# Synthesis and crystallographic characterization of di­phenyl­amide rare-earth metal complexes *Ln*(NPh_2_)_3_(THF)_2_ and [(Ph_2_N)_2_ *Ln*(μ-NPh_2_)]_2_

**DOI:** 10.1107/S2056989020009998

**Published:** 2020-08-14

**Authors:** Chad T. Palumbo, Christopher M. Kotyk, Joseph W. Ziller, William J. Evans

**Affiliations:** a Department of Chemistry, University of California, Irvine, CA 92697-2025, USA

**Keywords:** lanthanide, rare earth, di­phenyl­amide, oxide, crystal structure

## Abstract

Studies of the coordination chemistry between the di­phenyl­amide ligand, NPh_2_, and the smaller rare-earth *Ln* ^III^ ions, *Ln* = Y, Dy, and Er, led to the structural characterization by single-crystal X-ray diffraction crystallography of both solvated and unsolvated complexes, namely, tris­(di­phenyl­amido-κ*N*)bis­(tetrahydro­furan-κ*O*)yttrium(III), Y(NPh_2_)_3_(THF)_2_ or [Y(C_12_H_10_N)_3_(C_4_H_8_O)_2_], **1-Y**, and the erbium(III) (Er), **1-Er**, analogue, and bis­[μ-1κ*N*:2(η^6^)-di­phenyl­amido]­bis­[bis­(di­phenyl­amido-κ*N*)yttrium(III)], [(Ph_2_N)_2_Y(μ-NPh_2_)]_2_ or [Y_2_(C_12_H_10_N)_6_], **2-Y**, and the dysprosium(III) (Dy), **2-Dy**, analogue. The THF ligands of **1-Er** are modeled with disorder across two positions with occupancies of 0.627 (12):0.323 (12) and 0.633 (7):0.367 (7). Also structurally characterized was the tetra­metallic Er^III^ bridging oxide hydrolysis product, bis­(μ-di­phenyl­amido-κ^2^ *N*:*N*)bis­[μ-1κ*N*:2(η^6^)-di­phenyl­amido]­tetra­kis­(di­phenyl­amido-κ*N*)di-μ_3_-oxido-tetra­erbium(III) benzene disolvate, {[(Ph_2_N)Er(μ-NPh_2_)]_4_(μ-O)_2_}·(C_6_H_6_)_2_ or [Er_4_(C_12_H_10_N)_8_O_2_]·2C_6_H_6_, **3-Er**. The **3-Er** structure was refined as a three-component twin with occupancies 0.7375:0.2010:0.0615.

## Abstract 2

Crystallographic characterization of complexes of the smaller rare-earth elements Y, Dy, and Er with the NPh_2_ ligand reveals monometallic, bimetallic, and tetra­metallic structures.

## Chemical context   

Although the amide ligand, N*R*_2_, is widely used in rare-earth metal chemistry, most studies involve the bis­(tri­methyl­sil­yl)amide ligand originally introduced by Bradley, N(SiMe_3_)_2_ (Alyea *et al.*, 1972 ▸; Bradley *et al.*, 1972 ▸, 1973 ▸), and the di­methyl­silyl analog, N(SiHMe_2_)_2_ (Anwander *et al.*, 1998 ▸; Bienfait *et al.*, 2014 ▸; Meermann *et al.*, 2008 ▸), developed by Anwander. The neutral homoleptic complexes, *Ln*[N(SiMe_3_)_2_]_3_ and *Ln*[N(SiHMe_2_)_2_]_3_(THF)_2_, are heavily used in the rare-earth field.

In comparison, the NPh_2_ ligand has not been as extensively explored. The only neutral crystallographically characterized NPh_2_ rare-earth metal complexes in the literature are Yb(NPh_2_)_3_(THF)_2_ (**1-Yb**) (Yao *et al.*, 2001 ▸), Yb(NPh_2_)_3_[OP(NMe_2_)_3_]_2_ (Xu *et al.*, 2007 ▸), and [(Ph_2_N)_2_Ce(μ-NPh_2_)]_2_ (**2-Ce**) (Coles *et al.*, 2010 ▸). Many of the rare-earth NPh_2_ species are complex anions such as [*Ln*(NPh_2_)_4_]^1−^ (Yao *et al.*, 2004 ▸; Wong *et al.*, 1997*a* ▸,*b* ▸; Yu *et al.*, 2016 ▸), [*Ln*(NPh_2_)_4_]^2−^ (Minhas *et al.*, 1996 ▸), and [(C_5_H_4_ *R*)*Ln*(NPh_2_)_3_]^1−^ (*R* = Me, ^t^Bu) (Mao *et al.*, 1994 ▸).

To remedy the dearth of structural information on this class, we report the structures shown in the Scheme of the THF-solvated monometallic complexes *Ln*(NPh_2_)_3_(THF)_2_, **1-** ***Ln*** (*Ln* = Y, Er), the unsolvated bimetallic complexes [(Ph_2_N)_2_ *Ln*(μ-NPh_2_)]_2_, **2-** ***Ln*** (*Ln* = Y, Dy), and the tetra­metallic hydrolysis product {[(Ph_2_N)Er(μ-NPh_2_)]_4_(μ-O)_2_}·(C_6_H_6_)_2_, **3-Er**.
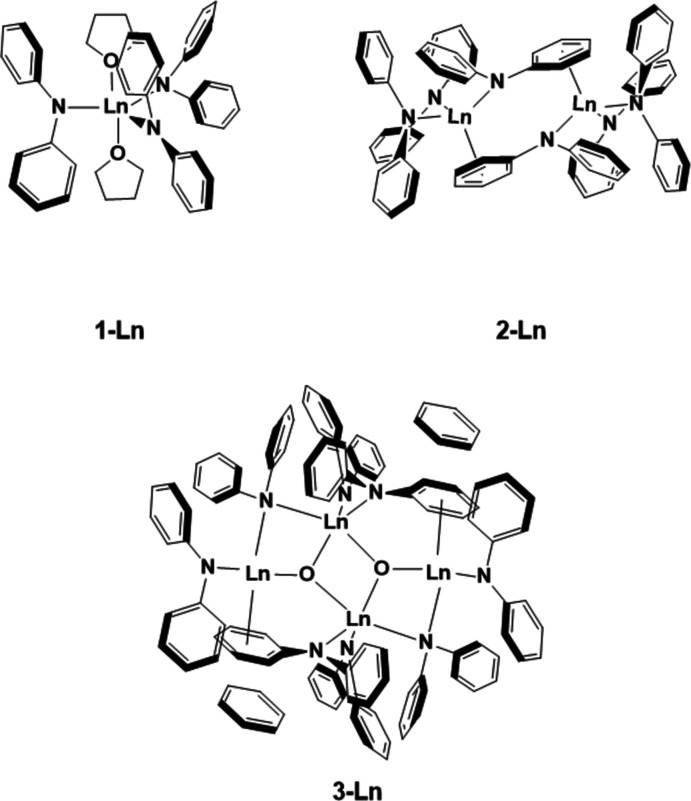


## Structural commentary   

**Figure 1:**
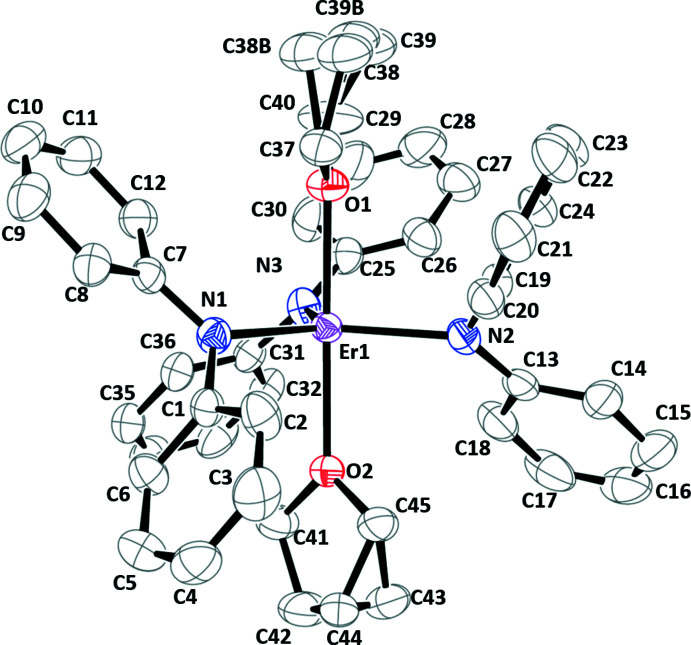
Displacement ellipsoid plot of Er(NPh_2_)_3_(THF)_2_, **1-Er**, drawn at the 50% probability level. Hydrogen atoms and co-crystallized solvent mol­ecules are omitted for clarity.

**Monometallic Complexes.** The metrical parameters of the *Ln*(NPh_2_)_3_(THF)_2_ complexes, **1-** ***Ln*** (*Ln* = Y, Er), are shown in Table 1 ▸ and the displacement ellipsoid plot of **1-Er** is shown in Fig. 1 ▸. The **1-** ***Ln*** complexes are not isomorphous; complex **1-Y** crystallizes in the *P*2_1_/*c* space group and **1-Er** in *P*2_1_/*n*. They contain five-coordinate *Ln* ^III^ ions with three amide and two neutral THF ligands arranged in a distorted trigonal–bipyramidal geometry. The divergence from perfect trigonal bipyramidal is evident by the three N(amide)—*Ln*1—N(amide) bond angles [**1-Y**: 130.61 (5), 122.03 (5), and 106.77 (5)°; **1-Er**: 130.04 (6), 119.13 (5), and 110.83 (6)°] that deviate from 120° and the O1—*Ln*1—O2 bond angles [**1-Y**: 160.31 (4)°; **1-Er**: 167.72 (4)°] that deviate from linearity. Complex **1-Y** has a *τ*_5_ parameter (Addison *et al.*, 1984 ▸) of 0.50 indicating a geometry halfway between ideal square-pyramidal (*τ*_5_ = 0) and trigonal–bipyramidal (*τ*_5_ = 1). The *τ*_5_ value of **1-Er** is 0.63 suggesting a geometry closer to trigonal–bipyramidal. The *Ln*—N(amide)_avg_ bond distances are 2.26 (2) Å for **1-Y** [Y1—N1 = 2.2612 (14), Y1—N2 = 2.2399 (14), Y1—N3 = 2.2870 (14) Å] and 2.25 (2) Å for **1-Er** [Er1—N1 = 2.2733 (15), Er1—N2 = 2.2524 (15), Er1—N3 = 2.2344 (15) Å], which reflects the similar size of these two ions [six-coordinate ionic radii: Y(III), 0.9 Å; Er(III), 0.89 Å] (Shannon, 1976 ▸). The *Ln*—O(THF)_avg_ bond lengths are 2.37 (2) Å for **1-Y** [Y1—O1 = 2.3526 (11), Y1—O2 = 2.3838 (12) Å] and 2.342 (6) Å for **1-Er** [Er1—O1 = 2.3475 (12), Er2—O2 = 2.3353 (11) Å].

**Figure 2:**
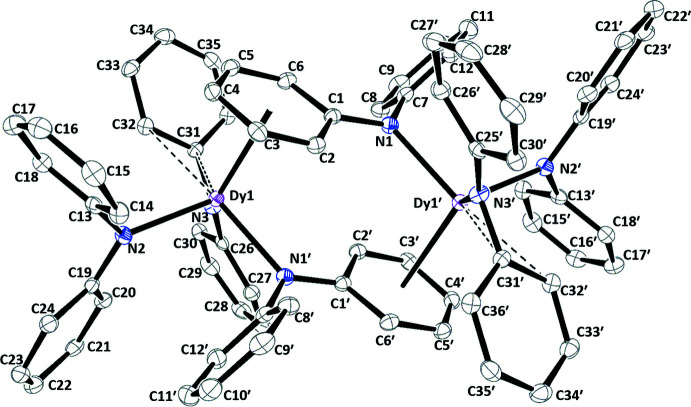
Displacement ellipsoid plot of [(Ph_2_N)_2_Dy(μ-NPh_2_)]_2_, **2-Dy**, drawn at the 50% probability level. Hydrogen atoms are omitted for clarity. The dashed lines represent the *Ln*—C(*ortho*) and *Ln*—C(*ipso*) distances discussed in the text. Symmetry code: (′) −*x* + 1, −*y* + 1, −*z* + 1.

**Bimetallic Complexes.** The metrical parameters of [(Ph_2_N)_2_ *Ln*(μ-NPh_2_)]_2_, **2-** ***Ln*** (*Ln* = Y, Dy), are presented in Table 2 ▸ and the displacement ellipsoid plot of **2-Dy** is in Fig. 2 ▸. The two **2-** ***Ln*** complexes (*Ln* = Y and Dy) are isomorphous and crystallize in the monoclinic *P*2_1_/*c* space group. Each mol­ecule of **2-** ***Ln*** is a dimer comprised of two (Ph_2_N)_2_ *Ln*(μ-NPh_2_) units that are related by an inversion center. The (μ-NPh_2_) ligand involving atom N1 binds to one lanthanide center through the nitro­gen atom and links to the other lanthanide center *via η* ^6^ coordination of one of the phenyl rings. The terminal NPh_2_ ligand involving N2 binds just through the nitro­gen donor atom. The other terminal NPh_2_ ligand containing N3 attaches to the *Ln* atom through the nitro­gen, but it also has a phenyl ring oriented toward the metal with *Ln*—C(*ipso*) and *Ln*—C(*ortho*) distances of 2.8235 (17) and 3.0169 (18) Å for Y and 2.836 (2) and 3.033 Å for Dy. These distances can be compared with the *Ln*1—N3 distances in these complexes: 2.2340 (15) Å for Y and 2.240 (2) Å for Dy. The bond distances of **2-Y** and **2-Dy** are close, which is consistent with their similar Shannon (1976 ▸) ionic radii [six-coordinate ionic radii: Y(III), 0.9 Å; Dy(III), 0.912 Å].

The 2.228 (2)–2.240 (2) Å range of terminal *Ln*—N(amide) bond distances in **2-** ***Ln*** is at the lower end of the 2.2343 (15)–2.2870 (14) Å range of distances in **1-** ***Ln*** and slightly shorter than the *Ln*—N1 distances of the bridging NPh_2_ [Y1—N1′ = 2.3039 (15) and Dy1—N1′ = 2.309 (2) Å], as is typical for terminal vs bridging ligands. The *Ln*—N2 distances [Y, 2.2294 (15) Å; Dy 2.228 (2) Å] are similar and comparable to the *Ln*—N1 bond lengths. The *Ln*—(phenyl ring centroid distances are also similar [Y, 2.584 Å; Dy, 2.605 Å] with similar *Ln*—C(phen­yl) bond distance ranges [Y, 2.8129 (19)–3.1300 (18) Å; Dy, 2.833 (2)–3.151 (3) Å].

**Figure 3:**
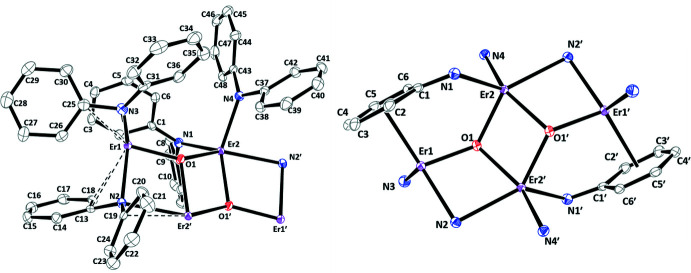
Displacement ellipsoid plots drawn at the 50% probability level of the asymmetric unit of {[(Ph_2_N)Er(μ-NPh_2_)]_4_(μ-O)_2_}·(C_6_H_6_)_2_, **3-Er**, with atoms Er1′, Er2′, O1′, N2′ added for clarity (left) and the Er_4_O_2_ core of **3-Er** (right). Hydrogen atoms and a mol­ecule of benzene in the asymmetric unit are omitted for clarity. Symmetry code: (′) −*x* + 1, −*y* + 1, −*z* + 1.

**A Tetra­metallic Complex.** The displacement ellipsoid plot and metrical parameters of {[(Ph_2_N)Er(μ-NPh_2_)]_4_(μ-O)_2_}·(C_6_H_6_)_2_, **3-Er**, are shown below in Figs. 3 ▸ and 4 ▸ and Table 3 ▸. Complex **3-Er** crystallizes in the triclinic *P* 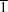 space group and is a tetra­metallic complex of Er^III^ comprised of two symmetrical {[(Ph_2_N)Er(μ-NPh_2_)]_2_(μ-O)}·(C_6_H_6_) units. The coordination environments of the two Er^III^ ions in this unit are different, as are all four NPh_2_ ligands. Er2 is five-coordinate with two μ-O bonds and three Er—N bonds. The bonding to Er1 is more complicated. It is bound to one μ-O ligand and one terminal NPh_2_ ligand through N3 with a short distance to *ipso* carbon C25. Er1 is also bound *η* ^6^ to a phenyl group of one μ-NPh_2_ ligand and to another μ-NPh_2_ ligand through the N2 atom that also bridges to Er2. In addition, C13 and C18 of this μ-NPh_2_ ligand are oriented toward Er1. The differences in the coord­ination environments of Er1 and Er2 lead to inequivalent Er—O bond distances [Er1—O1 = 2.095 (3), Er2—O1 = 2.190 (3) Å, Er2—O1′ = 2.245 (3) Å]. The Er–O–Er angle is bent [Er1—O1—Er2 = 133.25 (14)°]. The closest distances between the Er^III^ ions are Er1⋯Er2′ = 3.5734 (3) Å and Er2⋯Er2′ = 3.4836 (4) Å.

## Supra­molecular features   

**Figure 5:**
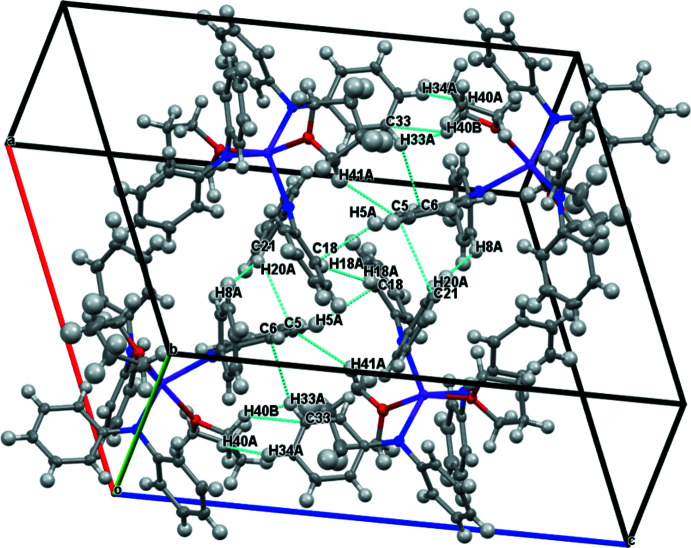
Packing structures and contacts for **1-Y**.

**Figure 6:**
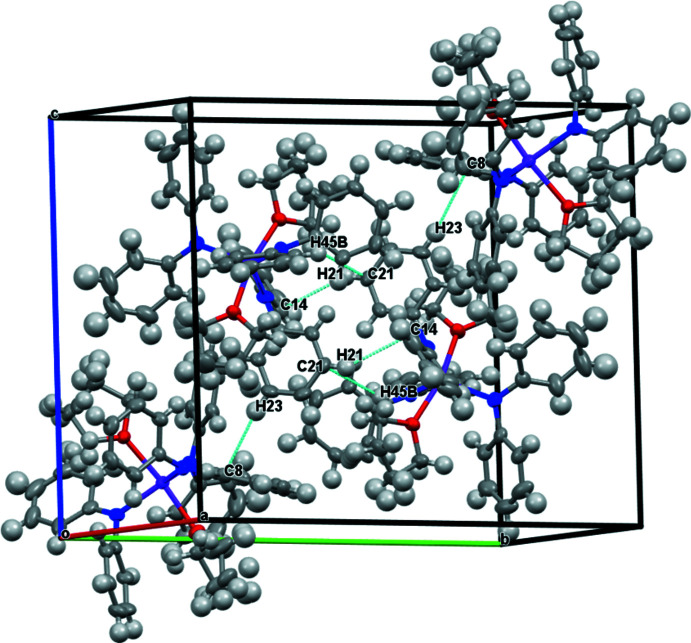
Packing structures and contacts for **1-Er**.

**Figure 7:**
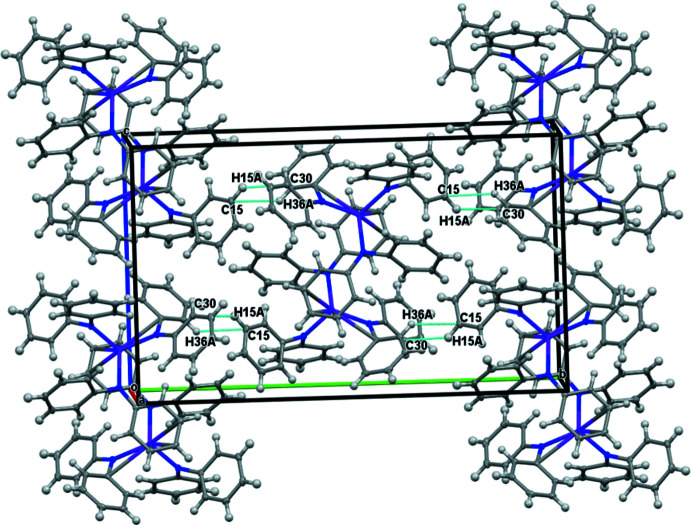
Packing structures and contacts for **2-*Ln***.

**Figure 8:**
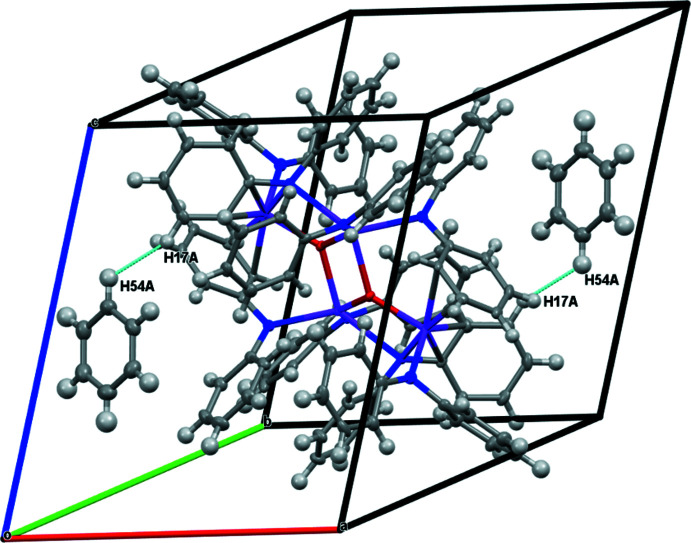
Packing structures and contacts for **3-Er**.

An examination of the packing diagrams for **1-** ***Ln*** (*Ln* = Y, Er), **2-** ***Ln*** (*Ln* = Y and Dy), and **3-Er** shows close C—H⋯phenyl inter­actions throughout the structures, Tables 4–7 ▸ ▸ ▸ ▸ and Figs. 5–8 ▸ ▸ ▸ ▸. Complex **3-Er** has two mol­ecules of benzene in the unit cell.

## Database survey   

A search of the Cambridge Structural Database shows **1-Yb** (Yao *et al.*, 2001 ▸) and **2-Ce** (Coles *et al.*, 2010 ▸) have been reported. Complex **1-Yb** is isomorphous with **1-Er**. Complex **2-Ce** is not isomorphous with **2-Y** and **2-Dy** and crystallizes in the *C*2/*c* space group. **2-Ce** is structurally different in that the μ-NPh_2_ ligand bridges the two Ce atoms only through the nitro­gen donor atom and not *via η* ^6^-phenyl-coordination as observed in **2-Y** and **2-Dy**. The tris­(amide) complex Yb(NPh_2_)_3_[OP(NMe_2_)_3_]_2_ (Xu *et al.*, 2007 ▸) has also been reported.

## Synthesis and crystallization   

**General Considerations.** All manipulations and syntheses described below were conducted with rigorous exclusion of air and water using standard Schlenk line and glovebox tech­niques under an argon atmosphere. Solvents were sparged with UHP argon (Airgas) and dried by passage through columns containing Q-5 and mol­ecular sieves prior to use. *Ln*Cl_3_ was prepared from the previously reported literature procedure (Meyer *et al.*, 1982 ▸). The compounds *Ln*[N(SiMe_3_)_2_]_3_ were prepared from their literature procedures (Bradley *et al.*, 1972 ▸). HNPh_2_ was purchased from commercial suppliers and used as received. NaNPh_2_ and KNPh_2_ were prepared by reaction of HNPh_2_ with NaH or KH in THF.

**Synthesis and Crystallization of Y(NPh_2_)_3_(THF)_2_, 1-Y.** In a glovebox, YCl_3_ (0.63 g, 3.2 mmol) was stirred for two days in THF (30 mL) in a Schlenk flask to ensure complete solvation. Under positive pressure of N_2_ on a Schlenk line, a solution of KNPh_2_ (1.9 g, 9.1 mmol) in THF (30 mL) was added dropwise to the YCl_3_ suspension in THF at 273 K over 15 min. The reaction vessel was allowed to warm to room temperature, and after 1 h, the solvent was removed under reduced pressure to yield a colorless solid. In a glovebox, the product was extracted with toluene and evaporated to dryness. The resulting solids were washed with hexane to yield **1-Y** as a colorless solid (2.2 g, 90%). The colorless solid was dissolved in diethyl ether and stored at 245 K for three days to yield colorless crystals of **1-Y**.

**Synthesis and Crystallization of Er(NPh_2_)_3_(THF)_2_, 1-Er.** In a glovebox, ErCl_3_ (243 mg, 0.887 mmol) was stirred in THF (10 mL), which gave a pink slurry. To the stirred suspension was added NaPh_2_ (500 mg, 2.62 mmol) in THF (10 mL) at 238 K dropwise over 5 min, and a color change to green–yellow and then pink was observed. After the addition, the resultant pink slurry was allowed to warm to room temperature and left to stir overnight. The volatiles were then removed under reduced pressure, which gave a pink gel. The gel was triturated with hexane several times to yield pink solids that were then dissolved in Et_2_O (17 mL) and stirred for several hours to ensure complete dissolution. Pink and colorless solids, presumably unreacted ErCl_3_ and NaNPh_2_, were centrifuged, and the volatiles of the supernatant were evaporated until supersaturation. As the concentrated pink solution warmed to room temperature, large pink hexa­gon-shaped crystals of Er(NPh_2_)_3_(THF)_2_, **1-Er**, suitable for X-ray diffraction grew within minutes (260 mg, 36%).

**Synthesis and Crystallization of [(NPh_2_)_2_Y(μ-NPh_2_)]_2_, 2-Y.** In a glovebox free of coordinating solvents, Y[N(SiMe_3_)_2_]_3_ (300 mg, 0.526 mmol) was dissolved in toluene (10 mL). To the stirred solution was added HNPh_2_ (272 mg, 1.61 mmol) in toluene (10 mL). The resultant colorless solution was left to stir for 48 h. The color of the solution slowly changed to yellow and a yellow precipitate was observed. The volatiles were removed under vacuum, and the resultant yellow solids were washed with hexane. The solids were stirred in benzene for 48 h, and the resultant yellow slurry was then centrifuged to remove the insoluble material. Toluene (4 mL) was added to the supernatant and the solution was concentrated to 4 mL before it was layered with hexane (15 mL). After 48 h at room temperature, yellow rectangular blocks of [(Ph_2_N)_2_Y(μ-NPh_2_)]_2_, **2-Y**, suitable for X-ray diffraction had formed.

**Synthesis and Crystallization of [(Ph_2_N)_2_Dy(μ-NPh_2_)]_2_, 2-Dy.** In a glovebox free of coordinating solvents, Dy[N(SiMe_3_)_2_]_3_ (300 mg, 0.466 mmol) was dissolved in toluene (10 mL). To the stirred solution was added HNPh_2_ (240 mg, 1.42 mmol) in toluene (10 mL). The resultant colorless solution was left to stir for 48 h and the color of the solution slowly turned to yellow and precipitated a yellow solid. The volatiles were removed, and the resultant yellow solids were washed with hexane. The solids were then stirred in benzene for 48 h, and the resultant yellow slurry was centrifuged to remove insoluble material. Toluene (4 mL) was added to the supernatant, and the solution was concentrated to 4 mL before it was layered with hexane (15 mL). After 48 h at room temperature, yellow rectangular blocks of [(Ph_2_N)_2_Dy(μ-NPh_2_)]_2_, **2-Dy**, suitable for X-ray diffraction had formed.

**Synthesis and Crystallization of {[(Ph_2_N)Er(μ-NPh_2_)]_4_(μ-O)_2_}·(C_6_H_6_)_2_, 3-Er.** In a glovebox free of coordinating solvents, Er[N(SiMe_3_)_2_]_3_ (300 mg, 0.463 mmol) was dissolved in toluene (10 mL). To the stirred solution was added HNPh_2_ (240 mg, 1.41 mmol) in toluene (10 mL). The resultant colorless solution was left to stir for 48 h, and the solution slowly changed color to yellow. The volatiles were removed, and the resultant yellow solids were washed with hexane. The solids were then stirred in benzene for 48 h, and the resultant yellow slurry was centrifuged to remove insoluble material. Toluene (4 mL) was added to the supernatant, and the solution was concentrated to 4 mL before it was layered with hexane (15 mL). After 48 h at room temperature, yellow rectangular blocks of {[(Ph_2_N)Er(μ-NPh_2_)]_4_(μ-O)_2_}·(C_6_H_6_)_2_, **3-Er**, suitable for X-ray diffraction had formed. Compound **3-Er** is a minor product of a formal hydrolysis of **2-Er**, presumably from adventitious water.

## Refinement   

**Refinement Details.** The mol­ecules of **2-** ***Ln*** and **3-Er** are located about an inversion center. There were two mol­ecules of benzene solvent present per empirical formula unit in **3-Er**. Crystal data, data collection and structure refinement details are summarized in Table 8 ▸. H atoms in all five structures were placed in calculated positions and C—H bond distances were constrained to 0.95 Å for aromatic and to 0.99 Å CH_2_ groups, respectively. *U*_iso_(H) values were set to 1.2*U*_eq_(C).

The two tetra­hydro­furan ligands in **1-Er** were modeled with disorder across two positions. For the ring of O1, two methyl­ene groups were included in the disorder, as well as the H atoms of the remaining CH_2_ groups. O—C bond distances were restrained to a target value of 1.47 (1) Å, C—C bond distances to a target value of 1.53 (1) Å. 1,3 distances between the oxygen atom and C38 and 39, and between C38*B* and C39*B* (*e.g*. the O—C—C angles) were restrained to be pairwise similar (with an esd of 0.02 Å). ADPs of the disordered carbon atoms (C38, C39, C38*B*, C39*B*) were constrained to be identical. *U* ^ij^ components of ADPs of atoms C39 and C40 were restrained to be similar with an esd of 0.01 Å^2^ and a distance cutoff of 4.0 Å. Subject to these conditions occupancies refined to 0.627 (12)/0.323 (12). For the ring involving O2, disorder was limited to one methyl­ene C atom and the H atoms of the two adjacent CH_2_ groups. No restraints were applied and occupancies refined to 0.633 (7)/0.367 (7).

The **3-Er** structure was found to be multi-component and was refined as a three-component twin. The orientation matrices for the three components were identified using the program *CELL_NOW* (Sheldrick, 2008*a* ▸). The second component is related to the first by no obvious twin law. The third component is related to the first by non-merohedry by a 180° rotation around [01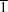]. The three components were integrated using *SAINT* (Bruker, 2013 ▸) and corrected for absorption using *TWINABS* (Sheldrick, 2012 ▸). The structure was solved using direct methods (Sheldrick 2008*b* ▸) with only the non-overlapping reflections of main component 1. The structure was refined using all reflections of component 1 (including the overlapping reflections), resulting in minor component occupancies of 0.0615 (6) and 0.2010 (4).

## Supplementary Material

Crystal structure: contains datablock(s) global, 1-Y, 1-Er, 2-Y, 2-Dy, 3-Er. DOI: 10.1107/S2056989020009998/zl2782sup1.cif

Structure factors: contains datablock(s) 1-Y. DOI: 10.1107/S2056989020009998/zl27821-Ysup7.hkl

Structure factors: contains datablock(s) 1-Er. DOI: 10.1107/S2056989020009998/zl27821-Ersup8.hkl

Structure factors: contains datablock(s) 2-Y. DOI: 10.1107/S2056989020009998/zl27822-Ysup9.hkl

Structure factors: contains datablock(s) 2-Dy. DOI: 10.1107/S2056989020009998/zl27822-Dysup10.hkl

Structure factors: contains datablock(s) 3-Er. DOI: 10.1107/S2056989020009998/zl27823-Ersup11.hkl

CCDC references: 2017774, 2017773, 2017772, 2017771, 2017770

Additional supporting information:  crystallographic information; 3D view; checkCIF report

## Figures and Tables

**Figure 4:**
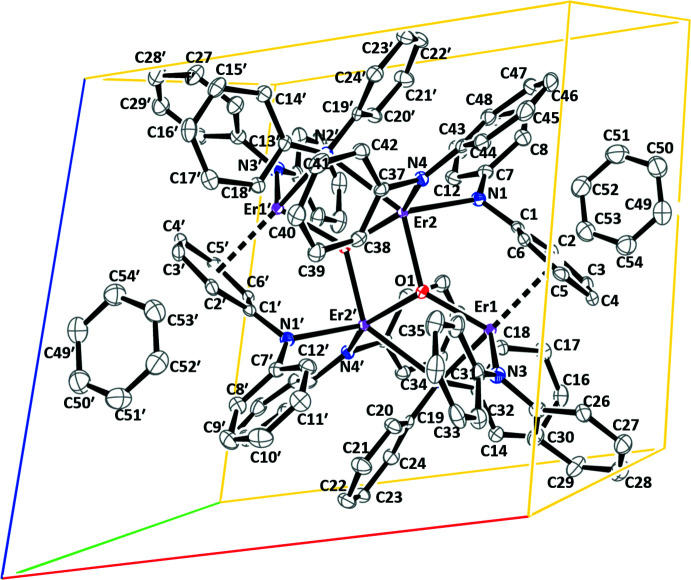
Unit-cell contents of {[(Ph_2_N)Er(μ-NPh_2_)]_4_(μ-O)_2_}·(C_6_H_6_)_2_, **3-Er**, with displacement ellipsoids drawn at the 50% probability level. Hydrogen atoms are omitted for clarity. Symmetry code: (′) −*x* + 1, −*y* + 1, −*z* + 1.

**Table 1:** Selected bond distances (Å) and angles (°) of *Ln*(NPh_2_)_3_(THF)_2_, **1-*Ln***

Parameter	**1-Y**	**1-Er**
*Ln*1—N1	2.2612 (14)	2.2733 (15)
*Ln*1—N2	2.2399 (14)	2.2524 (15)
*Ln*1—N3	2.2870 (14)	2.2344 (15)
*Ln*1—N(amide)­avg	2.26 (2)	2.25 (2)
*Ln*1—O1	2.3526 (11)	2.3475 (12)
*Ln*1—O2	2.3838 (12)	2.3353 (11)
*Ln*1—O(THF)avg	2.37 (2)	2.342 (6)
N1—*Ln*1—N2	106.77 (5)	130.04 (6)
N1—*Ln*1—N3	130.61 (5)	119.13 (5)
N2—*Ln*1—N3	122.03 (5)	110.83 (6)
O1—*Ln*1—O2	160.31 (4)	167.72 (4)

**Table 2:** Selected bond distances (Å) and angles (°) of [(Ph_2_N)_2_ *Ln*(μ-NPh_2_)]_2_, **2-*Ln***

Parameter	**2-Y**	**2-Dy**
*Ln*1—N1′	2.3039 (15)	2.309 (2)
*Ln*1—N2	2.2294 (15)	2.228 (2)
*Ln*1—N3	2.2340 (15)	2.240 (2)
*Ln*1—N(amide)­avg	2.25 (3)	2.26 (4)
*Ln*1—C1	3.1300 (18)	3.151 (2)
*Ln*1—C2	2.9498 (18)	2.967 (2)
*Ln*1—C3	2.8400 (18)	2.858 (2)
*Ln*1—C4	2.8129 (19)	2.833 (2)
*Ln*1—C5	2.8898 (18)	2.904 (3)
*Ln*1—C6	3.0125 (19)	3.032 (3)
*Ln*1—Centroid(phen­yl)	2.584	2.605
*Ln*1—C31	2.8235 (17)	2.836 (2)
*Ln*1—C32	3.0169 (18)	3.033

Symmetry code: (′) −*x* + 1, −*y* + 1, −*z* + 1.

**Table 3:** Selected bond distances (Å) and angles (°) of {[(Ph_2_N)Er(μ-NPh_2_)]_4_(μ-O)_2_}·(C_6_H_6_)_2_, **3-Er**

Parameter	**3-Er**
Er1—O1	2.095 (3)
Er2—O1	2.190 (3)
Er2—O1′	2.245 (3)
Er1—N2	2.367 (4)
Er1—N3	2.222 (3)
Er2—N1	2.303 (3)
Er2—N4	2.313 (4)
Er1—C1	2.871 (4)
Er1—C2	2.988 (4)
Er1—C3	2.989 (4)
Er1—C4	2.884 (4)
Er1—C5	2.784 (4)
Er1—C6	2.761 (4)
Er1—Centroid(phen­yl)	2.516
Er1—C13	2.812 (4)
Er1—C18	2.805 (4)
Er1—C25	2.904 (4)
Er2—C19′	2.903 (4)
Er1—O1—Er2	133.25 (14)
Er1—O1—Er2′	110.82 (12)
Er2—O1—Er2′	103.54 (11)
Er1⋯Er2′	3.5734 (3)
Er2⋯Er2′	3.4836 (4)

Symmetry code: (′) −*x* + 1, −*y* + 1, −*z* + 1.

**Table 4:** Inter­molecular contact lengths (Å) in **1-Y** vdW indicates the sum of the van der Waals radii of the two atoms.

Contact	**1-Y**	Length − vdW
C5⋯C21	3.388	−0.012
C5⋯H41*A*	2.840	−0.060
C6⋯H33*A*	2.890	−0.010
C18⋯H5*A*	2.822	−0.078
C33⋯H40*B*	2.825	−0.075
H8*A*⋯H20*A*	2.391	−0.009
H18*A*⋯H18*A*′	2.275	−0.125
H33*A*⋯H40*B*	2.271	−0.129
H34*A*⋯H40*A*	2.370	−0.030

**Table 5:** Inter­molecular contact lengths (Å) in **1-Er** vdW indicates the sum of the van der Waals radii of the two atoms.

Contact	**1-Er**	Length − vdW
C8⋯H23	2.873	−0.027
C14⋯H21	2.835	−0.065
C21⋯H45*B*	2.897	−0.003

**Table 6:** Inter­molecular contact lengths (Å) in **2-Y** and **2-Dy**

Contact	**2-Y**	Length − vdW	**2-Dy**	Length − vdW
C15⋯H36*A*	2.749	−0.151	2.737	−0.163
C30⋯H15*A*	2.805	−0.095	2.800	−0.100

vdW indicates the sum of the van der Waals radii of the two atoms.

**Table 7:** Inter­molecular contact lengths (Å) in **3-Er** vdW indicates the sum of the van der Waals radii of the two atoms.

Contact	**3-Er**	Length − vdW
H17*A*⋯H54*A*	2.370	−0.030

**Table 8:** Experimental details

	**1-Y**	**1-Er**	**2-Y**	**2-Dy**	**3-Er**
Crystal data					
Chemical formula	[Y(C_12_H_10_N)_3_(C_4_H_8_O)_2_]	[Er(C_12_H_10_N)_3_(C_4_H_8_O)_2_]	[Y_2_(C_12_H_10_N)_6_]	[Dy_2_(C_12_H_10_N)_6_]	[Er_4_(C_12_H_10_N)_8_O_2_]·2C_6_H_6_
*M*_r_	737.75	816.10	1187.08	1334.26	2202.93
Crystal system, space group	Monoclinic, *P*2_1_/*c*	Monoclinic, *P*2_1_/*n*	Monoclinic, *P*2_1_/*c*	Monoclinic, *P*2_1_/*c*	Triclinic, *P* 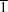
Temperature (K)	143	173	88	88	88
*a*, *b*, *c* (Å)	15.3539 (9), 12.5259 (7), 20.2511 (12)	12.0946 (5), 19.1086 (8), 16.3609 (7)	9.2776 (5), 22.5591 (13), 13.4791 (8)	9.3068 (15), 22.475 (4), 13.513 (2)	12.8857 (8), 13.6846 (9), 13.7411 (9)
α, β, γ (°)	90, 107.207 (1), 90	90, 91.3697 (5), 90	90, 91.4966 (9), 90	90, 91.266 (2), 90	61.3447 (8), 82.7796 (10), 83.0804 (10)
*V* (Å^3^)	3720.4 (4)	3780.1 (3)	2820.1 (3)	2825.8 (8)	2104.4 (2)
*Z*	4	4	2	2	1
Radiation type	Mo *K*α	Mo *K*α	Mo *K*α	Mo *K*α	Mo *K*α
μ (mm^−1^)	1.61	2.26	2.10	2.67	4.01
Crystal size (mm)	0.52 × 0.38 × 0.37	0.48 × 0.39 × 0.33	0.28 × 0.24 × 0.15	0.20 × 0.12 × 0.11	0.35 × 0.28 × 0.11

Data collection					
Diffractometer	Bruker SMART APEXII CCD	Bruker SMART APEXII CCD	Bruker SMART APEXII CCD	Bruker SMART APEXII CCD	Bruker SMART APEXII CCD
Absorption correction	Numerical (*SADABS*; Krause *et al*, 2015 ▸)	Multi-scan (*SADABS*; Sheldrick, 2014*b* ▸)	Multi-scan (*SADABS*; Krause *et al*, 2015 ▸)	Multi-scan (*SADABS*; Krause *et al*, 2015 ▸)	Multi-scan (*TWINABS*; Sheldrick, 2012 ▸)
*T*_min_, *T*_max_	0.537, 0.683	0.557, 0.695	0.622, 0.746	0.637, 0.746	0.254, 0.432
No. of measured, independent and observed [*I* > 2σ(*I*)] reflections	42053, 8829, 7562	46529, 9687, 8834	22940, 6851, 5604	34856, 7264, 6207	51658, 10308, 9209
*R*_int_	0.025	0.018	0.037	0.037	0.052

Refinement					
*R*[*F* ^2^ > 2σ(*F* ^2^)], *wR*(*F* ^2^), *S*	0.031, 0.081, 1.04	0.019, 0.050, 1.04	0.032, 0.070, 1.03	0.026, 0.065, 1.05	0.027, 0.062, 0.96
No. of reflections	8829	9687	6851	7264	10308
No. of parameters	451	462	361	361	552
No. of restraints	0	28	0	0	0
H-atom treatment	H-atom parameters constrained	H-atom parameters constrained	H-atom parameters constrained	H-atom parameters constrained	H-atom parameters constrained
Δρ_max_, Δρ_min_ (e Å^−3^)	0.81, −0.47	0.70, −0.42	0.39, −0.35	2.65, −0.81	1.62, −1.10

Computer programs: *APEX2* (Bruker, 2011, 2014 ▸), *SAINT* (Bruker, 2009, 2013 ▸), *SHELXS* (Sheldrick, 2008*b* ▸), *SHELXT* (Sheldrick, 2015*a* ▸), *SHELXL2014/7* (Sheldrick, 2015*b* ▸) and *SHELXTL* (Sheldrick, 2008*b* ▸).

## supplementary crystallographic information

### Tris(diphenylamido-κN)bis(tetrahydrofuran-κO)yttrium(III) (1-Y) Crystal data

**Table:**

[Y(C_12_H_10_N)_3_(C_4_H_8_O)_2_]	*F*(000) = 1544
*M**_r_* = 737.75	*D*_x_ = 1.317 Mg m^−^^3^
Monoclinic, *P*2_1_/*c*	Mo *K*α radiation, λ = 0.71073 Å
*a* = 15.3539 (9) Å	Cell parameters from 9981 reflections
*b* = 12.5259 (7) Å	θ = 2.2–28.2°
*c* = 20.2511 (12) Å	µ = 1.61 mm^−^^1^
β = 107.207 (1)°	*T* = 143 K
*V* = 3720.4 (4) Å^3^	Irregular, colorless
*Z* = 4	0.52 × 0.38 × 0.37 mm

### Tris(diphenylamido-κN)bis(tetrahydrofuran-κO)yttrium(III) (1-Y) Data collection

**Table:**

Bruker SMART APEXII CCD diffractometer	7562 reflections with *I* > 2σ(*I*)
Radiation source: fine-focus sealed tube	*R*_int_ = 0.025
φ and ω scans	θ_max_ = 28.5°, θ_min_ = 1.4°
Absorption correction: numerical (SADABS; Krause et al., 2015)	*h* = −20→19
*T*_min_ = 0.537, *T*_max_ = 0.683	*k* = −16→16
42053 measured reflections	*l* = −25→26
8829 independent reflections	

### Tris(diphenylamido-κN)bis(tetrahydrofuran-κO)yttrium(III) (1-Y) Refinement

**Table:**

Refinement on *F*^2^	Primary atom site location: structure-invariant direct methods
Least-squares matrix: full	Secondary atom site location: difference Fourier map
*R*[*F*^2^ > 2σ(*F*^2^)] = 0.031	Hydrogen site location: inferred from neighbouring sites
*wR*(*F*^2^) = 0.081	H-atom parameters constrained
*S* = 1.03	*w* = 1/[σ^2^(*F*_o_^2^) + (0.044*P*)^2^ + 1.6736*P*] where *P* = (*F*_o_^2^ + 2*F*_c_^2^)/3
8829 reflections	(Δ/σ)_max_ = 0.001
451 parameters	Δρ_max_ = 0.81 e Å^−^^3^
0 restraints	Δρ_min_ = −0.46 e Å^−^^3^

### Tris(diphenylamido-κN)bis(tetrahydrofuran-κO)yttrium(III) (1-Y) Special details

**Table:**

Geometry. All esds (except the esd in the dihedral angle between two l.s. planes) are estimated using the full covariance matrix. The cell esds are taken into account individually in the estimation of esds in distances, angles and torsion angles; correlations between esds in cell parameters are only used when they are defined by crystal symmetry. An approximate (isotropic) treatment of cell esds is used for estimating esds involving l.s. planes.
Refinement. A colorless crystal of approximate dimensions 0.373 x 0.377 x 0.520 mm was mounted on a glass fiber and transferred to a Bruker SMART APEX II diffractometer. The APEX2 program package was used to determine the unit-cell parameters and for data collection (15 sec/frame scan time for a sphere of diffraction data). The raw frame data was processed using SAINT and SADABS to yield the reflection data file. Subsequent calculations were carried out using the SHELXTL program. The diffraction symmetry was 2/m and the systematic absences were consistent with the monoclinic space group P21/c that was later determined to be correct.The structure was solved by direct methods and refined on F2 by full-matrix least-squares techniques. The analytical scattering factors for neutral atoms were used throughout the analysis. Hydrogen atoms were included using a riding model.Least-squares analysis yielded wR2 = 0.0812 and Goof = 1.035 for 451 variables refined against 8829 data (0.75Å), R1 = 0.0313 for those 7562 data with I > 2.0sigma(I).

### Tris(diphenylamido-κN)bis(tetrahydrofuran-κO)yttrium(III) (1-Y) Fractional atomic coordinates and isotropic or equivalent isotropic displacement parameters (Å^2^)

**Table:**

	*x*	*y*	*z*	*U*_iso_*/*U*_eq_	
Y1	0.25279 (2)	0.20035 (2)	0.12211 (2)	0.01710 (5)	
O1	0.20503 (8)	0.07834 (9)	0.19266 (6)	0.0202 (2)	
O2	0.26567 (8)	0.36154 (9)	0.06312 (6)	0.0246 (3)	
N1	0.30919 (10)	0.28942 (11)	0.22288 (7)	0.0218 (3)	
N2	0.37155 (9)	0.12258 (11)	0.09853 (7)	0.0221 (3)	
N3	0.11008 (9)	0.20102 (11)	0.04461 (7)	0.0211 (3)	
C1	0.36370 (11)	0.24655 (14)	0.28472 (9)	0.0219 (3)	
C2	0.40627 (12)	0.14663 (14)	0.28461 (9)	0.0258 (4)	
H2A	0.4010	0.1121	0.2418	0.031*	
C3	0.45564 (12)	0.09783 (15)	0.34571 (10)	0.0308 (4)	
H3A	0.4824	0.0299	0.3441	0.037*	
C4	0.46648 (14)	0.14671 (18)	0.40897 (10)	0.0363 (5)	
H4A	0.4997	0.1127	0.4507	0.044*	
C5	0.42786 (13)	0.24610 (18)	0.41004 (10)	0.0340 (4)	
H5A	0.4363	0.2811	0.4531	0.041*	
C6	0.37708 (12)	0.29590 (15)	0.34973 (9)	0.0261 (4)	
H6A	0.3510	0.3640	0.3522	0.031*	
C7	0.28589 (12)	0.39899 (13)	0.22410 (8)	0.0219 (3)	
C8	0.35150 (12)	0.47838 (14)	0.22855 (9)	0.0258 (4)	
H8A	0.4126	0.4583	0.2330	0.031*	
C9	0.32903 (14)	0.58517 (15)	0.22662 (10)	0.0326 (4)	
H9A	0.3747	0.6379	0.2304	0.039*	
C10	0.23979 (15)	0.61557 (16)	0.21918 (11)	0.0364 (4)	
H10A	0.2240	0.6891	0.2175	0.044*	
C11	0.17374 (14)	0.53803 (16)	0.21426 (11)	0.0344 (4)	
H11A	0.1125	0.5585	0.2090	0.041*	
C12	0.19682 (12)	0.43051 (15)	0.21696 (9)	0.0260 (4)	
H12A	0.1512	0.3780	0.2139	0.031*	
C13	0.46178 (11)	0.15543 (14)	0.10648 (8)	0.0223 (3)	
C14	0.49507 (12)	0.24840 (15)	0.14365 (10)	0.0280 (4)	
H14A	0.4591	0.2842	0.1677	0.034*	
C15	0.58011 (13)	0.28907 (15)	0.14586 (12)	0.0345 (4)	
H15A	0.6012	0.3528	0.1709	0.041*	
C16	0.63410 (13)	0.23818 (17)	0.11216 (11)	0.0356 (4)	
H16A	0.6915	0.2674	0.1129	0.043*	
C17	0.60379 (13)	0.14390 (18)	0.07720 (10)	0.0353 (4)	
H17A	0.6413	0.1074	0.0547	0.042*	
C18	0.51904 (12)	0.10234 (16)	0.07473 (9)	0.0295 (4)	
H18A	0.4996	0.0370	0.0512	0.035*	
C19	0.35113 (11)	0.01295 (14)	0.09203 (8)	0.0214 (3)	
C20	0.40967 (12)	−0.06562 (15)	0.13115 (9)	0.0260 (4)	
H20A	0.4685	−0.0457	0.1596	0.031*	
C21	0.38309 (13)	−0.17088 (16)	0.12880 (10)	0.0301 (4)	
H21A	0.4238	−0.2225	0.1556	0.036*	
C22	0.29726 (13)	−0.20271 (15)	0.08766 (10)	0.0303 (4)	
H22A	0.2790	−0.2753	0.0867	0.036*	
C23	0.23913 (12)	−0.12732 (15)	0.04830 (9)	0.0270 (4)	
H23A	0.1806	−0.1484	0.0199	0.032*	
C24	0.26538 (11)	−0.02058 (14)	0.04980 (8)	0.0230 (3)	
H24A	0.2248	0.0301	0.0219	0.028*	
C25	0.03547 (11)	0.18946 (14)	0.07233 (8)	0.0206 (3)	
C26	0.00257 (11)	0.08786 (14)	0.08106 (9)	0.0243 (3)	
H26A	0.0271	0.0269	0.0650	0.029*	
C27	−0.06539 (12)	0.07479 (16)	0.11287 (9)	0.0292 (4)	
H27A	−0.0867	0.0052	0.1187	0.035*	
C28	−0.10230 (12)	0.16307 (18)	0.13626 (9)	0.0313 (4)	
H28A	−0.1485	0.1542	0.1583	0.038*	
C29	−0.07127 (13)	0.26385 (17)	0.12718 (9)	0.0308 (4)	
H29A	−0.0967	0.3245	0.1428	0.037*	
C30	−0.00299 (12)	0.27746 (15)	0.09538 (9)	0.0253 (4)	
H30A	0.0175	0.3473	0.0893	0.030*	
C31	0.08840 (11)	0.20715 (13)	−0.02667 (8)	0.0205 (3)	
C32	0.15870 (12)	0.20298 (13)	−0.05869 (9)	0.0225 (3)	
H32A	0.2202	0.1972	−0.0306	0.027*	
C33	0.14032 (13)	0.20708 (14)	−0.12980 (9)	0.0266 (4)	
H33A	0.1891	0.2032	−0.1497	0.032*	
C34	0.05132 (13)	0.21681 (15)	−0.17243 (9)	0.0297 (4)	
H34A	0.0386	0.2191	−0.2213	0.036*	
C35	−0.01811 (12)	0.22302 (16)	−0.14231 (9)	0.0293 (4)	
H35A	−0.0791	0.2309	−0.1710	0.035*	
C36	−0.00112 (12)	0.21802 (14)	−0.07121 (9)	0.0251 (4)	
H36A	−0.0506	0.2220	−0.0522	0.030*	
C37	0.22126 (13)	−0.03600 (14)	0.20410 (9)	0.0269 (4)	
H37A	0.2846	−0.0545	0.2059	0.032*	
H37B	0.1787	−0.0778	0.1667	0.032*	
C38	0.20468 (13)	−0.05804 (15)	0.27300 (9)	0.0279 (4)	
H38A	0.2596	−0.0425	0.3121	0.033*	
H38B	0.1859	−0.1330	0.2762	0.033*	
C39	0.12761 (12)	0.01922 (14)	0.27130 (9)	0.0255 (4)	
H39A	0.0683	−0.0080	0.2418	0.031*	
H39B	0.1232	0.0332	0.3183	0.031*	
C40	0.15694 (12)	0.11810 (14)	0.24052 (9)	0.0228 (3)	
H40A	0.1033	0.1613	0.2156	0.027*	
H40B	0.1979	0.1627	0.2771	0.027*	
C41	0.33694 (14)	0.39496 (18)	0.03414 (11)	0.0395 (5)	
H41A	0.3666	0.3323	0.0202	0.047*	
H41B	0.3838	0.4372	0.0682	0.047*	
C42	0.28975 (18)	0.4624 (3)	−0.02798 (16)	0.0653 (8)	
H42A	0.3265	0.5265	−0.0302	0.078*	
H42B	0.2804	0.4210	−0.0712	0.078*	
C43	0.1999 (2)	0.4936 (2)	−0.01854 (13)	0.0580 (7)	
H43A	0.1946	0.5723	−0.0171	0.070*	
H43B	0.1490	0.4659	−0.0571	0.070*	
C44	0.19771 (14)	0.44599 (15)	0.04797 (11)	0.0348 (4)	
H44A	0.2126	0.5004	0.0851	0.042*	
H44B	0.1365	0.4165	0.0439	0.042*	

### Tris(diphenylamido-κN)bis(tetrahydrofuran-κO)yttrium(III) (1-Y) Atomic displacement parameters (Å^2^)

**Table:**

	*U*^11^	*U*^22^	*U*^33^	*U*^12^	*U*^13^	*U*^23^
Y1	0.01599 (8)	0.01943 (8)	0.01551 (8)	0.00072 (6)	0.00409 (6)	0.00039 (6)
O1	0.0236 (6)	0.0206 (6)	0.0189 (5)	0.0012 (5)	0.0100 (5)	−0.0002 (4)
O2	0.0274 (6)	0.0235 (6)	0.0228 (6)	0.0006 (5)	0.0076 (5)	0.0042 (5)
N1	0.0236 (7)	0.0207 (7)	0.0197 (7)	0.0000 (6)	0.0040 (6)	−0.0010 (5)
N2	0.0190 (7)	0.0238 (7)	0.0221 (7)	0.0019 (6)	0.0041 (6)	−0.0032 (6)
N3	0.0174 (6)	0.0275 (7)	0.0187 (7)	0.0003 (6)	0.0059 (5)	0.0004 (6)
C1	0.0192 (8)	0.0244 (9)	0.0218 (8)	−0.0053 (7)	0.0055 (6)	−0.0004 (7)
C2	0.0236 (8)	0.0245 (9)	0.0274 (9)	−0.0025 (7)	0.0044 (7)	−0.0004 (7)
C3	0.0243 (9)	0.0282 (9)	0.0368 (10)	−0.0006 (7)	0.0044 (8)	0.0083 (8)
C4	0.0318 (10)	0.0459 (12)	0.0276 (10)	−0.0007 (9)	0.0030 (8)	0.0133 (9)
C5	0.0334 (10)	0.0484 (12)	0.0207 (9)	−0.0068 (9)	0.0088 (8)	0.0006 (8)
C6	0.0250 (9)	0.0312 (9)	0.0228 (8)	−0.0042 (7)	0.0082 (7)	−0.0002 (7)
C7	0.0270 (8)	0.0225 (8)	0.0149 (7)	0.0001 (7)	0.0043 (6)	−0.0022 (6)
C8	0.0247 (8)	0.0261 (9)	0.0241 (8)	−0.0012 (7)	0.0034 (7)	−0.0017 (7)
C9	0.0369 (10)	0.0240 (9)	0.0338 (10)	−0.0061 (8)	0.0057 (8)	−0.0016 (8)
C10	0.0471 (12)	0.0221 (9)	0.0401 (11)	0.0054 (8)	0.0129 (9)	−0.0034 (8)
C11	0.0329 (10)	0.0356 (10)	0.0362 (10)	0.0084 (8)	0.0127 (8)	−0.0027 (8)
C12	0.0267 (9)	0.0278 (9)	0.0242 (9)	−0.0013 (7)	0.0087 (7)	−0.0013 (7)
C13	0.0192 (8)	0.0280 (8)	0.0192 (8)	0.0031 (7)	0.0046 (6)	0.0025 (7)
C14	0.0226 (8)	0.0264 (9)	0.0340 (10)	0.0041 (7)	0.0070 (7)	−0.0011 (8)
C15	0.0254 (9)	0.0272 (10)	0.0477 (12)	−0.0003 (7)	0.0058 (9)	0.0023 (8)
C16	0.0225 (9)	0.0398 (11)	0.0455 (12)	−0.0020 (8)	0.0115 (8)	0.0089 (9)
C17	0.0280 (9)	0.0478 (12)	0.0342 (10)	0.0036 (9)	0.0155 (8)	0.0013 (9)
C18	0.0276 (9)	0.0384 (10)	0.0241 (9)	−0.0007 (8)	0.0101 (7)	−0.0045 (8)
C19	0.0210 (8)	0.0270 (8)	0.0183 (7)	0.0022 (7)	0.0091 (6)	−0.0027 (6)
C20	0.0205 (8)	0.0322 (9)	0.0250 (9)	0.0050 (7)	0.0062 (7)	−0.0006 (7)
C21	0.0290 (9)	0.0309 (9)	0.0314 (10)	0.0087 (8)	0.0106 (8)	0.0037 (8)
C22	0.0341 (10)	0.0268 (9)	0.0335 (10)	−0.0009 (8)	0.0154 (8)	−0.0013 (8)
C23	0.0245 (8)	0.0358 (10)	0.0224 (8)	−0.0056 (7)	0.0094 (7)	−0.0040 (7)
C24	0.0203 (8)	0.0311 (9)	0.0182 (8)	0.0028 (7)	0.0065 (6)	0.0000 (7)
C25	0.0157 (7)	0.0289 (9)	0.0158 (7)	0.0015 (6)	0.0024 (6)	0.0006 (6)
C26	0.0216 (8)	0.0270 (9)	0.0227 (8)	0.0012 (7)	0.0041 (7)	0.0002 (7)
C27	0.0240 (9)	0.0353 (10)	0.0263 (9)	−0.0050 (7)	0.0044 (7)	0.0059 (8)
C28	0.0218 (8)	0.0517 (12)	0.0217 (9)	0.0024 (8)	0.0084 (7)	0.0080 (8)
C29	0.0296 (9)	0.0387 (10)	0.0263 (9)	0.0105 (8)	0.0116 (8)	0.0008 (8)
C30	0.0252 (8)	0.0268 (9)	0.0240 (8)	0.0036 (7)	0.0077 (7)	0.0025 (7)
C31	0.0209 (8)	0.0201 (8)	0.0197 (8)	−0.0004 (6)	0.0046 (6)	0.0021 (6)
C32	0.0193 (8)	0.0254 (8)	0.0223 (8)	−0.0013 (7)	0.0053 (6)	0.0015 (7)
C33	0.0276 (9)	0.0309 (9)	0.0231 (8)	−0.0045 (7)	0.0102 (7)	0.0022 (7)
C34	0.0339 (10)	0.0354 (10)	0.0178 (8)	−0.0076 (8)	0.0043 (7)	0.0058 (7)
C35	0.0229 (8)	0.0358 (10)	0.0242 (9)	−0.0045 (7)	−0.0007 (7)	0.0077 (7)
C36	0.0189 (8)	0.0308 (9)	0.0252 (9)	−0.0014 (7)	0.0059 (7)	0.0050 (7)
C37	0.0379 (10)	0.0193 (8)	0.0288 (9)	0.0014 (7)	0.0177 (8)	0.0011 (7)
C38	0.0338 (9)	0.0285 (9)	0.0249 (9)	0.0053 (8)	0.0141 (8)	0.0060 (7)
C39	0.0279 (9)	0.0295 (9)	0.0223 (8)	0.0007 (7)	0.0125 (7)	0.0026 (7)
C40	0.0271 (8)	0.0245 (8)	0.0203 (8)	0.0019 (7)	0.0124 (7)	−0.0028 (7)
C41	0.0285 (10)	0.0456 (12)	0.0454 (12)	−0.0038 (9)	0.0126 (9)	0.0200 (10)
C42	0.0558 (15)	0.0785 (19)	0.0701 (18)	0.0182 (14)	0.0319 (14)	0.0487 (16)
C43	0.0764 (18)	0.0595 (16)	0.0433 (13)	0.0318 (14)	0.0260 (13)	0.0228 (12)
C44	0.0399 (11)	0.0259 (9)	0.0412 (11)	0.0095 (8)	0.0161 (9)	0.0088 (8)

### Tris(diphenylamido-κN)bis(tetrahydrofuran-κO)yttrium(III) (1-Y) Geometric parameters (Å, º)

**Table:**

Y1—N2	2.2399 (14)	C21—C22	1.393 (3)
Y1—N1	2.2612 (14)	C21—H21A	0.9500
Y1—N3	2.2870 (14)	C22—C23	1.379 (3)
Y1—O1	2.3526 (11)	C22—H22A	0.9500
Y1—O2	2.3838 (12)	C23—C24	1.394 (3)
O1—C37	1.460 (2)	C23—H23A	0.9500
O1—C40	1.4682 (18)	C24—H24A	0.9500
O2—C41	1.448 (2)	C25—C30	1.394 (2)
O2—C44	1.453 (2)	C25—C26	1.400 (2)
N1—C1	1.392 (2)	C26—C27	1.389 (2)
N1—C7	1.420 (2)	C26—H26A	0.9500
N2—C19	1.406 (2)	C27—C28	1.388 (3)
N2—C13	1.408 (2)	C27—H27A	0.9500
N3—C31	1.384 (2)	C28—C29	1.381 (3)
N3—C25	1.425 (2)	C28—H28A	0.9500
C1—C2	1.412 (2)	C29—C30	1.394 (3)
C1—C6	1.413 (2)	C29—H29A	0.9500
C2—C3	1.387 (3)	C30—H30A	0.9500
C2—H2A	0.9500	C31—C36	1.410 (2)
C3—C4	1.385 (3)	C31—C32	1.415 (2)
C3—H3A	0.9500	C32—C33	1.384 (2)
C4—C5	1.382 (3)	C32—H32A	0.9500
C4—H4A	0.9500	C33—C34	1.389 (3)
C5—C6	1.388 (3)	C33—H33A	0.9500
C5—H5A	0.9500	C34—C35	1.378 (3)
C6—H6A	0.9500	C34—H34A	0.9500
C7—C12	1.389 (2)	C35—C36	1.387 (2)
C7—C8	1.399 (2)	C35—H35A	0.9500
C8—C9	1.379 (3)	C36—H36A	0.9500
C8—H8A	0.9500	C37—C38	1.516 (2)
C9—C10	1.387 (3)	C37—H37A	0.9900
C9—H9A	0.9500	C37—H37B	0.9900
C10—C11	1.386 (3)	C38—C39	1.521 (2)
C10—H10A	0.9500	C38—H38A	0.9900
C11—C12	1.390 (3)	C38—H38B	0.9900
C11—H11A	0.9500	C39—C40	1.514 (2)
C12—H12A	0.9500	C39—H39A	0.9900
C13—C14	1.399 (3)	C39—H39B	0.9900
C13—C18	1.401 (2)	C40—H40A	0.9900
C14—C15	1.390 (3)	C40—H40B	0.9900
C14—H14A	0.9500	C41—C42	1.510 (3)
C15—C16	1.376 (3)	C41—H41A	0.9900
C15—H15A	0.9500	C41—H41B	0.9900
C16—C17	1.385 (3)	C42—C43	1.499 (4)
C16—H16A	0.9500	C42—H42A	0.9900
C17—C18	1.389 (3)	C42—H42B	0.9900
C17—H17A	0.9500	C43—C44	1.483 (3)
C18—H18A	0.9500	C43—H43A	0.9900
C19—C24	1.405 (2)	C43—H43B	0.9900
C19—C20	1.408 (2)	C44—H44A	0.9900
C20—C21	1.377 (3)	C44—H44B	0.9900
C20—H20A	0.9500		
			
N2—Y1—N1	106.77 (5)	C23—C22—H22A	120.5
N2—Y1—N3	122.03 (5)	C21—C22—H22A	120.5
N1—Y1—N3	130.61 (5)	C22—C23—C24	120.69 (17)
N2—Y1—O1	105.47 (5)	C22—C23—H23A	119.7
N1—Y1—O1	82.96 (5)	C24—C23—H23A	119.7
N3—Y1—O1	90.67 (5)	C23—C24—C19	120.84 (16)
N2—Y1—O2	93.90 (5)	C23—C24—H24A	119.6
N1—Y1—O2	88.17 (5)	C19—C24—H24A	119.6
N3—Y1—O2	81.81 (5)	C30—C25—C26	118.17 (16)
O1—Y1—O2	160.31 (4)	C30—C25—N3	121.41 (15)
C37—O1—C40	108.88 (12)	C26—C25—N3	120.30 (15)
C37—O1—Y1	131.62 (9)	C27—C26—C25	120.97 (17)
C40—O1—Y1	119.24 (9)	C27—C26—H26A	119.5
C41—O2—C44	106.58 (14)	C25—C26—H26A	119.5
C41—O2—Y1	129.24 (11)	C28—C27—C26	120.20 (18)
C44—O2—Y1	124.16 (10)	C28—C27—H27A	119.9
C1—N1—C7	116.41 (13)	C26—C27—H27A	119.9
C1—N1—Y1	126.11 (11)	C29—C28—C27	119.41 (17)
C7—N1—Y1	117.47 (10)	C29—C28—H28A	120.3
C19—N2—C13	118.74 (14)	C27—C28—H28A	120.3
C19—N2—Y1	105.76 (10)	C28—C29—C30	120.65 (18)
C13—N2—Y1	133.16 (11)	C28—C29—H29A	119.7
C31—N3—C25	116.31 (13)	C30—C29—H29A	119.7
C31—N3—Y1	127.01 (11)	C29—C30—C25	120.59 (17)
C25—N3—Y1	116.62 (10)	C29—C30—H30A	119.7
N1—C1—C2	119.73 (15)	C25—C30—H30A	119.7
N1—C1—C6	123.53 (16)	N3—C31—C36	124.10 (15)
C2—C1—C6	116.70 (16)	N3—C31—C32	119.68 (15)
C3—C2—C1	121.34 (17)	C36—C31—C32	116.22 (15)
C3—C2—H2A	119.3	C33—C32—C31	121.81 (16)
C1—C2—H2A	119.3	C33—C32—H32A	119.1
C4—C3—C2	120.98 (18)	C31—C32—H32A	119.1
C4—C3—H3A	119.5	C32—C33—C34	120.68 (17)
C2—C3—H3A	119.5	C32—C33—H33A	119.7
C5—C4—C3	118.57 (17)	C34—C33—H33A	119.7
C5—C4—H4A	120.7	C35—C34—C33	118.52 (16)
C3—C4—H4A	120.7	C35—C34—H34A	120.7
C4—C5—C6	121.55 (18)	C33—C34—H34A	120.7
C4—C5—H5A	119.2	C34—C35—C36	121.63 (17)
C6—C5—H5A	119.2	C34—C35—H35A	119.2
C5—C6—C1	120.81 (18)	C36—C35—H35A	119.2
C5—C6—H6A	119.6	C35—C36—C31	121.13 (16)
C1—C6—H6A	119.6	C35—C36—H36A	119.4
C12—C7—C8	118.20 (16)	C31—C36—H36A	119.4
C12—C7—N1	121.20 (15)	O1—C37—C38	104.91 (13)
C8—C7—N1	120.50 (15)	O1—C37—H37A	110.8
C9—C8—C7	121.21 (17)	C38—C37—H37A	110.8
C9—C8—H8A	119.4	O1—C37—H37B	110.8
C7—C8—H8A	119.4	C38—C37—H37B	110.8
C8—C9—C10	120.01 (18)	H37A—C37—H37B	108.8
C8—C9—H9A	120.0	C37—C38—C39	101.81 (14)
C10—C9—H9A	120.0	C37—C38—H38A	111.4
C11—C10—C9	119.59 (18)	C39—C38—H38A	111.4
C11—C10—H10A	120.2	C37—C38—H38B	111.4
C9—C10—H10A	120.2	C39—C38—H38B	111.4
C10—C11—C12	120.23 (18)	H38A—C38—H38B	109.3
C10—C11—H11A	119.9	C40—C39—C38	101.86 (14)
C12—C11—H11A	119.9	C40—C39—H39A	111.4
C7—C12—C11	120.75 (17)	C38—C39—H39A	111.4
C7—C12—H12A	119.6	C40—C39—H39B	111.4
C11—C12—H12A	119.6	C38—C39—H39B	111.4
C14—C13—C18	117.60 (16)	H39A—C39—H39B	109.3
C14—C13—N2	119.69 (15)	O1—C40—C39	105.26 (13)
C18—C13—N2	122.58 (16)	O1—C40—H40A	110.7
C15—C14—C13	120.84 (17)	C39—C40—H40A	110.7
C15—C14—H14A	119.6	O1—C40—H40B	110.7
C13—C14—H14A	119.6	C39—C40—H40B	110.7
C16—C15—C14	120.81 (19)	H40A—C40—H40B	108.8
C16—C15—H15A	119.6	O2—C41—C42	105.39 (16)
C14—C15—H15A	119.6	O2—C41—H41A	110.7
C15—C16—C17	119.20 (18)	C42—C41—H41A	110.7
C15—C16—H16A	120.4	O2—C41—H41B	110.7
C17—C16—H16A	120.4	C42—C41—H41B	110.7
C16—C17—C18	120.52 (18)	H41A—C41—H41B	108.8
C16—C17—H17A	119.7	C43—C42—C41	105.81 (19)
C18—C17—H17A	119.7	C43—C42—H42A	110.6
C17—C18—C13	120.93 (18)	C41—C42—H42A	110.6
C17—C18—H18A	119.5	C43—C42—H42B	110.6
C13—C18—H18A	119.5	C41—C42—H42B	110.6
C24—C19—N2	119.45 (15)	H42A—C42—H42B	108.7
C24—C19—C20	117.43 (16)	C44—C43—C42	106.26 (19)
N2—C19—C20	122.85 (15)	C44—C43—H43A	110.5
C24—C19—Y1	85.35 (10)	C42—C43—H43A	110.5
N2—C19—Y1	46.94 (7)	C44—C43—H43B	110.5
C20—C19—Y1	136.08 (11)	C42—C43—H43B	110.5
C21—C20—C19	121.10 (17)	H43A—C43—H43B	108.7
C21—C20—H20A	119.4	O2—C44—C43	106.06 (16)
C19—C20—H20A	119.4	O2—C44—H44A	110.5
C20—C21—C22	120.87 (17)	C43—C44—H44A	110.5
C20—C21—H21A	119.6	O2—C44—H44B	110.5
C22—C21—H21A	119.6	C43—C44—H44B	110.5
C23—C22—C21	119.05 (17)	H44A—C44—H44B	108.7
			
C7—N1—C1—C2	−166.06 (15)	C20—C21—C22—C23	0.8 (3)
Y1—N1—C1—C2	15.1 (2)	C21—C22—C23—C24	−0.4 (3)
C7—N1—C1—C6	16.3 (2)	C22—C23—C24—C19	−0.8 (3)
Y1—N1—C1—C6	−162.48 (13)	N2—C19—C24—C23	−172.72 (15)
N1—C1—C2—C3	−175.27 (16)	C20—C19—C24—C23	1.4 (2)
C6—C1—C2—C3	2.5 (2)	Y1—C19—C24—C23	−139.09 (15)
C1—C2—C3—C4	−1.4 (3)	C31—N3—C25—C30	−95.94 (19)
C2—C3—C4—C5	−0.7 (3)	Y1—N3—C25—C30	86.57 (17)
C3—C4—C5—C6	1.7 (3)	C31—N3—C25—C26	88.07 (19)
C4—C5—C6—C1	−0.5 (3)	Y1—N3—C25—C26	−89.42 (16)
N1—C1—C6—C5	176.11 (16)	C30—C25—C26—C27	−1.1 (2)
C2—C1—C6—C5	−1.6 (2)	N3—C25—C26—C27	175.01 (15)
C1—N1—C7—C12	−111.78 (18)	C25—C26—C27—C28	0.4 (3)
Y1—N1—C7—C12	67.13 (18)	C26—C27—C28—C29	0.4 (3)
C1—N1—C7—C8	71.9 (2)	C27—C28—C29—C30	−0.5 (3)
Y1—N1—C7—C8	−109.15 (15)	C28—C29—C30—C25	−0.2 (3)
C12—C7—C8—C9	0.6 (3)	C26—C25—C30—C29	1.0 (3)
N1—C7—C8—C9	177.01 (16)	N3—C25—C30—C29	−175.06 (16)
C7—C8—C9—C10	−0.9 (3)	C25—N3—C31—C36	9.5 (2)
C8—C9—C10—C11	0.5 (3)	Y1—N3—C31—C36	−173.32 (12)
C9—C10—C11—C12	0.2 (3)	C25—N3—C31—C32	−170.81 (15)
C8—C7—C12—C11	0.1 (3)	Y1—N3—C31—C32	6.4 (2)
N1—C7—C12—C11	−176.30 (16)	N3—C31—C32—C33	178.94 (16)
C10—C11—C12—C7	−0.5 (3)	C36—C31—C32—C33	−1.3 (2)
C19—N2—C13—C14	−150.24 (16)	C31—C32—C33—C34	0.8 (3)
Y1—N2—C13—C14	9.6 (2)	C32—C33—C34—C35	0.4 (3)
C19—N2—C13—C18	34.0 (2)	C33—C34—C35—C36	−1.1 (3)
Y1—N2—C13—C18	−166.16 (13)	C34—C35—C36—C31	0.5 (3)
C18—C13—C14—C15	3.1 (3)	N3—C31—C36—C35	−179.59 (16)
N2—C13—C14—C15	−172.83 (17)	C32—C31—C36—C35	0.7 (3)
C13—C14—C15—C16	−0.7 (3)	C40—O1—C37—C38	−15.01 (18)
C14—C15—C16—C17	−1.6 (3)	Y1—O1—C37—C38	158.85 (11)
C15—C16—C17—C18	1.5 (3)	O1—C37—C38—C39	34.95 (18)
C16—C17—C18—C13	1.0 (3)	C37—C38—C39—C40	−41.13 (17)
C14—C13—C18—C17	−3.3 (3)	C37—O1—C40—C39	−11.27 (17)
N2—C13—C18—C17	172.56 (17)	Y1—O1—C40—C39	173.99 (10)
C13—N2—C19—C24	−146.07 (15)	C38—C39—C40—O1	32.67 (17)
Y1—N2—C19—C24	49.07 (17)	C44—O2—C41—C42	−30.5 (2)
C13—N2—C19—C20	40.1 (2)	Y1—O2—C41—C42	147.90 (17)
Y1—N2—C19—C20	−124.76 (14)	O2—C41—C42—C43	18.0 (3)
C13—N2—C19—Y1	164.86 (18)	C41—C42—C43—C44	0.8 (3)
C24—C19—C20—C21	−1.0 (2)	C41—O2—C44—C43	31.4 (2)
N2—C19—C20—C21	172.96 (16)	Y1—O2—C44—C43	−147.18 (16)
Y1—C19—C20—C21	113.04 (19)	C42—C43—C44—O2	−19.3 (3)
C19—C20—C21—C22	−0.1 (3)		

### Tris(diphenylamido-κN)bis(tetrahydrofuran-κO)erbium(III) (1-Er) Crystal data

**Table:**

[Er(C_12_H_10_N)_3_(C_4_H_8_O)_2_]	*F*(000) = 1660
*M**_r_* = 816.10	*D*_x_ = 1.434 Mg m^−^^3^
Monoclinic, *P*2_1_/*n*	Mo *K*α radiation, λ = 0.71073 Å
*a* = 12.0946 (5) Å	Cell parameters from 9998 reflections
*b* = 19.1086 (8) Å	θ = 2.5–29.0°
*c* = 16.3609 (7) Å	µ = 2.26 mm^−^^1^
β = 91.3697 (5)°	*T* = 173 K
*V* = 3780.1 (3) Å^3^	Irregular, pink
*Z* = 4	0.48 × 0.39 × 0.33 mm

### Tris(diphenylamido-κN)bis(tetrahydrofuran-κO)erbium(III) (1-Er) Data collection

**Table:**

Bruker SMART APEXII CCD diffractometer	8834 reflections with *I* > 2σ(*I*)
Radiation source: fine-focus sealed tube	*R*_int_ = 0.018
φ and ω scans	θ_max_ = 29.2°, θ_min_ = 1.6°
Absorption correction: multi-scan (SADABS; Krause et al., 2015)	*h* = −16→16
*T*_min_ = 0.557, *T*_max_ = 0.695	*k* = −25→24
46529 measured reflections	*l* = −22→22
9687 independent reflections	

### Tris(diphenylamido-κN)bis(tetrahydrofuran-κO)erbium(III) (1-Er) Refinement

**Table:**

Refinement on *F*^2^	Primary atom site location: structure-invariant direct methods
Least-squares matrix: full	Secondary atom site location: difference Fourier map
*R*[*F*^2^ > 2σ(*F*^2^)] = 0.019	Hydrogen site location: inferred from neighbouring sites
*wR*(*F*^2^) = 0.050	H-atom parameters constrained
*S* = 1.04	*w* = 1/[σ^2^(*F*_o_^2^) + (0.0257*P*)^2^ + 1.7012*P*] where *P* = (*F*_o_^2^ + 2*F*_c_^2^)/3
9687 reflections	(Δ/σ)_max_ = 0.004
462 parameters	Δρ_max_ = 0.70 e Å^−^^3^
28 restraints	Δρ_min_ = −0.42 e Å^−^^3^

### Tris(diphenylamido-κN)bis(tetrahydrofuran-κO)erbium(III) (1-Er) Special details

**Table:**

Geometry. All esds (except the esd in the dihedral angle between two l.s. planes) are estimated using the full covariance matrix. The cell esds are taken into account individually in the estimation of esds in distances, angles and torsion angles; correlations between esds in cell parameters are only used when they are defined by crystal symmetry. An approximate (isotropic) treatment of cell esds is used for estimating esds involving l.s. planes.
Refinement. A pink crystal of approximate dimensions 0.332 x 0.389 x 0.482 mm was mounted on a glass fiber and transferred to a Bruker SMART APEX II diffractometer. The APEX2 program package was used to determine the unit-cell parameters and for data collection (15 sec/frame scan time for a sphere of diffraction data). The raw frame data was processed using SAINT and SADABS to yield the reflection data file. Subsequent calculations were carried out using the SHELXTL program. The diffraction symmetry was 2/m and the systematic absences were consistent with the monoclinic space group P21/n that was later determined to be correct.The structure was solved by direct methods and refined on F2 by full-matrix least-squares techniques. The analytical scattering factors for neutral atoms were used throughout the analysis. Hydrogen atoms were included using a riding model. The tetrahydrofuran ligands were disordered and were included using multiple components with partial site-occupancy-factors.Least-squares analysis yielded wR2 = 0.0504 and Goof = 1.041 for 462 variables refined against 9687 data (0.73 Å), R1 = 0.0194 for those 8834 data with I > 2.0sigma(I).

### Tris(diphenylamido-κN)bis(tetrahydrofuran-κO)erbium(III) (1-Er) Fractional atomic coordinates and isotropic or equivalent isotropic displacement parameters (Å^2^)

**Table:**

	*x*	*y*	*z*	*U*_iso_*/*U*_eq_	Occ. (<1)
Er1	0.46777 (2)	0.72342 (2)	0.36679 (2)	0.02729 (3)	
O1	0.41457 (11)	0.78132 (6)	0.48614 (8)	0.0377 (3)	
O2	0.49661 (10)	0.64664 (6)	0.25832 (7)	0.0342 (3)	
N1	0.28353 (12)	0.71121 (8)	0.33981 (9)	0.0327 (3)	
N2	0.58473 (13)	0.65462 (8)	0.43975 (9)	0.0369 (3)	
N3	0.55625 (12)	0.81628 (8)	0.31723 (10)	0.0382 (3)	
C1	0.24325 (13)	0.64570 (9)	0.30972 (11)	0.0316 (3)	
C2	0.23877 (18)	0.58668 (11)	0.35926 (13)	0.0454 (5)	
H2	0.2603	0.5902	0.4153	0.054*	
C3	0.20326 (19)	0.52253 (11)	0.32804 (15)	0.0533 (5)	
H3	0.2010	0.4829	0.3629	0.064*	
C4	0.17145 (18)	0.51609 (11)	0.24715 (14)	0.0496 (5)	
H4	0.1481	0.4721	0.2258	0.060*	
C5	0.17397 (19)	0.57433 (12)	0.19752 (13)	0.0503 (5)	
H5	0.1515	0.5705	0.1417	0.060*	
C6	0.20901 (16)	0.63846 (10)	0.22831 (11)	0.0401 (4)	
H6	0.2097	0.6781	0.1933	0.048*	
C7	0.20194 (15)	0.76085 (9)	0.35533 (10)	0.0325 (3)	
C8	0.09037 (16)	0.74366 (11)	0.36552 (12)	0.0388 (4)	
H8	0.0669	0.6966	0.3583	0.047*	
C9	0.01340 (19)	0.79475 (13)	0.38613 (14)	0.0505 (5)	
H9	−0.0616	0.7819	0.3932	0.061*	
C10	0.0446 (2)	0.86363 (13)	0.39638 (13)	0.0541 (6)	
H10	−0.0081	0.8981	0.4106	0.065*	
C11	0.1541 (2)	0.88162 (11)	0.38556 (12)	0.0496 (5)	
H11	0.1764	0.9290	0.3923	0.059*	
C12	0.23129 (17)	0.83184 (10)	0.36517 (11)	0.0401 (4)	
H12	0.3058	0.8457	0.3576	0.048*	
C13	0.68712 (15)	0.62707 (11)	0.41862 (11)	0.0396 (4)	
C14	0.73872 (17)	0.57185 (11)	0.46194 (14)	0.0473 (5)	
H14	0.7053	0.5538	0.5095	0.057*	
C15	0.8377 (2)	0.54336 (13)	0.43620 (18)	0.0636 (7)	
H15	0.8710	0.5062	0.4664	0.076*	
C16	0.8879 (2)	0.56814 (17)	0.36780 (18)	0.0743 (9)	
H16	0.9547	0.5476	0.3500	0.089*	
C17	0.8405 (2)	0.62298 (18)	0.32529 (15)	0.0707 (8)	
H17	0.8758	0.6408	0.2784	0.085*	
C18	0.74103 (17)	0.65291 (14)	0.35007 (13)	0.0538 (6)	
H18	0.7099	0.6910	0.3202	0.065*	
C19	0.54807 (15)	0.63758 (9)	0.51950 (10)	0.0341 (4)	
C20	0.46354 (16)	0.58936 (10)	0.53102 (12)	0.0398 (4)	
H20	0.4344	0.5639	0.4855	0.048*	
C21	0.42101 (19)	0.57793 (11)	0.60840 (13)	0.0475 (5)	
H21	0.3624	0.5455	0.6150	0.057*	
C22	0.4635 (2)	0.61339 (12)	0.67505 (13)	0.0526 (5)	
H22	0.4338	0.6059	0.7276	0.063*	
C23	0.5498 (2)	0.65999 (12)	0.66552 (12)	0.0507 (5)	
H23	0.5806	0.6836	0.7118	0.061*	
C24	0.59134 (16)	0.67236 (11)	0.58859 (11)	0.0415 (4)	
H24	0.6500	0.7049	0.5826	0.050*	
C25	0.63270 (15)	0.85741 (11)	0.36236 (12)	0.0416 (4)	
C26	0.69543 (17)	0.82757 (13)	0.42630 (13)	0.0482 (5)	
H26	0.6919	0.7785	0.4353	0.058*	
C27	0.7629 (2)	0.86864 (16)	0.47698 (16)	0.0645 (7)	
H27	0.8037	0.8472	0.5205	0.077*	
C28	0.7714 (2)	0.93895 (17)	0.4653 (2)	0.0823 (10)	
H28	0.8172	0.9668	0.5003	0.099*	
C29	0.7123 (3)	0.96868 (15)	0.4017 (2)	0.0867 (10)	
H29	0.7185	1.0176	0.3924	0.104*	
C30	0.6437 (2)	0.92913 (13)	0.35027 (18)	0.0632 (7)	
H30	0.6040	0.9512	0.3066	0.076*	
C31	0.54035 (15)	0.83254 (9)	0.23354 (11)	0.0350 (4)	
C32	0.62980 (18)	0.84668 (11)	0.18308 (13)	0.0462 (5)	
H32	0.7025	0.8494	0.2061	0.055*	
C33	0.6130 (2)	0.85670 (13)	0.10019 (15)	0.0608 (7)	
H33	0.6743	0.8668	0.0670	0.073*	
C34	0.5090 (2)	0.85236 (13)	0.06487 (14)	0.0627 (7)	
H34	0.4985	0.8586	0.0076	0.075*	
C35	0.4203 (2)	0.83897 (12)	0.11336 (14)	0.0528 (5)	
H35	0.3482	0.8360	0.0893	0.063*	
C36	0.43466 (16)	0.82969 (10)	0.19732 (12)	0.0406 (4)	
H36	0.3723	0.8214	0.2302	0.049*	
C37	0.32888 (16)	0.75422 (11)	0.53980 (11)	0.0399 (4)	
H37A	0.3413	0.7040	0.5519	0.048*	0.373 (12)
H37B	0.2544	0.7600	0.5142	0.048*	0.373 (12)
H37C	0.3558	0.7122	0.5695	0.048*	0.627 (12)
H37D	0.2615	0.7416	0.5075	0.048*	0.627 (12)
C38	0.3404 (9)	0.7980 (6)	0.6171 (5)	0.0594 (9)	0.373 (12)
H38A	0.2728	0.8262	0.6249	0.071*	0.373 (12)
H38B	0.3515	0.7673	0.6654	0.071*	0.373 (12)
C39	0.4396 (9)	0.8454 (7)	0.6074 (5)	0.0594 (9)	0.373 (12)
H39A	0.4255	0.8925	0.6302	0.071*	0.373 (12)
H39B	0.5063	0.8254	0.6347	0.071*	0.373 (12)
C38B	0.3045 (6)	0.8127 (3)	0.5990 (4)	0.0594 (9)	0.627 (12)
H38C	0.2464	0.8443	0.5767	0.071*	0.627 (12)
H38D	0.2810	0.7940	0.6523	0.071*	0.627 (12)
C39B	0.4123 (6)	0.8488 (4)	0.6068 (3)	0.0594 (9)	0.627 (12)
H39C	0.4640	0.8231	0.6437	0.071*	0.627 (12)
H39D	0.4035	0.8972	0.6273	0.071*	0.627 (12)
C40	0.4521 (2)	0.84841 (14)	0.51903 (15)	0.0667 (7)	
H40A	0.5305	0.8565	0.5057	0.080*	0.373 (12)
H40B	0.4070	0.8870	0.4954	0.080*	0.373 (12)
H40C	0.4191	0.8877	0.4874	0.080*	0.627 (12)
H40D	0.5337	0.8521	0.5178	0.080*	0.627 (12)
C41	0.4980 (2)	0.66225 (11)	0.17130 (12)	0.0475 (5)	
H41A	0.5565	0.6968	0.1595	0.057*	
H41B	0.4258	0.6814	0.1523	0.057*	
C42	0.5211 (2)	0.59331 (14)	0.12968 (13)	0.0617 (7)	
H42A	0.5757	0.5994	0.0861	0.074*	0.633 (7)
H42B	0.4524	0.5732	0.1054	0.074*	0.633 (7)
H42C	0.4789	0.5896	0.0773	0.074*	0.367 (7)
H42D	0.6009	0.5885	0.1188	0.074*	0.367 (7)
C43	0.5677 (4)	0.54620 (19)	0.1983 (2)	0.0532 (12)	0.633 (7)
H43A	0.5525	0.4962	0.1868	0.064*	0.633 (7)
H43B	0.6483	0.5530	0.2064	0.064*	0.633 (7)
C44	0.4865 (6)	0.5413 (3)	0.1856 (4)	0.0460 (18)	0.367 (7)
H44A	0.5295	0.4977	0.1786	0.055*	0.367 (7)
H44B	0.4070	0.5306	0.1767	0.055*	0.367 (7)
C45	0.50666 (19)	0.57083 (10)	0.26924 (12)	0.0441 (4)	
H45A	0.4327	0.5487	0.2707	0.053*	0.633 (7)
H45B	0.5477	0.5598	0.3207	0.053*	0.633 (7)
H45C	0.4511	0.5533	0.3077	0.053*	0.367 (7)
H45D	0.5814	0.5582	0.2904	0.053*	0.367 (7)

### Tris(diphenylamido-κN)bis(tetrahydrofuran-κO)erbium(III) (1-Er) Atomic displacement parameters (Å^2^)

**Table:**

	*U*^11^	*U*^22^	*U*^33^	*U*^12^	*U*^13^	*U*^23^
Er1	0.02622 (4)	0.02886 (4)	0.02674 (4)	−0.00102 (3)	−0.00015 (3)	−0.00231 (3)
O1	0.0382 (7)	0.0401 (7)	0.0350 (7)	−0.0038 (5)	0.0066 (5)	−0.0073 (5)
O2	0.0407 (7)	0.0316 (6)	0.0301 (6)	0.0045 (5)	−0.0021 (5)	−0.0037 (5)
N1	0.0295 (7)	0.0319 (7)	0.0367 (8)	−0.0009 (6)	−0.0021 (6)	−0.0008 (6)
N2	0.0357 (8)	0.0464 (9)	0.0285 (7)	0.0050 (6)	−0.0036 (6)	−0.0013 (6)
N3	0.0328 (7)	0.0422 (9)	0.0394 (8)	−0.0051 (6)	−0.0015 (6)	0.0072 (7)
C1	0.0250 (7)	0.0334 (8)	0.0362 (8)	−0.0011 (6)	−0.0011 (6)	0.0017 (7)
C2	0.0500 (11)	0.0440 (11)	0.0418 (10)	−0.0053 (9)	−0.0082 (8)	0.0104 (8)
C3	0.0587 (13)	0.0350 (10)	0.0659 (14)	−0.0065 (9)	−0.0044 (11)	0.0123 (9)
C4	0.0469 (11)	0.0359 (10)	0.0662 (14)	−0.0064 (8)	0.0029 (10)	−0.0103 (9)
C5	0.0549 (12)	0.0534 (12)	0.0425 (10)	−0.0082 (10)	−0.0047 (9)	−0.0092 (9)
C6	0.0443 (10)	0.0398 (10)	0.0358 (9)	−0.0042 (8)	−0.0045 (8)	0.0035 (7)
C7	0.0345 (9)	0.0367 (9)	0.0264 (8)	0.0025 (7)	−0.0009 (6)	0.0046 (6)
C8	0.0348 (9)	0.0443 (10)	0.0374 (9)	0.0033 (8)	0.0039 (7)	0.0067 (8)
C9	0.0431 (11)	0.0627 (13)	0.0464 (11)	0.0127 (10)	0.0121 (9)	0.0114 (10)
C10	0.0650 (14)	0.0575 (13)	0.0405 (11)	0.0269 (11)	0.0134 (10)	0.0082 (9)
C11	0.0699 (14)	0.0387 (10)	0.0403 (10)	0.0111 (10)	0.0043 (10)	0.0035 (8)
C12	0.0454 (10)	0.0373 (9)	0.0377 (9)	0.0016 (8)	0.0003 (8)	0.0044 (8)
C13	0.0338 (9)	0.0497 (11)	0.0347 (9)	0.0011 (8)	−0.0077 (7)	−0.0150 (8)
C14	0.0397 (10)	0.0408 (10)	0.0609 (13)	0.0020 (8)	−0.0114 (9)	−0.0137 (9)
C15	0.0453 (12)	0.0548 (14)	0.0901 (19)	0.0108 (10)	−0.0134 (12)	−0.0271 (13)
C16	0.0393 (12)	0.103 (2)	0.0801 (18)	0.0149 (13)	−0.0079 (12)	−0.0497 (17)
C17	0.0423 (12)	0.125 (3)	0.0446 (12)	−0.0021 (14)	0.0014 (10)	−0.0286 (14)
C18	0.0394 (10)	0.0857 (17)	0.0360 (10)	0.0026 (11)	−0.0037 (8)	−0.0104 (10)
C19	0.0356 (9)	0.0353 (9)	0.0309 (8)	0.0057 (7)	−0.0072 (7)	0.0001 (7)
C20	0.0461 (10)	0.0345 (9)	0.0382 (9)	0.0004 (8)	−0.0106 (8)	−0.0009 (7)
C21	0.0547 (12)	0.0418 (10)	0.0456 (11)	−0.0086 (9)	−0.0053 (9)	0.0122 (9)
C22	0.0676 (14)	0.0561 (13)	0.0339 (10)	−0.0034 (11)	−0.0033 (9)	0.0129 (9)
C23	0.0630 (13)	0.0591 (13)	0.0293 (9)	−0.0054 (10)	−0.0123 (9)	−0.0014 (9)
C24	0.0430 (10)	0.0463 (10)	0.0348 (9)	−0.0060 (8)	−0.0082 (7)	−0.0006 (8)
C25	0.0308 (9)	0.0470 (11)	0.0470 (10)	−0.0017 (8)	0.0044 (8)	−0.0029 (8)
C26	0.0381 (10)	0.0634 (13)	0.0430 (10)	−0.0030 (9)	0.0003 (8)	−0.0025 (10)
C27	0.0450 (12)	0.093 (2)	0.0548 (13)	0.0044 (12)	−0.0061 (10)	−0.0214 (13)
C28	0.0579 (15)	0.078 (2)	0.110 (2)	0.0052 (14)	−0.0187 (16)	−0.0500 (18)
C29	0.0721 (19)	0.0491 (15)	0.138 (3)	−0.0003 (13)	−0.0213 (19)	−0.0267 (17)
C30	0.0524 (13)	0.0446 (12)	0.0916 (19)	0.0017 (10)	−0.0148 (13)	−0.0063 (12)
C31	0.0378 (9)	0.0261 (8)	0.0412 (9)	0.0024 (7)	0.0025 (7)	0.0058 (7)
C32	0.0426 (10)	0.0432 (11)	0.0532 (12)	0.0085 (8)	0.0112 (9)	0.0118 (9)
C33	0.0713 (16)	0.0592 (14)	0.0531 (13)	0.0253 (12)	0.0263 (12)	0.0169 (11)
C34	0.0902 (19)	0.0606 (14)	0.0376 (11)	0.0326 (13)	0.0059 (11)	0.0067 (10)
C35	0.0610 (13)	0.0484 (12)	0.0483 (12)	0.0173 (10)	−0.0119 (10)	0.0027 (9)
C36	0.0396 (10)	0.0369 (9)	0.0452 (10)	0.0051 (8)	−0.0004 (8)	0.0058 (8)
C37	0.0408 (10)	0.0448 (10)	0.0345 (9)	−0.0017 (8)	0.0079 (7)	0.0003 (8)
C38	0.051 (3)	0.0699 (15)	0.0579 (12)	−0.0018 (16)	0.0153 (13)	−0.0210 (11)
C39	0.051 (3)	0.0699 (15)	0.0579 (12)	−0.0018 (16)	0.0153 (13)	−0.0210 (11)
C38B	0.051 (3)	0.0699 (15)	0.0579 (12)	−0.0018 (16)	0.0153 (13)	−0.0210 (11)
C39B	0.051 (3)	0.0699 (15)	0.0579 (12)	−0.0018 (16)	0.0153 (13)	−0.0210 (11)
C40	0.0775 (17)	0.0637 (15)	0.0602 (14)	−0.0287 (13)	0.0271 (12)	−0.0318 (12)
C41	0.0675 (14)	0.0425 (11)	0.0326 (9)	0.0046 (10)	0.0028 (9)	−0.0012 (8)
C42	0.094 (2)	0.0530 (14)	0.0379 (11)	0.0079 (12)	0.0039 (12)	−0.0103 (9)
C43	0.067 (3)	0.0430 (18)	0.050 (2)	0.0125 (17)	0.0045 (18)	−0.0118 (15)
C44	0.053 (4)	0.035 (3)	0.049 (3)	0.004 (2)	−0.013 (3)	−0.013 (2)
C45	0.0623 (13)	0.0312 (9)	0.0384 (10)	0.0047 (8)	−0.0038 (9)	−0.0036 (7)

### Tris(diphenylamido-κN)bis(tetrahydrofuran-κO)erbium(III) (1-Er) Geometric parameters (Å, º)

**Table:**

Er1—N3	2.2344 (15)	C26—C27	1.392 (3)
Er1—N2	2.2524 (15)	C26—H26	0.9500
Er1—N1	2.2733 (15)	C27—C28	1.361 (4)
Er1—O2	2.3353 (11)	C27—H27	0.9500
Er1—O1	2.3475 (12)	C28—C29	1.371 (5)
O1—C40	1.458 (2)	C28—H28	0.9500
O1—C37	1.469 (2)	C29—C30	1.391 (4)
O2—C41	1.455 (2)	C29—H29	0.9500
O2—C45	1.464 (2)	C30—H30	0.9500
N1—C7	1.396 (2)	C31—C36	1.397 (3)
N1—C1	1.427 (2)	C31—C32	1.403 (3)
N2—C13	1.397 (2)	C32—C33	1.380 (3)
N2—C19	1.426 (2)	C32—H32	0.9500
N3—C25	1.409 (2)	C33—C34	1.374 (4)
N3—C31	1.413 (2)	C33—H33	0.9500
C1—C2	1.391 (3)	C34—C35	1.373 (4)
C1—C6	1.392 (2)	C34—H34	0.9500
C2—C3	1.392 (3)	C35—C36	1.392 (3)
C2—H2	0.9500	C35—H35	0.9500
C3—C4	1.375 (3)	C36—H36	0.9500
C3—H3	0.9500	C37—C38B	1.512 (4)
C4—C5	1.378 (3)	C37—C38	1.520 (7)
C4—H4	0.9500	C37—H37A	0.9900
C5—C6	1.387 (3)	C37—H37B	0.9900
C5—H5	0.9500	C37—H37C	0.9900
C6—H6	0.9500	C37—H37D	0.9900
C7—C8	1.403 (3)	C38—C39	1.515 (8)
C7—C12	1.410 (3)	C38—H38A	0.9900
C8—C9	1.396 (3)	C38—H38B	0.9900
C8—H8	0.9500	C39—C40	1.458 (8)
C9—C10	1.379 (4)	C39—H39A	0.9900
C9—H9	0.9500	C39—H39B	0.9900
C10—C11	1.383 (3)	C38B—C39B	1.479 (5)
C10—H10	0.9500	C38B—H38C	0.9900
C11—C12	1.380 (3)	C38B—H38D	0.9900
C11—H11	0.9500	C39B—C40	1.525 (5)
C12—H12	0.9500	C39B—H39C	0.9900
C13—C18	1.401 (3)	C39B—H39D	0.9900
C13—C14	1.408 (3)	C40—H40A	0.9900
C14—C15	1.389 (3)	C40—H40B	0.9900
C14—H14	0.9500	C40—H40C	0.9900
C15—C16	1.370 (4)	C40—H40D	0.9900
C15—H15	0.9500	C41—C42	1.512 (3)
C16—C17	1.375 (4)	C41—H41A	0.9900
C16—H16	0.9500	C41—H41B	0.9900
C17—C18	1.401 (3)	C42—C44	1.421 (7)
C17—H17	0.9500	C42—C43	1.535 (5)
C18—H18	0.9500	C42—H42A	0.9900
C19—C20	1.392 (3)	C42—H42B	0.9900
C19—C24	1.402 (2)	C42—H42C	0.9900
C20—C21	1.395 (3)	C42—H42D	0.9900
C20—H20	0.9500	C43—C45	1.468 (4)
C21—C22	1.373 (3)	C43—H43A	0.9900
C21—H21	0.9500	C43—H43B	0.9900
C22—C23	1.383 (3)	C44—C45	1.495 (6)
C22—H22	0.9500	C44—H44A	0.9900
C23—C24	1.387 (3)	C44—H44B	0.9900
C23—H23	0.9500	C45—H45A	0.9900
C24—H24	0.9500	C45—H45B	0.9900
C25—C30	1.391 (3)	C45—H45C	0.9900
C25—C26	1.399 (3)	C45—H45D	0.9900
			
N3—Er1—N2	110.83 (6)	C29—C30—C25	120.5 (3)
N3—Er1—N1	119.13 (5)	C29—C30—H30	119.8
N2—Er1—N1	130.04 (6)	C25—C30—H30	119.8
N3—Er1—O2	98.14 (5)	C36—C31—C32	117.94 (18)
N2—Er1—O2	86.14 (5)	C36—C31—N3	120.30 (16)
N1—Er1—O2	87.22 (5)	C32—C31—N3	121.59 (17)
N3—Er1—O1	94.06 (5)	C33—C32—C31	120.5 (2)
N2—Er1—O1	90.89 (5)	C33—C32—H32	119.8
N1—Er1—O1	85.59 (5)	C31—C32—H32	119.8
O2—Er1—O1	167.72 (4)	C34—C33—C32	121.1 (2)
C40—O1—C37	107.88 (14)	C34—C33—H33	119.4
C40—O1—Er1	129.21 (12)	C32—C33—H33	119.4
C37—O1—Er1	122.85 (10)	C35—C34—C33	119.2 (2)
C41—O2—C45	108.63 (13)	C35—C34—H34	120.4
C41—O2—Er1	128.32 (11)	C33—C34—H34	120.4
C45—O2—Er1	122.84 (10)	C34—C35—C36	120.9 (2)
C7—N1—C1	114.95 (14)	C34—C35—H35	119.6
C7—N1—Er1	126.06 (11)	C36—C35—H35	119.6
C1—N1—Er1	118.82 (11)	C35—C36—C31	120.33 (19)
C13—N2—C19	115.94 (15)	C35—C36—H36	119.8
C13—N2—Er1	129.66 (12)	C31—C36—H36	119.8
C19—N2—Er1	114.40 (11)	O1—C37—C38B	105.9 (2)
C25—N3—C31	117.24 (15)	O1—C37—C38	104.6 (3)
C25—N3—Er1	124.53 (12)	O1—C37—H37A	110.8
C31—N3—Er1	118.10 (11)	C38—C37—H37A	110.8
C2—C1—C6	117.53 (17)	O1—C37—H37B	110.8
C2—C1—N1	121.89 (16)	C38—C37—H37B	110.8
C6—C1—N1	120.57 (16)	H37A—C37—H37B	108.9
C1—C2—C3	121.08 (19)	O1—C37—H37C	110.6
C1—C2—H2	119.5	C38B—C37—H37C	110.6
C3—C2—H2	119.5	O1—C37—H37D	110.6
C4—C3—C2	120.58 (19)	C38B—C37—H37D	110.6
C4—C3—H3	119.7	H37C—C37—H37D	108.7
C2—C3—H3	119.7	C39—C38—C37	107.4 (5)
C3—C4—C5	119.03 (19)	C39—C38—H38A	110.2
C3—C4—H4	120.5	C37—C38—H38A	110.2
C5—C4—H4	120.5	C39—C38—H38B	110.2
C4—C5—C6	120.66 (19)	C37—C38—H38B	110.2
C4—C5—H5	119.7	H38A—C38—H38B	108.5
C6—C5—H5	119.7	C40—C39—C38	103.3 (6)
C5—C6—C1	121.10 (18)	C40—C39—H39A	111.1
C5—C6—H6	119.5	C38—C39—H39A	111.1
C1—C6—H6	119.5	C40—C39—H39B	111.1
N1—C7—C8	123.27 (17)	C38—C39—H39B	111.1
N1—C7—C12	119.81 (16)	H39A—C39—H39B	109.1
C8—C7—C12	116.86 (17)	C39B—C38B—C37	102.4 (3)
C9—C8—C7	120.9 (2)	C39B—C38B—H38C	111.3
C9—C8—H8	119.5	C37—C38B—H38C	111.3
C7—C8—H8	119.5	C39B—C38B—H38D	111.3
C10—C9—C8	121.0 (2)	C37—C38B—H38D	111.3
C10—C9—H9	119.5	H38C—C38B—H38D	109.2
C8—C9—H9	119.5	C38B—C39B—C40	102.4 (4)
C9—C10—C11	118.8 (2)	C38B—C39B—H39C	111.3
C9—C10—H10	120.6	C40—C39B—H39C	111.3
C11—C10—H10	120.6	C38B—C39B—H39D	111.3
C12—C11—C10	121.0 (2)	C40—C39B—H39D	111.3
C12—C11—H11	119.5	H39C—C39B—H39D	109.2
C10—C11—H11	119.5	C39—C40—O1	107.0 (5)
C11—C12—C7	121.4 (2)	O1—C40—C39B	104.5 (3)
C11—C12—H12	119.3	C39—C40—H40A	110.3
C7—C12—H12	119.3	O1—C40—H40A	110.3
N2—C13—C18	119.85 (19)	C39—C40—H40B	110.3
N2—C13—C14	122.87 (19)	O1—C40—H40B	110.3
C18—C13—C14	117.25 (19)	H40A—C40—H40B	108.6
C15—C14—C13	121.0 (2)	O1—C40—H40C	110.9
C15—C14—H14	119.5	C39B—C40—H40C	110.9
C13—C14—H14	119.5	O1—C40—H40D	110.9
C16—C15—C14	120.9 (3)	C39B—C40—H40D	110.9
C16—C15—H15	119.5	H40C—C40—H40D	108.9
C14—C15—H15	119.5	O2—C41—C42	105.57 (16)
C15—C16—C17	119.3 (2)	O2—C41—H41A	110.6
C15—C16—H16	120.4	C42—C41—H41A	110.6
C17—C16—H16	120.4	O2—C41—H41B	110.6
C16—C17—C18	121.0 (3)	C42—C41—H41B	110.6
C16—C17—H17	119.5	H41A—C41—H41B	108.8
C18—C17—H17	119.5	C44—C42—C41	105.0 (3)
C13—C18—C17	120.5 (2)	C41—C42—C43	104.49 (19)
C13—C18—H18	119.8	C41—C42—H42A	110.9
C17—C18—H18	119.8	C43—C42—H42A	110.9
C20—C19—C24	117.75 (17)	C41—C42—H42B	110.9
C20—C19—N2	121.36 (16)	C43—C42—H42B	110.9
C24—C19—N2	120.76 (17)	H42A—C42—H42B	108.9
C19—C20—C21	120.95 (17)	C44—C42—H42C	110.7
C19—C20—H20	119.5	C41—C42—H42C	110.7
C21—C20—H20	119.5	C44—C42—H42D	110.7
C22—C21—C20	120.3 (2)	C41—C42—H42D	110.7
C22—C21—H21	119.9	H42C—C42—H42D	108.8
C20—C21—H21	119.9	C45—C43—C42	102.0 (3)
C21—C22—C23	119.8 (2)	C45—C43—H43A	111.4
C21—C22—H22	120.1	C42—C43—H43A	111.4
C23—C22—H22	120.1	C45—C43—H43B	111.4
C22—C23—C24	120.18 (19)	C42—C43—H43B	111.4
C22—C23—H23	119.9	H43A—C43—H43B	109.2
C24—C23—H23	119.9	C42—C44—C45	106.3 (4)
C23—C24—C19	120.98 (19)	C42—C44—H44A	110.5
C23—C24—H24	119.5	C45—C44—H44A	110.5
C19—C24—H24	119.5	C42—C44—H44B	110.5
C30—C25—C26	117.1 (2)	C45—C44—H44B	110.5
C30—C25—N3	122.6 (2)	H44A—C44—H44B	108.7
C26—C25—N3	120.13 (19)	O2—C45—C43	105.2 (2)
C27—C26—C25	121.0 (2)	O2—C45—C44	104.5 (3)
C27—C26—H26	119.5	O2—C45—H45A	110.7
C25—C26—H26	119.5	C43—C45—H45A	110.7
C28—C27—C26	121.2 (3)	O2—C45—H45B	110.7
C28—C27—H27	119.4	C43—C45—H45B	110.7
C26—C27—H27	119.4	H45A—C45—H45B	108.8
C27—C28—C29	118.4 (3)	O2—C45—H45C	110.8
C27—C28—H28	120.8	C44—C45—H45C	110.8
C29—C28—H28	120.8	O2—C45—H45D	110.8
C28—C29—C30	121.8 (3)	C44—C45—H45D	110.8
C28—C29—H29	119.1	H45C—C45—H45D	108.9
C30—C29—H29	119.1		
			
C7—N1—C1—C2	−103.9 (2)	Er1—N3—C25—C30	−146.15 (19)
Er1—N1—C1—C2	71.7 (2)	C31—N3—C25—C26	−146.81 (18)
C7—N1—C1—C6	77.5 (2)	Er1—N3—C25—C26	28.9 (2)
Er1—N1—C1—C6	−106.84 (17)	C30—C25—C26—C27	2.0 (3)
C6—C1—C2—C3	1.2 (3)	N3—C25—C26—C27	−173.3 (2)
N1—C1—C2—C3	−177.48 (19)	C25—C26—C27—C28	−1.0 (4)
C1—C2—C3—C4	−0.1 (4)	C26—C27—C28—C29	−0.5 (5)
C2—C3—C4—C5	−0.8 (4)	C27—C28—C29—C30	0.9 (5)
C3—C4—C5—C6	0.6 (3)	C28—C29—C30—C25	0.2 (5)
C4—C5—C6—C1	0.5 (3)	C26—C25—C30—C29	−1.6 (4)
C2—C1—C6—C5	−1.3 (3)	N3—C25—C30—C29	173.6 (2)
N1—C1—C6—C5	177.33 (18)	C25—N3—C31—C36	−140.86 (18)
C1—N1—C7—C8	18.8 (2)	Er1—N3—C31—C36	43.1 (2)
Er1—N1—C7—C8	−156.41 (14)	C25—N3—C31—C32	44.0 (3)
C1—N1—C7—C12	−164.08 (16)	Er1—N3—C31—C32	−131.98 (16)
Er1—N1—C7—C12	20.7 (2)	C36—C31—C32—C33	−0.6 (3)
N1—C7—C8—C9	175.84 (18)	N3—C31—C32—C33	174.59 (19)
C12—C7—C8—C9	−1.3 (3)	C31—C32—C33—C34	−0.7 (3)
C7—C8—C9—C10	0.6 (3)	C32—C33—C34—C35	1.1 (4)
C8—C9—C10—C11	0.3 (3)	C33—C34—C35—C36	−0.1 (4)
C9—C10—C11—C12	−0.2 (3)	C34—C35—C36—C31	−1.2 (3)
C10—C11—C12—C7	−0.6 (3)	C32—C31—C36—C35	1.6 (3)
N1—C7—C12—C11	−175.93 (17)	N3—C31—C36—C35	−173.69 (18)
C8—C7—C12—C11	1.3 (3)	C40—O1—C37—C38B	8.2 (4)
C19—N2—C13—C18	−165.92 (17)	Er1—O1—C37—C38B	−169.3 (4)
Er1—N2—C13—C18	13.5 (3)	C40—O1—C37—C38	−15.1 (6)
C19—N2—C13—C14	16.3 (3)	Er1—O1—C37—C38	167.4 (5)
Er1—N2—C13—C14	−164.33 (14)	O1—C37—C38—C39	−4.4 (9)
N2—C13—C14—C15	176.38 (18)	C37—C38—C39—C40	21.8 (10)
C18—C13—C14—C15	−1.5 (3)	O1—C37—C38B—C39B	−30.9 (5)
C13—C14—C15—C16	−0.1 (3)	C37—C38B—C39B—C40	40.7 (5)
C14—C15—C16—C17	1.4 (4)	C38—C39—C40—O1	−31.4 (8)
C15—C16—C17—C18	−1.1 (4)	C37—O1—C40—C39	30.0 (5)
N2—C13—C18—C17	−176.2 (2)	Er1—O1—C40—C39	−152.7 (5)
C14—C13—C18—C17	1.8 (3)	C37—O1—C40—C39B	17.1 (4)
C16—C17—C18—C13	−0.5 (4)	Er1—O1—C40—C39B	−165.6 (3)
C13—N2—C19—C20	−106.2 (2)	C38B—C39B—C40—O1	−36.4 (5)
Er1—N2—C19—C20	74.27 (19)	C45—O2—C41—C42	−5.6 (2)
C13—N2—C19—C24	77.9 (2)	Er1—O2—C41—C42	179.56 (14)
Er1—N2—C19—C24	−101.57 (17)	O2—C41—C42—C44	23.5 (4)
C24—C19—C20—C21	2.1 (3)	O2—C41—C42—C43	−17.4 (3)
N2—C19—C20—C21	−173.83 (18)	C41—C42—C43—C45	33.5 (3)
C19—C20—C21—C22	−1.2 (3)	C41—C42—C44—C45	−32.0 (5)
C20—C21—C22—C23	−0.8 (3)	C41—O2—C45—C43	27.7 (3)
C21—C22—C23—C24	1.7 (4)	Er1—O2—C45—C43	−157.2 (2)
C22—C23—C24—C19	−0.7 (3)	C41—O2—C45—C44	−13.3 (4)
C20—C19—C24—C23	−1.2 (3)	Er1—O2—C45—C44	161.9 (3)
N2—C19—C24—C23	174.82 (18)	C42—C43—C45—O2	−37.2 (3)
C31—N3—C25—C30	38.1 (3)	C42—C44—C45—O2	28.5 (5)

### Bis[µ-1κN:2(η6)-diphenylamido]bis[bis(diphenylamido-κN)yttrium(III)] (2-Y) Crystal data

**Table:**

[Y_2_(C_12_H_10_N)_6_]	*F*(000) = 1224
*M**_r_* = 1187.08	*D*_x_ = 1.398 Mg m^−^^3^
Monoclinic, *P*2_1_/*c*	Mo *K*α radiation, λ = 0.71073 Å
*a* = 9.2776 (5) Å	Cell parameters from 8538 reflections
*b* = 22.5591 (13) Å	θ = 2.2–28.5°
*c* = 13.4791 (8) Å	µ = 2.10 mm^−^^1^
β = 91.4966 (9)°	*T* = 88 K
*V* = 2820.1 (3) Å^3^	Irregular, colorless
*Z* = 2	0.28 × 0.24 × 0.15 mm

### Bis[µ-1κN:2(η6)-diphenylamido]bis[bis(diphenylamido-κN)yttrium(III)] (2-Y) Data collection

**Table:**

Bruker SMART APEXII CCD diffractometer	5604 reflections with *I* > 2σ(*I*)
Radiation source: fine-focus sealed tube	*R*_int_ = 0.037
φ and ω scans	θ_max_ = 29.0°, θ_min_ = 1.8°
Absorption correction: multi-scan (SADABS; Krause et al., 2015)	*h* = −12→11
*T*_min_ = 0.622, *T*_max_ = 0.746	*k* = −29→29
22940 measured reflections	*l* = −18→18
6851 independent reflections	

### Bis[µ-1κN:2(η6)-diphenylamido]bis[bis(diphenylamido-κN)yttrium(III)] (2-Y) Refinement

**Table:**

Refinement on *F*^2^	Primary atom site location: isomorphous structure methods
Least-squares matrix: full	Secondary atom site location: difference Fourier map
*R*[*F*^2^ > 2σ(*F*^2^)] = 0.032	Hydrogen site location: inferred from neighbouring sites
*wR*(*F*^2^) = 0.070	H-atom parameters constrained
*S* = 1.03	*w* = 1/[σ^2^(*F*_o_^2^) + (0.0256*P*)^2^ + 1.5469*P*] where *P* = (*F*_o_^2^ + 2*F*_c_^2^)/3
6851 reflections	(Δ/σ)_max_ = 0.005
361 parameters	Δρ_max_ = 0.39 e Å^−^^3^
0 restraints	Δρ_min_ = −0.35 e Å^−^^3^

### Bis[µ-1κN:2(η6)-diphenylamido]bis[bis(diphenylamido-κN)yttrium(III)] (2-Y) Special details

**Table:**

Geometry. All esds (except the esd in the dihedral angle between two l.s. planes) are estimated using the full covariance matrix. The cell esds are taken into account individually in the estimation of esds in distances, angles and torsion angles; correlations between esds in cell parameters are only used when they are defined by crystal symmetry. An approximate (isotropic) treatment of cell esds is used for estimating esds involving l.s. planes.
Refinement. A colorless crystal of approximate dimensions 0.146 x 0.239 x 0.284 mm was mounted in a cryoloop and transferred to a Bruker SMART APEX II diffractometer. The APEX2 program package was used to determine the unit-cell parameters and for data collection (30 sec/frame scan time for a hemisphere of diffraction data). The raw frame data was processed using SAINT and SADABS to yield the reflection data file. Subsequent calculations were carried out using the SHELXTL4 program. The diffraction symmetry was 2/m and the systematic absences were consistent with the monoclinic space group P21/c that was later determined to be correct. The structure was solved using the coordinates of the dysprosium analogue and refined on F2 by full-matrix least-squares techniques. The analytical scattering factors for neutral atoms were used throughout the analysis. Hydrogen atoms were included using a riding model. The molecule was located about an inversion center. Least-squares analysis yielded wR2 = 0.0697 and Goof = 1.030 for 361 variables refined against 6851 data (0.73 Å), R1 = 0.0315 for those 5604 data with I > 2.0sigma(I).

### Bis[µ-1κN:2(η6)-diphenylamido]bis[bis(diphenylamido-κN)yttrium(III)] (2-Y) Fractional atomic coordinates and isotropic or equivalent isotropic displacement parameters (Å^2^)

**Table:**

	*x*	*y*	*z*	*U*_iso_*/*U*_eq_	
Y1	0.54944 (2)	0.53325 (2)	0.68672 (2)	0.01043 (5)	
N1	0.31265 (16)	0.46134 (6)	0.45267 (11)	0.0129 (3)	
N2	0.64877 (16)	0.59961 (6)	0.79024 (11)	0.0132 (3)	
N3	0.62857 (16)	0.44684 (6)	0.74806 (11)	0.0123 (3)	
C1	0.30672 (19)	0.49921 (8)	0.53141 (13)	0.0120 (4)	
C2	0.3512 (2)	0.55933 (8)	0.51771 (13)	0.0129 (4)	
H2A	0.3950	0.5701	0.4574	0.016*	
C3	0.3323 (2)	0.60236 (8)	0.58964 (14)	0.0153 (4)	
H3A	0.3573	0.6423	0.5760	0.018*	
C4	0.2766 (2)	0.58774 (8)	0.68228 (14)	0.0165 (4)	
H4A	0.2649	0.6171	0.7320	0.020*	
C5	0.23913 (19)	0.52870 (8)	0.69904 (14)	0.0156 (4)	
H5A	0.2004	0.5180	0.7611	0.019*	
C6	0.2570 (2)	0.48498 (8)	0.62716 (13)	0.0139 (4)	
H6A	0.2356	0.4449	0.6427	0.017*	
C7	0.2581 (2)	0.40201 (8)	0.46423 (13)	0.0130 (4)	
C8	0.3273 (2)	0.35982 (8)	0.52516 (14)	0.0152 (4)	
H8A	0.4088	0.3710	0.5650	0.018*	
C9	0.2776 (2)	0.30176 (8)	0.52768 (15)	0.0185 (4)	
H9A	0.3250	0.2735	0.5694	0.022*	
C10	0.1594 (2)	0.28487 (8)	0.46973 (15)	0.0208 (4)	
H10A	0.1266	0.2450	0.4708	0.025*	
C11	0.0890 (2)	0.32637 (9)	0.41005 (15)	0.0209 (4)	
H11A	0.0073	0.3149	0.3706	0.025*	
C12	0.1375 (2)	0.38481 (8)	0.40774 (14)	0.0160 (4)	
H12A	0.0879	0.4131	0.3673	0.019*	
C13	0.5619 (2)	0.65051 (8)	0.80710 (14)	0.0130 (4)	
C14	0.5344 (2)	0.69157 (8)	0.73156 (14)	0.0154 (4)	
H14A	0.5840	0.6885	0.6710	0.019*	
C15	0.4350 (2)	0.73698 (8)	0.74429 (15)	0.0188 (4)	
H15A	0.4140	0.7635	0.6911	0.023*	
C16	0.3665 (2)	0.74394 (8)	0.83363 (15)	0.0197 (4)	
H16A	0.2990	0.7751	0.8421	0.024*	
C17	0.3979 (2)	0.70446 (8)	0.91100 (15)	0.0179 (4)	
H17A	0.3532	0.7093	0.9731	0.021*	
C18	0.4940 (2)	0.65819 (8)	0.89794 (14)	0.0154 (4)	
H18A	0.5140	0.6315	0.9510	0.019*	
C19	0.7884 (2)	0.60107 (8)	0.83245 (13)	0.0130 (4)	
C20	0.8629 (2)	0.54780 (8)	0.85134 (13)	0.0141 (4)	
H20A	0.8173	0.5111	0.8358	0.017*	
C21	1.0012 (2)	0.54779 (9)	0.89210 (14)	0.0170 (4)	
H21A	1.0484	0.5112	0.9055	0.020*	
C22	1.0721 (2)	0.60080 (9)	0.91365 (14)	0.0189 (4)	
H22A	1.1672	0.6007	0.9415	0.023*	
C23	1.0012 (2)	0.65389 (9)	0.89366 (14)	0.0190 (4)	
H23A	1.0492	0.6904	0.9069	0.023*	
C24	0.8614 (2)	0.65436 (8)	0.85456 (13)	0.0155 (4)	
H24A	0.8143	0.6912	0.8425	0.019*	
C25	0.7441 (2)	0.40688 (8)	0.74930 (13)	0.0124 (4)	
C26	0.8493 (2)	0.40999 (8)	0.67643 (14)	0.0152 (4)	
H26A	0.8444	0.4409	0.6285	0.018*	
C27	0.9602 (2)	0.36881 (9)	0.67311 (15)	0.0190 (4)	
H27A	1.0283	0.3712	0.6218	0.023*	
C28	0.9729 (2)	0.32415 (8)	0.74394 (16)	0.0202 (4)	
H28A	1.0460	0.2948	0.7396	0.024*	
C29	0.8770 (2)	0.32330 (8)	0.82083 (15)	0.0192 (4)	
H29A	0.8884	0.2947	0.8722	0.023*	
C30	0.7640 (2)	0.36378 (8)	0.82387 (14)	0.0149 (4)	
H30A	0.6993	0.3623	0.8772	0.018*	
C31	0.5091 (2)	0.43248 (8)	0.80708 (13)	0.0121 (4)	
C32	0.4588 (2)	0.47383 (8)	0.87565 (13)	0.0140 (4)	
H32A	0.5116	0.5093	0.8880	0.017*	
C33	0.3312 (2)	0.46328 (9)	0.92599 (13)	0.0169 (4)	
H33A	0.2969	0.4921	0.9710	0.020*	
C34	0.2549 (2)	0.41164 (9)	0.91104 (14)	0.0186 (4)	
H34A	0.1683	0.4048	0.9452	0.022*	
C35	0.3065 (2)	0.36940 (9)	0.84486 (14)	0.0185 (4)	
H35A	0.2560	0.3331	0.8356	0.022*	
C36	0.4298 (2)	0.37982 (8)	0.79293 (13)	0.0144 (4)	
H36A	0.4617	0.3511	0.7469	0.017*	

### Bis[µ-1κN:2(η6)-diphenylamido]bis[bis(diphenylamido-κN)yttrium(III)] (2-Y) Atomic displacement parameters (Å^2^)

**Table:**

	*U*^11^	*U*^22^	*U*^33^	*U*^12^	*U*^13^	*U*^23^
Y1	0.01069 (9)	0.00997 (8)	0.01062 (9)	0.00022 (7)	0.00014 (6)	−0.00005 (7)
N1	0.0126 (8)	0.0122 (7)	0.0139 (7)	−0.0011 (6)	0.0014 (6)	0.0014 (6)
N2	0.0136 (8)	0.0108 (7)	0.0150 (8)	0.0010 (6)	−0.0008 (6)	−0.0003 (6)
N3	0.0114 (8)	0.0124 (7)	0.0131 (8)	0.0013 (6)	0.0025 (6)	0.0009 (6)
C1	0.0085 (9)	0.0150 (9)	0.0125 (9)	0.0019 (7)	−0.0012 (7)	0.0005 (7)
C2	0.0117 (9)	0.0148 (9)	0.0124 (9)	0.0011 (7)	0.0013 (7)	0.0017 (7)
C3	0.0133 (10)	0.0145 (9)	0.0179 (9)	0.0018 (7)	−0.0030 (8)	0.0004 (7)
C4	0.0129 (10)	0.0202 (9)	0.0161 (9)	0.0056 (8)	−0.0025 (8)	−0.0057 (7)
C5	0.0090 (9)	0.0245 (10)	0.0134 (9)	0.0024 (8)	0.0007 (7)	−0.0001 (8)
C6	0.0115 (9)	0.0164 (9)	0.0137 (9)	0.0002 (7)	0.0012 (7)	0.0019 (7)
C7	0.0151 (10)	0.0121 (8)	0.0119 (9)	−0.0012 (7)	0.0049 (7)	−0.0002 (7)
C8	0.0129 (10)	0.0167 (9)	0.0162 (9)	−0.0008 (7)	0.0029 (8)	0.0010 (7)
C9	0.0199 (11)	0.0150 (9)	0.0210 (10)	0.0020 (8)	0.0063 (8)	0.0038 (8)
C10	0.0270 (12)	0.0134 (9)	0.0226 (10)	−0.0053 (8)	0.0081 (9)	−0.0012 (8)
C11	0.0210 (11)	0.0238 (10)	0.0180 (10)	−0.0094 (8)	0.0008 (8)	−0.0025 (8)
C12	0.0163 (10)	0.0185 (9)	0.0133 (9)	−0.0003 (8)	0.0017 (8)	0.0021 (7)
C13	0.0107 (9)	0.0112 (8)	0.0168 (9)	−0.0024 (7)	−0.0030 (7)	−0.0017 (7)
C14	0.0176 (10)	0.0127 (9)	0.0159 (9)	−0.0027 (7)	−0.0004 (8)	−0.0004 (7)
C15	0.0206 (11)	0.0127 (9)	0.0229 (10)	0.0003 (8)	−0.0033 (8)	0.0039 (8)
C16	0.0149 (10)	0.0140 (9)	0.0299 (11)	0.0040 (8)	−0.0023 (8)	−0.0010 (8)
C17	0.0174 (10)	0.0187 (9)	0.0177 (10)	0.0006 (8)	0.0022 (8)	−0.0034 (8)
C18	0.0159 (10)	0.0149 (9)	0.0154 (9)	−0.0006 (7)	−0.0015 (8)	0.0012 (7)
C19	0.0141 (10)	0.0167 (9)	0.0082 (8)	−0.0003 (7)	0.0021 (7)	−0.0010 (7)
C20	0.0167 (10)	0.0143 (9)	0.0115 (9)	0.0006 (7)	0.0028 (7)	−0.0015 (7)
C21	0.0160 (10)	0.0204 (9)	0.0148 (9)	0.0039 (8)	0.0033 (8)	−0.0007 (7)
C22	0.0123 (10)	0.0275 (10)	0.0171 (9)	0.0009 (8)	0.0022 (8)	−0.0039 (8)
C23	0.0162 (10)	0.0206 (10)	0.0205 (10)	−0.0035 (8)	0.0024 (8)	−0.0063 (8)
C24	0.0181 (10)	0.0135 (9)	0.0150 (9)	−0.0003 (8)	0.0034 (8)	−0.0015 (7)
C25	0.0115 (9)	0.0109 (8)	0.0146 (9)	−0.0009 (7)	−0.0017 (7)	−0.0033 (7)
C26	0.0137 (10)	0.0166 (9)	0.0153 (9)	−0.0018 (7)	−0.0002 (7)	−0.0007 (7)
C27	0.0110 (10)	0.0240 (10)	0.0222 (10)	−0.0012 (8)	0.0022 (8)	−0.0078 (8)
C28	0.0120 (10)	0.0165 (9)	0.0317 (11)	0.0033 (8)	−0.0055 (8)	−0.0055 (8)
C29	0.0176 (11)	0.0153 (9)	0.0242 (11)	−0.0011 (8)	−0.0067 (8)	0.0019 (8)
C30	0.0150 (10)	0.0150 (9)	0.0148 (9)	−0.0010 (7)	−0.0007 (7)	−0.0015 (7)
C31	0.0110 (9)	0.0143 (9)	0.0108 (8)	0.0020 (7)	−0.0019 (7)	0.0038 (7)
C32	0.0148 (9)	0.0150 (9)	0.0121 (9)	0.0005 (7)	−0.0008 (7)	−0.0001 (7)
C33	0.0158 (10)	0.0225 (9)	0.0123 (9)	0.0060 (8)	0.0002 (7)	0.0014 (8)
C34	0.0112 (10)	0.0285 (11)	0.0161 (9)	−0.0002 (8)	0.0009 (8)	0.0079 (8)
C35	0.0168 (10)	0.0208 (9)	0.0175 (10)	−0.0053 (8)	−0.0041 (8)	0.0046 (8)
C36	0.0152 (10)	0.0149 (9)	0.0132 (9)	0.0004 (7)	−0.0005 (7)	0.0001 (7)

### Bis[µ-1κN:2(η6)-diphenylamido]bis[bis(diphenylamido-κN)yttrium(III)] (2-Y) Geometric parameters (Å, º)

**Table:**

Y1—Cnt	2.584	C14—C15	1.392 (3)
Y1—N2	2.2294 (15)	C14—H14A	0.9500
Y1—N3	2.2340 (15)	C15—C16	1.386 (3)
Y1—N1^i^	2.3039 (15)	C15—H15A	0.9500
Y1—C4	2.8129 (19)	C16—C17	1.396 (3)
Y1—C31	2.8235 (17)	C16—H16A	0.9500
Y1—C3	2.8400 (18)	C17—C18	1.387 (3)
Y1—C5	2.8898 (18)	C17—H17A	0.9500
Y1—C2	2.9498 (18)	C18—H18A	0.9500
Y1—C6	3.0125 (19)	C19—C20	1.406 (2)
Y1—C32	3.0169 (18)	C19—C24	1.408 (2)
Y1—C1	3.1300 (18)	C20—C21	1.383 (3)
N1—C1	1.365 (2)	C20—H20A	0.9500
N1—C7	1.441 (2)	C21—C22	1.392 (3)
N1—Y1^i^	2.3039 (15)	C21—H21A	0.9500
N2—C19	1.401 (2)	C22—C23	1.389 (3)
N2—C13	1.425 (2)	C22—H22A	0.9500
N3—C25	1.400 (2)	C23—C24	1.387 (3)
N3—C31	1.419 (2)	C23—H23A	0.9500
C1—C6	1.419 (3)	C24—H24A	0.9500
C1—C2	1.431 (2)	C25—C26	1.404 (3)
C2—C3	1.386 (3)	C25—C30	1.407 (3)
C2—H2A	0.9500	C26—C27	1.388 (3)
C3—C4	1.403 (3)	C26—H26A	0.9500
C3—H3A	0.9500	C27—C28	1.391 (3)
C4—C5	1.396 (3)	C27—H27A	0.9500
C4—H4A	0.9500	C28—C29	1.384 (3)
C5—C6	1.396 (3)	C28—H28A	0.9500
C5—H5A	0.9500	C29—C30	1.391 (3)
C6—H6A	0.9500	C29—H29A	0.9500
C7—C12	1.392 (3)	C30—H30A	0.9500
C7—C8	1.402 (2)	C31—C32	1.402 (3)
C8—C9	1.389 (3)	C31—C36	1.408 (2)
C8—H8A	0.9500	C32—C33	1.400 (3)
C9—C10	1.383 (3)	C32—H32A	0.9500
C9—H9A	0.9500	C33—C34	1.375 (3)
C10—C11	1.387 (3)	C33—H33A	0.9500
C10—H10A	0.9500	C34—C35	1.398 (3)
C11—C12	1.394 (3)	C34—H34A	0.9500
C11—H11A	0.9500	C35—C36	1.376 (3)
C12—H12A	0.9500	C35—H35A	0.9500
C13—C14	1.395 (2)	C36—H36A	0.9500
C13—C18	1.402 (3)		
			
Cnt—Y1—N1^i^	100.5	C5—C6—H6A	119.4
Cnt—Y1—N2	123.8	C1—C6—H6A	119.4
Cnt—Y1—N3	121.4	Y1—C6—H6A	118.6
N2—Y1—N3	103.17 (5)	C12—C7—C8	118.71 (17)
N2—Y1—N1^i^	104.21 (5)	C12—C7—N1	118.64 (16)
N3—Y1—N1^i^	99.43 (5)	C8—C7—N1	122.51 (16)
N2—Y1—C4	94.41 (5)	C9—C8—C7	120.47 (18)
N3—Y1—C4	132.53 (6)	C9—C8—H8A	119.8
N1^i^—Y1—C4	118.56 (5)	C7—C8—H8A	119.8
N2—Y1—C31	103.88 (5)	C10—C9—C8	120.32 (18)
N3—Y1—C31	29.77 (5)	C10—C9—H9A	119.8
N1^i^—Y1—C31	126.59 (5)	C8—C9—H9A	119.8
C4—Y1—C31	103.38 (6)	C9—C10—C11	119.74 (18)
N2—Y1—C3	101.33 (5)	C9—C10—H10A	120.1
N3—Y1—C3	150.68 (6)	C11—C10—H10A	120.1
N1^i^—Y1—C3	89.86 (5)	C10—C11—C12	120.26 (19)
C4—Y1—C3	28.73 (5)	C10—C11—H11A	119.9
C31—Y1—C3	127.13 (5)	C12—C11—H11A	119.9
N2—Y1—C5	112.58 (6)	C7—C12—C11	120.47 (18)
N3—Y1—C5	105.43 (6)	C7—C12—H12A	119.8
N1^i^—Y1—C5	128.61 (5)	C11—C12—H12A	119.8
C4—Y1—C5	28.31 (5)	C14—C13—C18	118.54 (17)
C31—Y1—C5	77.94 (5)	C14—C13—N2	120.80 (17)
C3—Y1—C5	49.47 (5)	C18—C13—N2	120.51 (16)
N2—Y1—C2	126.06 (5)	C15—C14—C13	120.50 (18)
N3—Y1—C2	130.70 (5)	C15—C14—H14A	119.7
N1^i^—Y1—C2	73.02 (5)	C13—C14—H14A	119.7
C4—Y1—C2	49.74 (5)	C16—C15—C14	120.75 (18)
C31—Y1—C2	121.00 (5)	C16—C15—H15A	119.6
C3—Y1—C2	27.62 (5)	C14—C15—H15A	119.6
C5—Y1—C2	56.59 (5)	C15—C16—C17	119.01 (18)
N2—Y1—C6	139.83 (5)	C15—C16—H16A	120.5
N3—Y1—C6	93.91 (5)	C17—C16—H16A	120.5
N1^i^—Y1—C6	108.49 (5)	C18—C17—C16	120.52 (18)
C4—Y1—C6	49.37 (5)	C18—C17—H17A	119.7
C31—Y1—C6	74.34 (5)	C16—C17—H17A	119.7
C3—Y1—C6	56.81 (5)	C17—C18—C13	120.57 (17)
C5—Y1—C6	27.25 (5)	C17—C18—H18A	119.7
C2—Y1—C6	47.72 (5)	C13—C18—H18A	119.7
N2—Y1—C32	83.68 (5)	N2—C19—C20	119.88 (16)
N3—Y1—C32	52.85 (5)	N2—C19—C24	122.71 (16)
N1^i^—Y1—C32	152.25 (5)	C20—C19—C24	117.37 (17)
C4—Y1—C32	86.65 (5)	C21—C20—C19	121.25 (17)
C31—Y1—C32	27.52 (5)	C21—C20—H20A	119.4
C3—Y1—C32	115.01 (5)	C19—C20—H20A	119.4
C5—Y1—C32	68.62 (5)	C20—C21—C22	120.75 (18)
C2—Y1—C32	123.95 (5)	C20—C21—H21A	119.6
C6—Y1—C32	78.19 (5)	C22—C21—H21A	119.6
N2—Y1—C1	149.52 (5)	C23—C22—C21	118.79 (18)
N3—Y1—C1	104.90 (5)	C23—C22—H22A	120.6
N1^i^—Y1—C1	82.69 (5)	C21—C22—H22A	120.6
C4—Y1—C1	57.44 (5)	C24—C23—C22	120.89 (18)
C31—Y1—C1	94.75 (5)	C24—C23—H23A	119.6
C3—Y1—C1	48.48 (5)	C22—C23—H23A	119.6
C5—Y1—C1	47.88 (5)	C23—C24—C19	120.91 (17)
C2—Y1—C1	27.02 (5)	C23—C24—H24A	119.5
C6—Y1—C1	26.62 (5)	C19—C24—H24A	119.5
C32—Y1—C1	104.12 (5)	N3—C25—C26	120.34 (16)
C1—N1—C7	118.43 (15)	N3—C25—C30	122.66 (17)
C1—N1—Y1^i^	129.47 (12)	C26—C25—C30	116.97 (17)
C7—N1—Y1^i^	109.96 (11)	C27—C26—C25	121.22 (18)
C19—N2—C13	115.92 (14)	C27—C26—H26A	119.4
C19—N2—Y1	129.23 (12)	C25—C26—H26A	119.4
C13—N2—Y1	114.50 (11)	C26—C27—C28	120.82 (19)
C25—N3—C31	117.09 (15)	C26—C27—H27A	119.6
C25—N3—Y1	144.12 (12)	C28—C27—H27A	119.6
C31—N3—Y1	98.78 (10)	C29—C28—C27	118.65 (18)
N1—C1—C6	125.92 (16)	C29—C28—H28A	120.7
N1—C1—C2	118.38 (16)	C27—C28—H28A	120.7
C6—C1—C2	115.69 (16)	C28—C29—C30	120.84 (18)
N1—C1—Y1	129.11 (12)	C28—C29—H29A	119.6
C6—C1—Y1	72.07 (10)	C30—C29—H29A	119.6
C2—C1—Y1	69.46 (10)	C29—C30—C25	121.16 (18)
C3—C2—C1	122.09 (17)	C29—C30—H30A	119.4
C3—C2—Y1	71.78 (10)	C25—C30—H30A	119.4
C1—C2—Y1	83.53 (10)	C32—C31—C36	117.97 (17)
C3—C2—H2A	119.0	C32—C31—N3	119.75 (16)
C1—C2—H2A	119.0	C36—C31—N3	122.01 (16)
Y1—C2—H2A	116.1	C32—C31—Y1	83.94 (11)
C2—C3—C4	120.94 (17)	C36—C31—Y1	132.71 (12)
C2—C3—Y1	80.60 (11)	N3—C31—Y1	51.44 (8)
C4—C3—Y1	74.56 (10)	C33—C32—C31	120.45 (17)
C2—C3—H3A	119.5	C33—C32—Y1	137.96 (12)
C4—C3—H3A	119.5	C31—C32—Y1	68.54 (10)
Y1—C3—H3A	115.8	C33—C32—H32A	119.8
C5—C4—C3	117.86 (17)	C31—C32—H32A	119.8
C5—C4—Y1	78.91 (11)	Y1—C32—H32A	67.7
C3—C4—Y1	76.71 (11)	C34—C33—C32	120.75 (18)
C5—C4—H4A	121.1	C34—C33—H33A	119.6
C3—C4—H4A	121.1	C32—C33—H33A	119.6
Y1—C4—H4A	114.2	C33—C34—C35	119.16 (18)
C6—C5—C4	121.80 (17)	C33—C34—H34A	120.4
C6—C5—Y1	81.28 (11)	C35—C34—H34A	120.4
C4—C5—Y1	72.79 (11)	C36—C35—C34	120.73 (18)
C6—C5—H5A	119.1	C36—C35—H35A	119.6
C4—C5—H5A	119.1	C34—C35—H35A	119.6
Y1—C5—H5A	117.4	C35—C36—C31	120.90 (17)
C5—C6—C1	121.28 (17)	C35—C36—H36A	119.6
C5—C6—Y1	71.47 (11)	C31—C36—H36A	119.6
C1—C6—Y1	81.32 (11)		
			
C7—N1—C1—C6	3.1 (3)	C14—C15—C16—C17	−0.3 (3)
Y1^i^—N1—C1—C6	−158.49 (14)	C15—C16—C17—C18	−1.4 (3)
C7—N1—C1—C2	−175.55 (16)	C16—C17—C18—C13	0.4 (3)
Y1^i^—N1—C1—C2	22.9 (2)	C14—C13—C18—C17	2.2 (3)
C7—N1—C1—Y1	98.74 (18)	N2—C13—C18—C17	−173.45 (17)
Y1^i^—N1—C1—Y1	−62.81 (19)	C13—N2—C19—C20	157.34 (16)
N1—C1—C2—C3	171.99 (17)	Y1—N2—C19—C20	−29.9 (2)
C6—C1—C2—C3	−6.8 (3)	C13—N2—C19—C24	−24.8 (2)
Y1—C1—C2—C3	−63.73 (16)	Y1—N2—C19—C24	148.01 (14)
N1—C1—C2—Y1	−124.28 (15)	N2—C19—C20—C21	179.41 (17)
C6—C1—C2—Y1	56.97 (15)	C24—C19—C20—C21	1.4 (3)
C1—C2—C3—C4	4.2 (3)	C19—C20—C21—C22	−1.4 (3)
Y1—C2—C3—C4	−65.55 (16)	C20—C21—C22—C23	0.1 (3)
C1—C2—C3—Y1	69.72 (16)	C21—C22—C23—C24	1.1 (3)
C2—C3—C4—C5	−0.9 (3)	C22—C23—C24—C19	−1.1 (3)
Y1—C3—C4—C5	−69.60 (15)	N2—C19—C24—C23	−178.09 (17)
C2—C3—C4—Y1	68.71 (16)	C20—C19—C24—C23	−0.2 (3)
C3—C4—C5—C6	0.6 (3)	C31—N3—C25—C26	164.66 (16)
Y1—C4—C5—C6	−67.73 (17)	Y1—N3—C25—C26	−16.9 (3)
C3—C4—C5—Y1	68.36 (15)	C31—N3—C25—C30	−17.7 (2)
C4—C5—C6—C1	−3.6 (3)	Y1—N3—C25—C30	160.73 (15)
Y1—C5—C6—C1	−67.05 (16)	N3—C25—C26—C27	−176.41 (17)
C4—C5—C6—Y1	63.42 (16)	C30—C25—C26—C27	5.8 (3)
N1—C1—C6—C5	−172.21 (17)	C25—C26—C27—C28	−2.0 (3)
C2—C1—C6—C5	6.4 (3)	C26—C27—C28—C29	−3.2 (3)
Y1—C1—C6—C5	62.04 (16)	C27—C28—C29—C30	4.3 (3)
N1—C1—C6—Y1	125.75 (18)	C28—C29—C30—C25	−0.2 (3)
C2—C1—C6—Y1	−55.60 (14)	N3—C25—C30—C29	177.55 (17)
C1—N1—C7—C12	114.78 (19)	C26—C25—C30—C29	−4.8 (3)
Y1^i^—N1—C7—C12	−80.29 (18)	C25—N3—C31—C32	126.76 (17)
C1—N1—C7—C8	−69.5 (2)	Y1—N3—C31—C32	−52.32 (17)
Y1^i^—N1—C7—C8	95.41 (17)	C25—N3—C31—C36	−59.3 (2)
C12—C7—C8—C9	1.1 (3)	Y1—N3—C31—C36	121.64 (15)
N1—C7—C8—C9	−174.64 (17)	C25—N3—C31—Y1	179.08 (17)
C7—C8—C9—C10	0.2 (3)	C36—C31—C32—C33	−1.7 (3)
C8—C9—C10—C11	−1.0 (3)	N3—C31—C32—C33	172.48 (16)
C9—C10—C11—C12	0.5 (3)	Y1—C31—C32—C33	133.99 (16)
C8—C7—C12—C11	−1.6 (3)	C36—C31—C32—Y1	−135.72 (16)
N1—C7—C12—C11	174.28 (17)	N3—C31—C32—Y1	38.49 (13)
C10—C11—C12—C7	0.8 (3)	C31—C32—C33—C34	1.6 (3)
C19—N2—C13—C14	105.47 (19)	Y1—C32—C33—C34	92.7 (2)
Y1—N2—C13—C14	−68.39 (19)	C32—C33—C34—C35	0.2 (3)
C19—N2—C13—C18	−79.0 (2)	C33—C34—C35—C36	−1.9 (3)
Y1—N2—C13—C18	107.16 (16)	C34—C35—C36—C31	1.7 (3)
C18—C13—C14—C15	−3.9 (3)	C32—C31—C36—C35	0.1 (3)
N2—C13—C14—C15	171.74 (17)	N3—C31—C36—C35	−174.01 (17)
C13—C14—C15—C16	3.0 (3)	Y1—C31—C36—C35	−109.05 (19)

Symmetry code: (i) −*x*+1, −*y*+1, −*z*+1.

### Bis[µ-1κN:2(η6)-diphenylamido]bis[bis(diphenylamido-κN)dysprosium(III)] (2-Dy) Crystal data

**Table:**

[Dy_2_(C_12_H_10_N)_6_]	*F*(000) = 1332
*M**_r_* = 1334.26	*D*_x_ = 1.568 Mg m^−^^3^
Monoclinic, *P*2_1_/*c*	Mo *K*α radiation, λ = 0.71073 Å
*a* = 9.3068 (15) Å	Cell parameters from 9898 reflections
*b* = 22.475 (4) Å	θ = 2.4–29.1°
*c* = 13.513 (2) Å	µ = 2.67 mm^−^^1^
β = 91.266 (2)°	*T* = 88 K
*V* = 2825.8 (8) Å^3^	Rectangle, yellow
*Z* = 2	0.20 × 0.12 × 0.11 mm

### Bis[µ-1κN:2(η6)-diphenylamido]bis[bis(diphenylamido-κN)dysprosium(III)] (2-Dy) Data collection

**Table:**

Bruker SMART APEXII CCD diffractometer	6207 reflections with *I* > 2σ(*I*)
Radiation source: fine-focus sealed tube	*R*_int_ = 0.037
φ and ω scans	θ_max_ = 29.2°, θ_min_ = 1.8°
Absorption correction: multi-scan (SADABS; Krause et al., 2015)	*h* = −12→12
*T*_min_ = 0.637, *T*_max_ = 0.746	*k* = −29→30
34856 measured reflections	*l* = −18→18
7264 independent reflections	

### Bis[µ-1κN:2(η6)-diphenylamido]bis[bis(diphenylamido-κN)dysprosium(III)] (2-Dy) Refinement

**Table:**

Refinement on *F*^2^	Primary atom site location: structure-invariant direct methods
Least-squares matrix: full	Secondary atom site location: difference Fourier map
*R*[*F*^2^ > 2σ(*F*^2^)] = 0.026	Hydrogen site location: inferred from neighbouring sites
*wR*(*F*^2^) = 0.065	H-atom parameters constrained
*S* = 1.05	*w* = 1/[σ^2^(*F*_o_^2^) + (0.0347*P*)^2^ + 1.5774*P*] where *P* = (*F*_o_^2^ + 2*F*_c_^2^)/3
7264 reflections	(Δ/σ)_max_ = 0.004
361 parameters	Δρ_max_ = 2.65 e Å^−^^3^
0 restraints	Δρ_min_ = −0.81 e Å^−^^3^

### Bis[µ-1κN:2(η6)-diphenylamido]bis[bis(diphenylamido-κN)dysprosium(III)] (2-Dy) Special details

**Table:**

Geometry. All esds (except the esd in the dihedral angle between two l.s. planes) are estimated using the full covariance matrix. The cell esds are taken into account individually in the estimation of esds in distances, angles and torsion angles; correlations between esds in cell parameters are only used when they are defined by crystal symmetry. An approximate (isotropic) treatment of cell esds is used for estimating esds involving l.s. planes.
Refinement. A yellow crystal of approximate dimensions 0.111 x 0.116 x 0.201 mm was mounted in a cryoloop and transferred to a Bruker SMART APEX II diffractometer. The APEX2 program package was used to determine the unit-cell parameters and for data collection (20 sec/frame scan time for a sphere of diffraction data). The raw frame data was processed using SAINT and SADABS to yield the reflection data file. Subsequent calculations were carried out using the SHELXTL program. The diffraction symmetry was 2/m and the systematic absences were consistent with the monoclinic space group P21/c that was later determined to be correct. The structure was solved by direct methods and refined on F2 by full-matrix least-squares techniques. The analytical scattering factors for neutral atoms were used throughout the analysis. Hydrogen atoms were included using a riding model. The molecule was located about an inversion center. Least-squares analysis yielded wR2 = 0.0652 and Goof = 1.046 for 361 variables refined against 7264 data (0.73 Å), R1 = 0.0255 for those 6207 data with I > 2.0sigma(I).

### Bis[µ-1κN:2(η6)-diphenylamido]bis[bis(diphenylamido-κN)dysprosium(III)] (2-Dy) Fractional atomic coordinates and isotropic or equivalent isotropic displacement parameters (Å^2^)

**Table:**

	*x*	*y*	*z*	*U*_iso_*/*U*_eq_	
Dy1	0.55102 (2)	0.53447 (2)	0.68682 (2)	0.01129 (4)	
N1	0.3123 (2)	0.46206 (8)	0.45361 (15)	0.0138 (4)	
N2	0.6513 (2)	0.59985 (9)	0.79130 (15)	0.0139 (4)	
N3	0.6285 (2)	0.44733 (9)	0.74828 (15)	0.0138 (4)	
C1	0.3069 (2)	0.50018 (11)	0.53188 (17)	0.0134 (5)	
C2	0.3516 (3)	0.56042 (11)	0.51798 (18)	0.0146 (5)	
H2A	0.3959	0.5711	0.4579	0.018*	
C3	0.3323 (3)	0.60366 (11)	0.58969 (18)	0.0160 (5)	
H3A	0.3571	0.6438	0.5759	0.019*	
C4	0.2765 (3)	0.58902 (11)	0.68239 (19)	0.0167 (5)	
H4A	0.2640	0.6185	0.7319	0.020*	
C5	0.2401 (3)	0.52961 (11)	0.69925 (19)	0.0174 (5)	
H5A	0.2022	0.5188	0.7614	0.021*	
C6	0.2576 (3)	0.48579 (11)	0.62774 (18)	0.0159 (5)	
H6A	0.2363	0.4456	0.6433	0.019*	
C7	0.2582 (3)	0.40265 (11)	0.46475 (17)	0.0143 (5)	
C8	0.3269 (3)	0.36010 (11)	0.52499 (19)	0.0165 (5)	
H8A	0.4081	0.3712	0.5647	0.020*	
C9	0.2771 (3)	0.30160 (11)	0.5272 (2)	0.0204 (5)	
H9A	0.3240	0.2731	0.5686	0.024*	
C10	0.1591 (3)	0.28486 (12)	0.4689 (2)	0.0218 (6)	
H10A	0.1259	0.2449	0.4698	0.026*	
C11	0.0900 (3)	0.32678 (12)	0.4093 (2)	0.0219 (5)	
H11A	0.0093	0.3154	0.3695	0.026*	
C12	0.1381 (3)	0.38532 (11)	0.40750 (18)	0.0180 (5)	
H12A	0.0892	0.4138	0.3671	0.022*	
C13	0.5638 (2)	0.65068 (10)	0.80820 (18)	0.0140 (5)	
C14	0.5361 (3)	0.69171 (11)	0.73263 (19)	0.0169 (5)	
H14A	0.5859	0.6887	0.6722	0.020*	
C15	0.4358 (3)	0.73717 (12)	0.7450 (2)	0.0208 (5)	
H15A	0.4147	0.7637	0.6919	0.025*	
C16	0.3668 (3)	0.74397 (11)	0.8343 (2)	0.0199 (5)	
H16A	0.2987	0.7750	0.8426	0.024*	
C17	0.3988 (3)	0.70468 (12)	0.9116 (2)	0.0198 (5)	
H17A	0.3541	0.7096	0.9736	0.024*	
C18	0.4956 (3)	0.65840 (11)	0.89879 (18)	0.0155 (5)	
H18A	0.5157	0.6317	0.9519	0.019*	
C19	0.7902 (3)	0.60156 (11)	0.83350 (17)	0.0137 (5)	
C20	0.8640 (3)	0.54784 (11)	0.85176 (18)	0.0151 (5)	
H20A	0.8187	0.5111	0.8358	0.018*	
C21	1.0022 (3)	0.54794 (12)	0.89280 (19)	0.0178 (5)	
H21A	1.0491	0.5112	0.9063	0.021*	
C22	1.0730 (3)	0.60102 (12)	0.91443 (19)	0.0202 (5)	
H22A	1.1675	0.6008	0.9424	0.024*	
C23	1.0027 (3)	0.65426 (12)	0.89423 (19)	0.0196 (5)	
H23A	1.0506	0.6909	0.9071	0.024*	
C24	0.8628 (3)	0.65475 (11)	0.85541 (18)	0.0167 (5)	
H24A	0.8158	0.6917	0.8436	0.020*	
C25	0.7434 (2)	0.40709 (10)	0.74912 (18)	0.0131 (5)	
C26	0.8493 (3)	0.41048 (11)	0.67664 (19)	0.0165 (5)	
H26A	0.8451	0.4416	0.6292	0.020*	
C27	0.9598 (3)	0.36907 (12)	0.6732 (2)	0.0207 (5)	
H27A	1.0279	0.3714	0.6220	0.025*	
C28	0.9724 (3)	0.32419 (12)	0.7439 (2)	0.0224 (6)	
H28A	1.0454	0.2948	0.7396	0.027*	
C29	0.8761 (3)	0.32334 (12)	0.8204 (2)	0.0211 (5)	
H29A	0.8873	0.2946	0.8716	0.025*	
C30	0.7631 (3)	0.36389 (11)	0.82377 (19)	0.0164 (5)	
H30A	0.6984	0.3624	0.8771	0.020*	
C31	0.5088 (3)	0.43281 (11)	0.80700 (17)	0.0130 (5)	
C32	0.4586 (3)	0.47398 (11)	0.87570 (18)	0.0148 (5)	
H32A	0.5113	0.5095	0.8882	0.018*	
C33	0.3314 (3)	0.46332 (11)	0.92619 (19)	0.0179 (5)	
H33A	0.2976	0.4921	0.9717	0.022*	
C34	0.2549 (3)	0.41167 (12)	0.91064 (19)	0.0205 (5)	
H34A	0.1683	0.4048	0.9448	0.025*	
C35	0.3055 (3)	0.36945 (12)	0.84425 (19)	0.0190 (5)	
H35A	0.2544	0.3332	0.8345	0.023*	
C36	0.4298 (3)	0.38005 (11)	0.79245 (18)	0.0155 (5)	
H36A	0.4622	0.3513	0.7465	0.019*	

### Bis[µ-1κN:2(η6)-diphenylamido]bis[bis(diphenylamido-κN)dysprosium(III)] (2-Dy) Atomic displacement parameters (Å^2^)

**Table:**

	*U*^11^	*U*^22^	*U*^33^	*U*^12^	*U*^13^	*U*^23^
Dy1	0.01278 (6)	0.00898 (6)	0.01212 (6)	0.00032 (4)	0.00080 (4)	−0.00024 (4)
N1	0.0153 (10)	0.0108 (10)	0.0152 (10)	−0.0026 (8)	0.0009 (8)	0.0011 (8)
N2	0.0159 (10)	0.0090 (9)	0.0168 (10)	−0.0003 (8)	0.0000 (8)	−0.0017 (8)
N3	0.0153 (10)	0.0101 (9)	0.0162 (10)	0.0014 (8)	0.0024 (8)	0.0020 (8)
C1	0.0117 (11)	0.0145 (12)	0.0141 (11)	0.0011 (9)	0.0004 (9)	0.0012 (9)
C2	0.0135 (11)	0.0149 (12)	0.0155 (12)	0.0008 (9)	0.0010 (9)	0.0010 (9)
C3	0.0149 (11)	0.0128 (11)	0.0201 (12)	0.0017 (9)	−0.0041 (9)	0.0001 (10)
C4	0.0145 (11)	0.0190 (13)	0.0165 (12)	0.0034 (9)	−0.0012 (9)	−0.0046 (9)
C5	0.0137 (11)	0.0234 (14)	0.0150 (12)	0.0022 (10)	0.0012 (9)	0.0012 (10)
C6	0.0150 (12)	0.0162 (12)	0.0165 (12)	0.0001 (10)	0.0026 (9)	0.0027 (10)
C7	0.0164 (11)	0.0118 (11)	0.0150 (11)	−0.0007 (9)	0.0057 (9)	−0.0007 (9)
C8	0.0152 (11)	0.0161 (12)	0.0183 (12)	−0.0003 (9)	0.0012 (9)	0.0031 (9)
C9	0.0249 (14)	0.0134 (12)	0.0232 (13)	0.0015 (10)	0.0074 (11)	0.0042 (10)
C10	0.0277 (14)	0.0137 (12)	0.0243 (14)	−0.0048 (11)	0.0081 (11)	−0.0015 (10)
C11	0.0232 (13)	0.0209 (14)	0.0215 (13)	−0.0084 (11)	0.0010 (10)	−0.0014 (11)
C12	0.0207 (12)	0.0176 (13)	0.0159 (12)	−0.0025 (10)	0.0010 (10)	0.0019 (10)
C13	0.0136 (11)	0.0095 (11)	0.0190 (12)	−0.0025 (9)	−0.0005 (9)	−0.0022 (9)
C14	0.0216 (13)	0.0121 (12)	0.0169 (12)	−0.0016 (10)	0.0003 (10)	0.0007 (9)
C15	0.0248 (13)	0.0137 (12)	0.0236 (13)	0.0002 (10)	−0.0049 (11)	0.0032 (10)
C16	0.0170 (12)	0.0139 (12)	0.0286 (14)	0.0017 (10)	−0.0003 (10)	−0.0007 (10)
C17	0.0181 (12)	0.0188 (13)	0.0226 (13)	0.0012 (10)	0.0037 (10)	0.0000 (10)
C18	0.0160 (12)	0.0139 (12)	0.0165 (12)	−0.0004 (9)	−0.0014 (9)	0.0014 (9)
C19	0.0147 (11)	0.0140 (12)	0.0124 (11)	0.0005 (9)	0.0036 (9)	−0.0026 (9)
C20	0.0163 (12)	0.0118 (11)	0.0173 (12)	−0.0007 (9)	0.0047 (9)	−0.0006 (9)
C21	0.0164 (12)	0.0198 (13)	0.0173 (12)	0.0050 (10)	0.0045 (10)	−0.0008 (10)
C22	0.0151 (12)	0.0274 (14)	0.0182 (12)	0.0015 (11)	0.0013 (10)	−0.0020 (11)
C23	0.0168 (12)	0.0201 (13)	0.0221 (13)	−0.0041 (10)	0.0042 (10)	−0.0058 (10)
C24	0.0177 (12)	0.0143 (12)	0.0183 (12)	−0.0013 (10)	0.0038 (10)	−0.0015 (10)
C25	0.0127 (11)	0.0103 (11)	0.0163 (11)	−0.0008 (9)	−0.0027 (9)	−0.0040 (9)
C26	0.0159 (12)	0.0160 (12)	0.0177 (12)	−0.0013 (9)	0.0001 (9)	−0.0015 (9)
C27	0.0138 (12)	0.0215 (13)	0.0270 (14)	−0.0023 (10)	0.0016 (10)	−0.0074 (11)
C28	0.0137 (12)	0.0181 (13)	0.0353 (15)	0.0039 (10)	−0.0043 (11)	−0.0074 (11)
C29	0.0210 (13)	0.0150 (12)	0.0268 (14)	0.0006 (10)	−0.0085 (11)	0.0034 (10)
C30	0.0177 (12)	0.0150 (12)	0.0165 (12)	−0.0006 (10)	−0.0016 (9)	−0.0002 (9)
C31	0.0133 (11)	0.0128 (12)	0.0129 (11)	0.0021 (9)	0.0009 (9)	0.0008 (9)
C32	0.0173 (12)	0.0125 (12)	0.0148 (12)	0.0002 (9)	0.0004 (9)	0.0004 (9)
C33	0.0161 (12)	0.0224 (13)	0.0154 (12)	0.0064 (10)	0.0019 (10)	0.0005 (10)
C34	0.0157 (12)	0.0278 (15)	0.0178 (13)	−0.0001 (10)	0.0013 (10)	0.0073 (10)
C35	0.0185 (12)	0.0195 (13)	0.0187 (13)	−0.0047 (10)	−0.0045 (10)	0.0037 (10)
C36	0.0188 (12)	0.0130 (12)	0.0145 (12)	0.0006 (9)	0.0000 (9)	0.0007 (9)

### Bis[µ-1κN:2(η6)-diphenylamido]bis[bis(diphenylamido-κN)dysprosium(III)] (2-Dy) Geometric parameters (Å, º)

**Table:**

Dy1—Cnt	2.605	C14—C15	1.396 (4)
Dy1—N2	2.228 (2)	C14—H14A	0.9500
Dy1—N3	2.240 (2)	C15—C16	1.387 (4)
Dy1—N1^i^	2.309 (2)	C15—H15A	0.9500
Dy1—C4	2.833 (2)	C16—C17	1.395 (4)
Dy1—C31	2.836 (2)	C16—H16A	0.9500
Dy1—C3	2.858 (2)	C17—C18	1.389 (3)
Dy1—C5	2.904 (3)	C17—H17A	0.9500
Dy1—C2	2.967 (2)	C18—H18A	0.9500
Dy1—C6	3.032 (3)	C19—C24	1.401 (3)
Dy1—C1	3.151 (2)	C19—C20	1.408 (3)
N1—C1	1.363 (3)	C20—C21	1.390 (4)
N1—C7	1.436 (3)	C20—H20A	0.9500
N1—Dy1^i^	2.309 (2)	C21—C22	1.391 (4)
N2—C19	1.402 (3)	C21—H21A	0.9500
N2—C13	1.424 (3)	C22—C23	1.388 (4)
N3—C25	1.400 (3)	C22—H22A	0.9500
N3—C31	1.420 (3)	C23—C24	1.393 (3)
C1—C6	1.421 (3)	C23—H23A	0.9500
C1—C2	1.430 (3)	C24—H24A	0.9500
C2—C3	1.387 (3)	C25—C26	1.407 (3)
C2—H2A	0.9500	C25—C30	1.409 (3)
C3—C4	1.406 (3)	C26—C27	1.389 (4)
C3—H3A	0.9500	C26—H26A	0.9500
C4—C5	1.398 (4)	C27—C28	1.392 (4)
C4—H4A	0.9500	C27—H27A	0.9500
C5—C6	1.392 (4)	C28—C29	1.384 (4)
C5—H5A	0.9500	C28—H28A	0.9500
C6—H6A	0.9500	C29—C30	1.393 (4)
C7—C12	1.400 (3)	C29—H29A	0.9500
C7—C8	1.401 (3)	C30—H30A	0.9500
C8—C9	1.395 (3)	C31—C32	1.398 (3)
C8—H8A	0.9500	C31—C36	1.407 (3)
C9—C10	1.388 (4)	C32—C33	1.400 (4)
C9—H9A	0.9500	C32—H32A	0.9500
C10—C11	1.388 (4)	C33—C34	1.376 (4)
C10—H10A	0.9500	C33—H33A	0.9500
C11—C12	1.390 (4)	C34—C35	1.395 (4)
C11—H11A	0.9500	C34—H34A	0.9500
C12—H12A	0.9500	C35—C36	1.387 (3)
C13—C14	1.396 (3)	C35—H35A	0.9500
C13—C18	1.402 (3)	C36—H36A	0.9500
			
Cnt—Dy1—N1^i^	100.3	C12—C7—C8	118.7 (2)
Cnt—Dy1—N2	124.6	C12—C7—N1	118.6 (2)
Cnt—Dy1—N3	120.9	C8—C7—N1	122.6 (2)
N2—Dy1—N3	102.41 (7)	C9—C8—C7	120.5 (2)
N2—Dy1—N1^i^	105.54 (7)	C9—C8—H8A	119.7
N3—Dy1—N1^i^	98.95 (7)	C7—C8—H8A	119.7
N2—Dy1—C4	95.33 (7)	C10—C9—C8	120.2 (2)
N3—Dy1—C4	131.97 (7)	C10—C9—H9A	119.9
N1^i^—Dy1—C4	118.75 (7)	C8—C9—H9A	119.9
N2—Dy1—C31	103.29 (7)	C11—C10—C9	119.7 (2)
N3—Dy1—C31	29.63 (7)	C11—C10—H10A	120.2
N1^i^—Dy1—C31	125.71 (7)	C9—C10—H10A	120.2
C4—Dy1—C31	102.97 (7)	C10—C11—C12	120.5 (2)
N2—Dy1—C3	102.49 (7)	C10—C11—H11A	119.7
N3—Dy1—C3	150.00 (7)	C12—C11—H11A	119.7
N1^i^—Dy1—C3	90.23 (7)	C11—C12—C7	120.4 (2)
C4—Dy1—C3	28.60 (7)	C11—C12—H12A	119.8
C31—Dy1—C3	126.53 (7)	C7—C12—H12A	119.8
N2—Dy1—C5	113.08 (7)	C14—C13—C18	118.5 (2)
N3—Dy1—C5	104.98 (7)	C14—C13—N2	120.6 (2)
N1^i^—Dy1—C5	128.06 (7)	C18—C13—N2	120.7 (2)
C4—Dy1—C5	28.16 (7)	C13—C14—C15	120.6 (2)
C31—Dy1—C5	77.63 (7)	C13—C14—H14A	119.7
C3—Dy1—C5	49.20 (7)	C15—C14—H14A	119.7
N2—Dy1—C2	127.14 (7)	C16—C15—C14	120.6 (2)
N3—Dy1—C2	130.36 (7)	C16—C15—H15A	119.7
N1^i^—Dy1—C2	73.02 (7)	C14—C15—H15A	119.7
C4—Dy1—C2	49.48 (7)	C15—C16—C17	119.1 (2)
C31—Dy1—C2	120.44 (7)	C15—C16—H16A	120.5
C3—Dy1—C2	27.47 (7)	C17—C16—H16A	120.5
C5—Dy1—C2	56.28 (7)	C18—C17—C16	120.6 (2)
N2—Dy1—C6	140.08 (7)	C18—C17—H17A	119.7
N3—Dy1—C6	93.59 (7)	C16—C17—H17A	119.7
N1^i^—Dy1—C6	107.76 (7)	C17—C18—C13	120.6 (2)
C4—Dy1—C6	49.02 (7)	C17—C18—H18A	119.7
C31—Dy1—C6	74.02 (7)	C13—C18—H18A	119.7
C3—Dy1—C6	56.45 (7)	C24—C19—N2	123.0 (2)
C5—Dy1—C6	27.01 (7)	C24—C19—C20	117.6 (2)
C2—Dy1—C6	47.48 (7)	N2—C19—C20	119.3 (2)
N2—Dy1—C1	150.23 (7)	C21—C20—C19	120.8 (2)
N3—Dy1—C1	104.70 (7)	C21—C20—H20A	119.6
N1^i^—Dy1—C1	82.21 (7)	C19—C20—H20A	119.6
C4—Dy1—C1	56.99 (7)	C20—C21—C22	121.0 (2)
C31—Dy1—C1	94.39 (7)	C20—C21—H21A	119.5
C3—Dy1—C1	48.09 (7)	C22—C21—H21A	119.5
C5—Dy1—C1	47.51 (7)	C23—C22—C21	118.6 (2)
C2—Dy1—C1	26.82 (7)	C23—C22—H22A	120.7
C6—Dy1—C1	26.48 (6)	C21—C22—H22A	120.7
C1—N1—C7	118.9 (2)	C22—C23—C24	120.9 (2)
C1—N1—Dy1^i^	130.32 (16)	C22—C23—H23A	119.5
C7—N1—Dy1^i^	108.57 (14)	C24—C23—H23A	119.5
C19—N2—C13	116.00 (19)	C23—C24—C19	121.0 (2)
C19—N2—Dy1	130.18 (15)	C23—C24—H24A	119.5
C13—N2—Dy1	113.43 (15)	C19—C24—H24A	119.5
C25—N3—C31	117.1 (2)	N3—C25—C26	120.4 (2)
C25—N3—Dy1	143.80 (16)	N3—C25—C30	122.5 (2)
C31—N3—Dy1	99.10 (14)	C26—C25—C30	117.0 (2)
N1—C1—C6	125.6 (2)	C27—C26—C25	121.2 (2)
N1—C1—C2	118.5 (2)	C27—C26—H26A	119.4
C6—C1—C2	115.9 (2)	C25—C26—H26A	119.4
N1—C1—Dy1	129.12 (16)	C26—C27—C28	120.9 (2)
C6—C1—Dy1	72.09 (14)	C26—C27—H27A	119.5
C2—C1—Dy1	69.39 (13)	C28—C27—H27A	119.5
C3—C2—C1	121.9 (2)	C29—C28—C27	118.5 (2)
C3—C2—Dy1	71.89 (14)	C29—C28—H28A	120.8
C1—C2—Dy1	83.80 (14)	C27—C28—H28A	120.8
C3—C2—H2A	119.0	C28—C29—C30	121.1 (2)
C1—C2—H2A	119.0	C28—C29—H29A	119.4
Dy1—C2—H2A	115.6	C30—C29—H29A	119.4
C2—C3—C4	121.0 (2)	C29—C30—C25	120.9 (2)
C2—C3—Dy1	80.64 (14)	C29—C30—H30A	119.5
C4—C3—Dy1	74.73 (14)	C25—C30—H30A	119.5
C2—C3—H3A	119.5	C32—C31—C36	118.0 (2)
C4—C3—H3A	119.5	C32—C31—N3	119.8 (2)
Dy1—C3—H3A	115.6	C36—C31—N3	122.0 (2)
C5—C4—C3	117.7 (2)	C32—C31—Dy1	84.24 (15)
C5—C4—Dy1	78.75 (14)	C36—C31—Dy1	132.67 (17)
C3—C4—Dy1	76.67 (14)	N3—C31—Dy1	51.27 (11)
C5—C4—H4A	121.2	C31—C32—C33	120.6 (2)
C3—C4—H4A	121.2	C31—C32—H32A	119.7
Dy1—C4—H4A	114.3	C33—C32—H32A	119.7
C6—C5—C4	122.0 (2)	C34—C33—C32	120.7 (2)
C6—C5—Dy1	81.62 (15)	C34—C33—H33A	119.7
C4—C5—Dy1	73.09 (14)	C32—C33—H33A	119.7
C6—C5—H5A	119.0	C33—C34—C35	119.4 (2)
C4—C5—H5A	119.0	C33—C34—H34A	120.3
Dy1—C5—H5A	116.8	C35—C34—H34A	120.3
C5—C6—C1	121.1 (2)	C36—C35—C34	120.3 (2)
C5—C6—Dy1	71.38 (14)	C36—C35—H35A	119.8
C1—C6—Dy1	81.43 (14)	C34—C35—H35A	119.8
C5—C6—H6A	119.4	C35—C36—C31	120.9 (2)
C1—C6—H6A	119.4	C35—C36—H36A	119.5
Dy1—C6—H6A	118.5	C31—C36—H36A	119.5
			
C7—N1—C1—C6	3.2 (4)	C13—C14—C15—C16	2.9 (4)
Dy1^i^—N1—C1—C6	−158.01 (18)	C14—C15—C16—C17	−0.1 (4)
C7—N1—C1—C2	−175.7 (2)	C15—C16—C17—C18	−1.7 (4)
Dy1^i^—N1—C1—C2	23.2 (3)	C16—C17—C18—C13	0.6 (4)
C7—N1—C1—Dy1	98.6 (2)	C14—C13—C18—C17	2.2 (4)
Dy1^i^—N1—C1—Dy1	−62.5 (3)	N2—C13—C18—C17	−173.4 (2)
N1—C1—C2—C3	171.6 (2)	C13—N2—C19—C24	−25.1 (3)
C6—C1—C2—C3	−7.3 (3)	Dy1—N2—C19—C24	147.11 (19)
Dy1—C1—C2—C3	−64.1 (2)	C13—N2—C19—C20	157.4 (2)
N1—C1—C2—Dy1	−124.3 (2)	Dy1—N2—C19—C20	−30.4 (3)
C6—C1—C2—Dy1	56.81 (19)	C24—C19—C20—C21	1.8 (3)
C1—C2—C3—C4	4.5 (4)	N2—C19—C20—C21	179.5 (2)
Dy1—C2—C3—C4	−65.8 (2)	C19—C20—C21—C22	−1.8 (4)
C1—C2—C3—Dy1	70.3 (2)	C20—C21—C22—C23	0.0 (4)
C2—C3—C4—C5	−0.6 (4)	C21—C22—C23—C24	1.6 (4)
Dy1—C3—C4—C5	−69.5 (2)	C22—C23—C24—C19	−1.5 (4)
C2—C3—C4—Dy1	68.9 (2)	N2—C19—C24—C23	−177.8 (2)
C3—C4—C5—C6	0.0 (4)	C20—C19—C24—C23	−0.2 (3)
Dy1—C4—C5—C6	−68.3 (2)	C31—N3—C25—C26	164.9 (2)
C3—C4—C5—Dy1	68.3 (2)	Dy1—N3—C25—C26	−16.2 (4)
C4—C5—C6—C1	−3.3 (4)	C31—N3—C25—C30	−18.4 (3)
Dy1—C5—C6—C1	−67.2 (2)	Dy1—N3—C25—C30	160.5 (2)
C4—C5—C6—Dy1	64.0 (2)	N3—C25—C26—C27	−176.6 (2)
N1—C1—C6—C5	−172.2 (2)	C30—C25—C26—C27	6.5 (4)
C2—C1—C6—C5	6.7 (3)	C25—C26—C27—C28	−2.5 (4)
Dy1—C1—C6—C5	62.1 (2)	C26—C27—C28—C29	−3.0 (4)
N1—C1—C6—Dy1	125.7 (2)	C27—C28—C29—C30	4.2 (4)
C2—C1—C6—Dy1	−55.40 (19)	C28—C29—C30—C25	0.0 (4)
C1—N1—C7—C12	115.4 (3)	N3—C25—C30—C29	177.9 (2)
Dy1^i^—N1—C7—C12	−79.6 (2)	C26—C25—C30—C29	−5.3 (4)
C1—N1—C7—C8	−69.5 (3)	C25—N3—C31—C32	126.7 (2)
Dy1^i^—N1—C7—C8	95.5 (2)	Dy1—N3—C31—C32	−52.6 (2)
C12—C7—C8—C9	0.4 (4)	C25—N3—C31—C36	−59.1 (3)
N1—C7—C8—C9	−174.7 (2)	Dy1—N3—C31—C36	121.6 (2)
C7—C8—C9—C10	0.5 (4)	C25—N3—C31—Dy1	179.3 (2)
C8—C9—C10—C11	−0.7 (4)	C36—C31—C32—C33	−1.6 (4)
C9—C10—C11—C12	0.1 (4)	N3—C31—C32—C33	172.8 (2)
C10—C11—C12—C7	0.8 (4)	Dy1—C31—C32—C33	134.2 (2)
C8—C7—C12—C11	−1.1 (4)	C31—C32—C33—C34	1.3 (4)
N1—C7—C12—C11	174.2 (2)	C32—C33—C34—C35	0.4 (4)
C19—N2—C13—C14	105.8 (3)	C33—C34—C35—C36	−1.6 (4)
Dy1—N2—C13—C14	−67.8 (2)	C34—C35—C36—C31	1.2 (4)
C19—N2—C13—C18	−78.7 (3)	C32—C31—C36—C35	0.4 (4)
Dy1—N2—C13—C18	107.7 (2)	N3—C31—C36—C35	−173.8 (2)
C18—C13—C14—C15	−4.0 (4)	Dy1—C31—C36—C35	−109.2 (3)
N2—C13—C14—C15	171.7 (2)		

Symmetry code: (i) −*x*+1, −*y*+1, −*z*+1.

### Bis(µ-diphenylamido-κ2N:N)bis[µ-1κN:2(η6)-diphenylamido]tetrakis(diphenylamido-κN)di-µ3-oxido-tetraerbium(III) benzene disolvate (3-Er) Crystal data

**Table:**

[Er_4_(C_12_H_10_N)_8_O_2_]·2C_6_H_6_	*Z* = 1
*M**_r_* = 2202.93	*F*(000) = 1084
Triclinic, *P*1	*D*_x_ = 1.738 Mg m^−^^3^
*a* = 12.8857 (8) Å	Mo *K*α radiation, λ = 0.71073 Å
*b* = 13.6846 (9) Å	Cell parameters from 8814 reflections
*c* = 13.7411 (9) Å	θ = 2.2–29.1°
α = 61.3447 (8)°	µ = 4.01 mm^−^^1^
β = 82.7796 (10)°	*T* = 88 K
γ = 83.0804 (10)°	Irregular, yellow
*V* = 2104.4 (2) Å^3^	0.35 × 0.28 × 0.11 mm

### Bis(µ-diphenylamido-κ2N:N)bis[µ-1κN:2(η6)-diphenylamido]tetrakis(diphenylamido-κN)di-µ3-oxido-tetraerbium(III) benzene disolvate (3-Er) Data collection

**Table:**

Bruker SMART APEXII CCD diffractometer	9209 reflections with *I* > 2σ(*I*)
Radiation source: fine-focus sealed tube	*R*_int_ = 0.052
φ and ω scans	θ_max_ = 29.2°, θ_min_ = 1.6°
Absorption correction: multi-scan (*TWINABS*; Sheldrick, 2012)	*h* = −17→17
*T*_min_ = 0.254, *T*_max_ = 0.432	*k* = −15→18
51658 measured reflections	*l* = 0→18
10308 independent reflections	

### Bis(µ-diphenylamido-κ2N:N)bis[µ-1κN:2(η6)-diphenylamido]tetrakis(diphenylamido-κN)di-µ3-oxido-tetraerbium(III) benzene disolvate (3-Er) Refinement

**Table:**

Refinement on *F*^2^	Primary atom site location: structure-invariant direct methods
Least-squares matrix: full	Secondary atom site location: difference Fourier map
*R*[*F*^2^ > 2σ(*F*^2^)] = 0.027	Hydrogen site location: inferred from neighbouring sites
*wR*(*F*^2^) = 0.062	H-atom parameters constrained
*S* = 0.96	*w* = 1/[σ^2^(*F*_o_^2^) + (0.0328*P*)^2^] where *P* = (*F*_o_^2^ + 2*F*_c_^2^)/3
10308 reflections	(Δ/σ)_max_ = 0.001
552 parameters	Δρ_max_ = 1.62 e Å^−^^3^
0 restraints	Δρ_min_ = −1.10 e Å^−^^3^

### Bis(µ-diphenylamido-κ2N:N)bis[µ-1κN:2(η6)-diphenylamido]tetrakis(diphenylamido-κN)di-µ3-oxido-tetraerbium(III) benzene disolvate (3-Er) Special details

**Table:**

Geometry. All esds (except the esd in the dihedral angle between two l.s. planes) are estimated using the full covariance matrix. The cell esds are taken into account individually in the estimation of esds in distances, angles and torsion angles; correlations between esds in cell parameters are only used when they are defined by crystal symmetry. An approximate (isotropic) treatment of cell esds is used for estimating esds involving l.s. planes.
Refinement. A yellow crystal of approximate dimensions 0.106 x 0.278 x 0.347 mm was mounted on a glass fiber and transferred to a Bruker SMART APEX II diffractometer. The APEX2 program package and the CELL_NOW were used to determine the unit-cell parameters. Data was collected using a 10 sec/frame scan time for a sphere of diffraction data. The raw frame data was processed using SAINT and TWINABS to yield the reflection data file (HKLF5 format). Subsequent calculations were carried out using the SHELXTL program. There were no systematic absences nor any diffraction symmetry other than the Friedel condition The centrosymmetric triclinic space group P-1 was assigned and later determined to be correct. The structure was solved by direct methods and refined on F2 by full-matrix least-squares techniques. The analytical scattering factors for neutral atoms were used throughout the analysis. Hydrogen atoms were included using a riding model. The molecule was located about an inversion center. There were two molecules of benzene solvent present per empirical formula-unit. At convergence, wR2 = 0.0624 and Goof = 0.96 for 552 variables refined against 10308 data (0.73Å), R1 = 0.0273 for those 9209 with I > 2.0sigma(I). The structure was refined as a three-component twin

### Bis(µ-diphenylamido-κ2N:N)bis[µ-1κN:2(η6)-diphenylamido]tetrakis(diphenylamido-κN)di-µ3-oxido-tetraerbium(III) benzene disolvate (3-Er) Fractional atomic coordinates and isotropic or equivalent isotropic displacement parameters (Å^2^)

**Table:**

	*x*	*y*	*z*	*U*_iso_*/*U*_eq_	
Er1	0.71708 (2)	0.58391 (2)	0.33190 (2)	0.01164 (5)	
Er2	0.56207 (2)	0.40689 (2)	0.61938 (2)	0.01102 (5)	
O1	0.5913 (2)	0.4965 (2)	0.4381 (2)	0.0130 (6)	
N1	0.6896 (3)	0.5078 (3)	0.6255 (3)	0.0150 (7)	
N2	0.5780 (3)	0.7048 (3)	0.2301 (3)	0.0129 (7)	
N3	0.7699 (3)	0.4884 (3)	0.2397 (3)	0.0179 (7)	
N4	0.6547 (3)	0.2348 (3)	0.6816 (3)	0.0138 (7)	
C1	0.7748 (3)	0.5260 (3)	0.5492 (3)	0.0145 (8)	
C2	0.8211 (3)	0.6295 (4)	0.4871 (4)	0.0179 (9)	
H2A	0.7989	0.6882	0.5049	0.021*	
C3	0.8985 (3)	0.6476 (4)	0.4003 (4)	0.0217 (10)	
H3A	0.9267	0.7186	0.3595	0.026*	
C4	0.9348 (3)	0.5633 (4)	0.3728 (4)	0.0190 (9)	
H4A	0.9857	0.5767	0.3123	0.023*	
C5	0.8946 (3)	0.4586 (4)	0.4364 (4)	0.0171 (9)	
H5A	0.9214	0.3993	0.4210	0.021*	
C6	0.8166 (3)	0.4392 (4)	0.5213 (3)	0.0149 (9)	
H6A	0.7902	0.3673	0.5620	0.018*	
C7	0.6791 (3)	0.5710 (3)	0.6850 (3)	0.0167 (9)	
C8	0.7583 (3)	0.5611 (4)	0.7512 (4)	0.0203 (9)	
H8A	0.8216	0.5177	0.7512	0.024*	
C9	0.7450 (4)	0.6141 (4)	0.8168 (4)	0.0262 (10)	
H9A	0.7989	0.6065	0.8617	0.031*	
C10	0.6529 (4)	0.6782 (4)	0.8166 (4)	0.0298 (11)	
H10A	0.6436	0.7142	0.8617	0.036*	
C11	0.5748 (4)	0.6898 (4)	0.7509 (4)	0.0254 (11)	
H11A	0.5118	0.7337	0.7509	0.030*	
C12	0.5885 (4)	0.6368 (4)	0.6845 (4)	0.0205 (9)	
H12A	0.5350	0.6461	0.6385	0.025*	
C13	0.6320 (3)	0.8012 (3)	0.1977 (4)	0.0147 (9)	
C14	0.6592 (3)	0.8810 (4)	0.0865 (4)	0.0173 (9)	
H14A	0.6302	0.8789	0.0273	0.021*	
C15	0.7272 (4)	0.9618 (4)	0.0627 (4)	0.0224 (10)	
H15A	0.7438	1.0146	−0.0126	0.027*	
C16	0.7714 (4)	0.9671 (4)	0.1468 (4)	0.0238 (11)	
H16A	0.8185	1.0226	0.1296	0.029*	
C17	0.7457 (4)	0.8899 (4)	0.2568 (4)	0.0208 (10)	
H17A	0.7755	0.8924	0.3154	0.025*	
C18	0.6762 (3)	0.8086 (3)	0.2813 (4)	0.0154 (9)	
H18A	0.6586	0.7572	0.3568	0.018*	
C19	0.5232 (3)	0.6974 (4)	0.1504 (3)	0.0140 (8)	
C20	0.5248 (4)	0.5954 (4)	0.1537 (4)	0.0202 (9)	
H20A	0.5633	0.5331	0.2073	0.024*	
C21	0.4706 (4)	0.5814 (4)	0.0793 (4)	0.0249 (10)	
H21A	0.4722	0.5101	0.0830	0.030*	
C22	0.4153 (4)	0.6706 (4)	0.0016 (4)	0.0246 (10)	
H22A	0.3810	0.6620	−0.0509	0.030*	
C23	0.4095 (3)	0.7730 (4)	−0.0007 (4)	0.0229 (10)	
H23A	0.3696	0.8342	−0.0537	0.028*	
C24	0.4611 (3)	0.7876 (4)	0.0734 (4)	0.0197 (9)	
H24A	0.4549	0.8579	0.0727	0.024*	
C25	0.8398 (3)	0.5580 (4)	0.1525 (4)	0.0178 (9)	
C26	0.8081 (4)	0.6696 (4)	0.0868 (4)	0.0218 (10)	
H26A	0.7373	0.6946	0.0962	0.026*	
C27	0.8765 (4)	0.7462 (4)	0.0078 (4)	0.0299 (11)	
H27A	0.8532	0.8224	−0.0346	0.036*	
C28	0.9786 (4)	0.7094 (4)	−0.0078 (4)	0.0298 (11)	
H28A	1.0263	0.7601	−0.0617	0.036*	
C29	1.0112 (4)	0.5979 (4)	0.0555 (4)	0.0271 (11)	
H29A	1.0811	0.5726	0.0430	0.033*	
C30	0.9446 (3)	0.5233 (4)	0.1360 (4)	0.0224 (10)	
H30A	0.9696	0.4481	0.1805	0.027*	
C31	0.7641 (3)	0.3809 (4)	0.2550 (4)	0.0194 (9)	
C32	0.7791 (3)	0.3575 (4)	0.1648 (4)	0.0242 (10)	
H32A	0.7946	0.4156	0.0917	0.029*	
C33	0.7714 (4)	0.2495 (5)	0.1822 (5)	0.0279 (11)	
H33A	0.7838	0.2344	0.1209	0.033*	
C34	0.7463 (4)	0.1649 (4)	0.2860 (5)	0.0295 (12)	
H34A	0.7407	0.0918	0.2967	0.035*	
C35	0.7291 (4)	0.1870 (4)	0.3758 (5)	0.0298 (11)	
H35A	0.7110	0.1288	0.4481	0.036*	
C36	0.7382 (3)	0.2932 (4)	0.3602 (4)	0.0234 (10)	
H36A	0.7267	0.3069	0.4224	0.028*	
C37	0.6008 (3)	0.1578 (4)	0.6696 (3)	0.0155 (9)	
C38	0.5506 (3)	0.1928 (4)	0.5726 (3)	0.0178 (9)	
H38A	0.5553	0.2678	0.5155	0.021*	
C39	0.4943 (4)	0.1223 (4)	0.5565 (4)	0.0225 (10)	
H39A	0.4616	0.1489	0.4892	0.027*	
C40	0.4859 (4)	0.0141 (4)	0.6380 (4)	0.0230 (10)	
H40A	0.4477	−0.0348	0.6274	0.028*	
C41	0.5337 (3)	−0.0241 (4)	0.7369 (4)	0.0233 (10)	
H41A	0.5276	−0.0990	0.7936	0.028*	
C42	0.5899 (3)	0.0469 (4)	0.7526 (4)	0.0186 (9)	
H42A	0.6216	0.0201	0.8205	0.022*	
C43	0.7467 (3)	0.1943 (4)	0.7385 (3)	0.0167 (9)	
C44	0.8148 (3)	0.1077 (4)	0.7362 (4)	0.0207 (10)	
H44A	0.7968	0.0706	0.6979	0.025*	
C45	0.9077 (4)	0.0742 (4)	0.7877 (4)	0.0285 (11)	
H45A	0.9517	0.0144	0.7852	0.034*	
C46	0.9372 (4)	0.1280 (4)	0.8436 (4)	0.0310 (12)	
H46A	1.0007	0.1051	0.8795	0.037*	
C47	0.8718 (4)	0.2152 (4)	0.8456 (4)	0.0266 (11)	
H47A	0.8913	0.2534	0.8822	0.032*	
C48	0.7779 (3)	0.2476 (4)	0.7946 (4)	0.0211 (10)	
H48A	0.7339	0.3071	0.7977	0.025*	
C49	1.0007 (4)	0.8477 (5)	0.5371 (5)	0.0363 (13)	
H49A	1.0683	0.8361	0.5061	0.044*	
C50	0.9877 (4)	0.8289 (5)	0.6451 (5)	0.0380 (13)	
H50A	1.0458	0.8023	0.6895	0.046*	
C51	0.8906 (5)	0.8483 (5)	0.6894 (5)	0.0424 (14)	
H51A	0.8823	0.8369	0.7637	0.051*	
C52	0.8041 (4)	0.8847 (5)	0.6255 (5)	0.0365 (13)	
H52A	0.7368	0.8977	0.6560	0.044*	
C53	0.8177 (4)	0.9014 (4)	0.5189 (5)	0.0326 (12)	
H53A	0.7592	0.9254	0.4752	0.039*	
C54	0.9157 (4)	0.8838 (5)	0.4732 (5)	0.0329 (12)	
H54A	0.9243	0.8963	0.3985	0.039*	

### Bis(µ-diphenylamido-κ2N:N)bis[µ-1κN:2(η6)-diphenylamido]tetrakis(diphenylamido-κN)di-µ3-oxido-tetraerbium(III) benzene disolvate (3-Er) Atomic displacement parameters (Å^2^)

**Table:**

	*U*^11^	*U*^22^	*U*^33^	*U*^12^	*U*^13^	*U*^23^
Er1	0.01054 (9)	0.01134 (10)	0.01106 (9)	−0.00114 (7)	−0.00026 (7)	−0.00374 (7)
Er2	0.01002 (9)	0.01011 (10)	0.01026 (8)	−0.00106 (7)	−0.00127 (7)	−0.00248 (7)
O1	0.0114 (14)	0.0144 (15)	0.0126 (13)	−0.0020 (11)	0.0011 (11)	−0.0060 (11)
N1	0.0160 (18)	0.0145 (18)	0.0150 (16)	−0.0019 (14)	−0.0014 (14)	−0.0072 (14)
N2	0.0142 (17)	0.0123 (17)	0.0125 (16)	−0.0025 (14)	−0.0005 (13)	−0.0059 (14)
N3	0.0184 (18)	0.0159 (19)	0.0183 (17)	−0.0009 (15)	−0.0016 (15)	−0.0072 (15)
N4	0.0132 (17)	0.0133 (18)	0.0149 (16)	−0.0011 (14)	−0.0026 (13)	−0.0063 (14)
C1	0.014 (2)	0.012 (2)	0.0132 (18)	−0.0010 (16)	−0.0058 (16)	−0.0014 (16)
C2	0.016 (2)	0.017 (2)	0.021 (2)	−0.0027 (17)	−0.0060 (17)	−0.0075 (18)
C3	0.015 (2)	0.022 (3)	0.022 (2)	−0.0070 (18)	−0.0036 (18)	−0.0042 (18)
C4	0.012 (2)	0.025 (2)	0.016 (2)	−0.0036 (17)	−0.0029 (16)	−0.0057 (18)
C5	0.016 (2)	0.018 (2)	0.0156 (19)	0.0047 (17)	−0.0063 (17)	−0.0064 (17)
C6	0.012 (2)	0.017 (2)	0.0135 (18)	−0.0032 (16)	−0.0037 (15)	−0.0043 (16)
C7	0.021 (2)	0.012 (2)	0.0162 (19)	−0.0026 (17)	−0.0014 (17)	−0.0054 (16)
C8	0.021 (2)	0.018 (2)	0.022 (2)	−0.0006 (18)	−0.0034 (18)	−0.0088 (18)
C9	0.028 (2)	0.027 (3)	0.028 (2)	−0.003 (2)	−0.008 (2)	−0.015 (2)
C10	0.045 (3)	0.025 (3)	0.027 (2)	−0.001 (2)	−0.004 (2)	−0.018 (2)
C11	0.028 (3)	0.020 (2)	0.030 (2)	0.002 (2)	−0.004 (2)	−0.014 (2)
C12	0.023 (2)	0.019 (2)	0.018 (2)	−0.0013 (18)	−0.0055 (18)	−0.0068 (18)
C13	0.010 (2)	0.011 (2)	0.018 (2)	0.0024 (15)	−0.0012 (16)	−0.0035 (16)
C14	0.019 (2)	0.015 (2)	0.019 (2)	−0.0018 (17)	−0.0008 (17)	−0.0081 (17)
C15	0.023 (2)	0.012 (2)	0.026 (2)	−0.0031 (18)	0.0059 (19)	−0.0053 (18)
C16	0.019 (2)	0.016 (2)	0.039 (3)	−0.0037 (18)	0.000 (2)	−0.015 (2)
C17	0.021 (2)	0.018 (2)	0.029 (2)	0.0010 (18)	−0.0042 (19)	−0.016 (2)
C18	0.015 (2)	0.012 (2)	0.018 (2)	0.0008 (16)	−0.0039 (16)	−0.0058 (16)
C19	0.0080 (19)	0.018 (2)	0.0136 (18)	−0.0033 (16)	0.0017 (15)	−0.0053 (16)
C20	0.024 (2)	0.017 (2)	0.018 (2)	−0.0017 (18)	−0.0071 (18)	−0.0058 (18)
C21	0.034 (3)	0.022 (2)	0.023 (2)	−0.005 (2)	−0.007 (2)	−0.012 (2)
C22	0.023 (2)	0.034 (3)	0.019 (2)	0.000 (2)	−0.0063 (18)	−0.014 (2)
C23	0.021 (2)	0.027 (3)	0.016 (2)	0.0011 (19)	−0.0050 (18)	−0.0067 (19)
C24	0.019 (2)	0.018 (2)	0.020 (2)	0.0005 (18)	−0.0010 (17)	−0.0070 (18)
C25	0.021 (2)	0.018 (2)	0.019 (2)	−0.0015 (18)	−0.0006 (17)	−0.0119 (18)
C26	0.018 (2)	0.024 (3)	0.024 (2)	0.0005 (19)	0.0035 (18)	−0.013 (2)
C27	0.027 (3)	0.028 (3)	0.027 (3)	−0.007 (2)	0.008 (2)	−0.009 (2)
C28	0.029 (3)	0.032 (3)	0.030 (3)	−0.011 (2)	0.006 (2)	−0.016 (2)
C29	0.020 (2)	0.039 (3)	0.028 (2)	−0.002 (2)	0.003 (2)	−0.020 (2)
C30	0.017 (2)	0.028 (3)	0.023 (2)	−0.0006 (19)	−0.0032 (18)	−0.013 (2)
C31	0.009 (2)	0.026 (2)	0.028 (2)	0.0013 (17)	0.0000 (17)	−0.018 (2)
C32	0.019 (2)	0.029 (3)	0.030 (2)	−0.003 (2)	−0.0010 (19)	−0.018 (2)
C33	0.017 (2)	0.041 (3)	0.042 (3)	0.001 (2)	−0.006 (2)	−0.033 (3)
C34	0.020 (2)	0.028 (3)	0.052 (3)	−0.004 (2)	0.005 (2)	−0.028 (3)
C35	0.024 (2)	0.023 (3)	0.042 (3)	−0.005 (2)	0.004 (2)	−0.016 (2)
C36	0.018 (2)	0.026 (3)	0.031 (3)	−0.0043 (19)	0.0055 (19)	−0.019 (2)
C37	0.014 (2)	0.017 (2)	0.0130 (18)	0.0007 (17)	0.0023 (16)	−0.0063 (17)
C38	0.019 (2)	0.015 (2)	0.0162 (19)	−0.0011 (17)	0.0018 (17)	−0.0049 (17)
C39	0.021 (2)	0.027 (3)	0.024 (2)	−0.0035 (19)	0.0029 (19)	−0.016 (2)
C40	0.025 (2)	0.022 (2)	0.029 (2)	−0.0068 (19)	0.003 (2)	−0.018 (2)
C41	0.028 (2)	0.014 (2)	0.025 (2)	−0.0029 (19)	0.007 (2)	−0.0091 (19)
C42	0.019 (2)	0.018 (2)	0.017 (2)	0.0001 (17)	−0.0018 (17)	−0.0072 (18)
C43	0.011 (2)	0.015 (2)	0.0128 (18)	0.0003 (16)	−0.0007 (15)	0.0014 (16)
C44	0.017 (2)	0.021 (2)	0.019 (2)	0.0022 (18)	−0.0004 (17)	−0.0063 (18)
C45	0.018 (2)	0.027 (3)	0.026 (2)	0.002 (2)	0.0027 (19)	−0.003 (2)
C46	0.013 (2)	0.035 (3)	0.025 (2)	−0.001 (2)	−0.0031 (19)	0.001 (2)
C47	0.022 (2)	0.028 (3)	0.020 (2)	−0.011 (2)	−0.0058 (19)	−0.0003 (19)
C48	0.017 (2)	0.016 (2)	0.019 (2)	−0.0024 (18)	−0.0006 (17)	−0.0003 (17)
C49	0.027 (3)	0.038 (3)	0.047 (3)	−0.004 (2)	−0.001 (2)	−0.022 (3)
C50	0.033 (3)	0.037 (3)	0.039 (3)	−0.005 (2)	−0.007 (2)	−0.012 (3)
C51	0.050 (4)	0.049 (4)	0.029 (3)	−0.010 (3)	−0.002 (3)	−0.017 (3)
C52	0.033 (3)	0.036 (3)	0.036 (3)	−0.005 (2)	0.003 (2)	−0.014 (2)
C53	0.032 (3)	0.031 (3)	0.036 (3)	−0.003 (2)	−0.007 (2)	−0.016 (2)
C54	0.034 (3)	0.038 (3)	0.031 (3)	−0.006 (2)	0.001 (2)	−0.019 (2)

### Bis(µ-diphenylamido-κ2N:N)bis[µ-1κN:2(η6)-diphenylamido]tetrakis(diphenylamido-κN)di-µ3-oxido-tetraerbium(III) benzene disolvate (3-Er) Geometric parameters (Å, º)

**Table:**

Er1—Cnt	2.516	C19—Er2^i^	2.903 (4)
Er1—O1	2.095 (3)	C20—C21	1.403 (6)
Er1—N3	2.222 (3)	C20—H20A	0.9500
Er1—N2	2.367 (4)	C21—C22	1.367 (7)
Er1—C6	2.761 (4)	C21—H21A	0.9500
Er1—C5	2.784 (4)	C22—C23	1.380 (7)
Er1—C18	2.805 (4)	C22—H22A	0.9500
Er1—C13	2.812 (4)	C23—C24	1.385 (6)
Er1—C1	2.871 (4)	C23—H23A	0.9500
Er1—C4	2.884 (4)	C24—H24A	0.9500
Er1—C25	2.904 (4)	C25—C26	1.392 (6)
Er1—C2	2.988 (4)	C25—C30	1.404 (6)
Er1—C3	2.989 (4)	C26—C27	1.394 (6)
Er2—O1	2.190 (3)	C26—H26A	0.9500
Er2—O1^i^	2.245 (3)	C27—C28	1.379 (7)
Er2—N1	2.303 (3)	C27—H27A	0.9500
Er2—N4	2.313 (4)	C28—C29	1.387 (7)
Er2—N2^i^	2.563 (3)	C28—H28A	0.9500
Er2—C19^i^	2.903 (4)	C29—C30	1.375 (6)
Er2—Er2^i^	3.4836 (4)	C29—H29A	0.9500
Er2—Er1^i^	3.5734 (3)	C30—H30A	0.9500
O1—Er2^i^	2.245 (3)	C31—C36	1.398 (7)
N1—C1	1.376 (5)	C31—C32	1.408 (6)
N1—C7	1.434 (5)	C32—C33	1.393 (7)
N2—C13	1.415 (5)	C32—H32A	0.9500
N2—C19	1.425 (5)	C33—C34	1.368 (8)
N2—Er2^i^	2.563 (3)	C33—H33A	0.9500
N3—C31	1.393 (6)	C34—C35	1.391 (7)
N3—C25	1.412 (5)	C34—H34A	0.9500
N4—C43	1.408 (5)	C35—C36	1.381 (7)
N4—C37	1.411 (5)	C35—H35A	0.9500
C1—C2	1.417 (6)	C36—H36A	0.9500
C1—C6	1.442 (6)	C37—C38	1.397 (6)
C2—C3	1.398 (6)	C37—C42	1.405 (6)
C2—H2A	0.9500	C38—C39	1.386 (6)
C3—C4	1.391 (6)	C38—H38A	0.9500
C3—H3A	0.9500	C39—C40	1.370 (7)
C4—C5	1.395 (6)	C39—H39A	0.9500
C4—H4A	0.9500	C40—C41	1.396 (7)
C5—C6	1.383 (6)	C40—H40A	0.9500
C5—H5A	0.9500	C41—C42	1.388 (6)
C6—H6A	0.9500	C41—H41A	0.9500
C7—C12	1.385 (6)	C42—H42A	0.9500
C7—C8	1.403 (6)	C43—C44	1.398 (6)
C8—C9	1.387 (6)	C43—C48	1.406 (7)
C8—H8A	0.9500	C44—C45	1.383 (6)
C9—C10	1.389 (7)	C44—H44A	0.9500
C9—H9A	0.9500	C45—C46	1.399 (8)
C10—C11	1.380 (7)	C45—H45A	0.9500
C10—H10A	0.9500	C46—C47	1.385 (7)
C11—C12	1.397 (6)	C46—H46A	0.9500
C11—H11A	0.9500	C47—C48	1.393 (6)
C12—H12A	0.9500	C47—H47A	0.9500
C13—C18	1.395 (6)	C48—H48A	0.9500
C13—C14	1.416 (6)	C49—C50	1.371 (8)
C14—C15	1.383 (6)	C49—C54	1.383 (8)
C14—H14A	0.9500	C49—H49A	0.9500
C15—C16	1.386 (7)	C50—C51	1.374 (8)
C15—H15A	0.9500	C50—H50A	0.9500
C16—C17	1.392 (7)	C51—C52	1.398 (8)
C16—H16A	0.9500	C51—H51A	0.9500
C17—C18	1.400 (6)	C52—C53	1.362 (7)
C17—H17A	0.9500	C52—H52A	0.9500
C18—H18A	0.9500	C53—C54	1.384 (7)
C19—C20	1.372 (6)	C53—H53A	0.9500
C19—C24	1.416 (6)	C54—H54A	0.9500
			
Cnt—Er1—O1	103.8	Er1—C6—H6A	115.2
Cnt—Er1—N1	148.5	C12—C7—C8	118.6 (4)
Cnt—Er1—N2	106.2	C12—C7—N1	121.1 (4)
O1—Er1—N3	102.87 (12)	C8—C7—N1	120.2 (4)
O1—Er1—N2	81.47 (11)	C9—C8—C7	120.7 (4)
N3—Er1—N2	102.68 (13)	C9—C8—H8A	119.7
O1—Er1—C6	78.87 (11)	C7—C8—H8A	119.7
N3—Er1—C6	95.72 (13)	C8—C9—C10	119.9 (4)
N2—Er1—C6	155.59 (12)	C8—C9—H9A	120.1
O1—Er1—C5	104.41 (12)	C10—C9—H9A	120.1
N3—Er1—C5	77.15 (13)	C11—C10—C9	120.1 (5)
N2—Er1—C5	174.04 (12)	C11—C10—H10A	119.9
C6—Er1—C5	28.87 (12)	C9—C10—H10A	119.9
O1—Er1—C18	107.51 (12)	C10—C11—C12	119.9 (5)
N3—Er1—C18	137.18 (13)	C10—C11—H11A	120.0
N2—Er1—C18	54.34 (12)	C12—C11—H11A	120.0
C6—Er1—C18	119.04 (13)	C7—C12—C11	120.8 (4)
C5—Er1—C18	121.87 (13)	C7—C12—H12A	119.6
O1—Er1—C13	105.59 (11)	C11—C12—H12A	119.6
N3—Er1—C13	113.71 (13)	C18—C13—N2	116.8 (4)
N2—Er1—C13	30.18 (11)	C18—C13—C14	117.1 (4)
C6—Er1—C13	147.75 (13)	N2—C13—C14	125.3 (4)
C5—Er1—C13	144.46 (12)	C18—C13—Er1	75.3 (2)
C18—Er1—C13	28.76 (12)	N2—C13—Er1	57.2 (2)
O1—Er1—C1	76.44 (11)	C14—C13—Er1	131.7 (3)
N3—Er1—C1	125.21 (13)	C15—C14—C13	121.1 (4)
N2—Er1—C1	130.40 (12)	C15—C14—H14A	119.5
C6—Er1—C1	29.58 (12)	C13—C14—H14A	119.5
C5—Er1—C1	51.58 (12)	C14—C15—C16	121.1 (4)
C18—Er1—C1	91.24 (12)	C14—C15—H15A	119.4
C13—Er1—C1	119.16 (12)	C16—C15—H15A	119.4
O1—Er1—C4	129.68 (12)	C15—C16—C17	118.9 (4)
N3—Er1—C4	85.36 (13)	C15—C16—H16A	120.5
N2—Er1—C4	145.78 (12)	C17—C16—H16A	120.5
C6—Er1—C4	50.82 (12)	C16—C17—C18	120.1 (4)
C5—Er1—C4	28.43 (12)	C16—C17—H17A	120.0
C18—Er1—C4	97.37 (13)	C18—C17—H17A	120.0
C13—Er1—C4	116.22 (12)	C13—C18—C17	121.7 (4)
C1—Er1—C4	59.66 (12)	C13—C18—Er1	75.9 (2)
O1—Er1—C25	130.35 (12)	C17—C18—Er1	129.9 (3)
N3—Er1—C25	28.19 (12)	C13—C18—H18A	119.2
N2—Er1—C25	98.55 (12)	C17—C18—H18A	119.2
C6—Er1—C25	105.17 (13)	Er1—C18—H18A	65.9
C5—Er1—C25	78.44 (13)	C20—C19—C24	118.4 (4)
C18—Er1—C25	112.34 (12)	C20—C19—N2	118.0 (4)
C13—Er1—C25	96.02 (12)	C24—C19—N2	123.4 (4)
C1—Er1—C25	129.44 (12)	C20—C19—Er2^i^	87.9 (3)
C4—Er1—C25	72.95 (12)	C24—C19—Er2^i^	116.4 (3)
O1—Er1—C2	100.08 (11)	N2—C19—Er2^i^	61.94 (19)
N3—Er1—C2	132.84 (13)	C19—C20—C21	121.1 (4)
N2—Er1—C2	121.19 (12)	C19—C20—H20A	119.4
C6—Er1—C2	49.61 (12)	C21—C20—H20A	119.4
C5—Er1—C2	57.37 (13)	C22—C21—C20	119.9 (5)
C18—Er1—C2	70.02 (12)	C22—C21—H21A	120.1
C13—Er1—C2	98.66 (12)	C20—C21—H21A	120.1
C1—Er1—C2	27.89 (11)	C21—C22—C23	120.0 (4)
C4—Er1—C2	48.81 (12)	C21—C22—H22A	120.0
C25—Er1—C2	120.41 (12)	C23—C22—H22A	120.0
O1—Er1—C3	125.75 (12)	C22—C23—C24	120.8 (4)
N3—Er1—C3	111.63 (13)	C22—C23—H23A	119.6
N2—Er1—C3	126.87 (12)	C24—C23—H23A	119.6
C6—Er1—C3	57.72 (12)	C23—C24—C19	119.7 (4)
C5—Er1—C3	48.83 (13)	C23—C24—H24A	120.2
C18—Er1—C3	73.12 (12)	C19—C24—H24A	120.2
C13—Er1—C3	97.40 (12)	C26—C25—C30	117.5 (4)
C1—Er1—C3	49.59 (12)	C26—C25—N3	119.1 (4)
C4—Er1—C3	27.32 (12)	C30—C25—N3	123.0 (4)
C25—Er1—C3	93.91 (12)	C26—C25—Er1	84.9 (3)
C2—Er1—C3	27.06 (11)	C30—C25—Er1	134.4 (3)
O1—Er2—O1^i^	76.46 (11)	N3—C25—Er1	48.0 (2)
O1—Er2—N1	86.63 (11)	C25—C26—C27	122.3 (4)
O1^i^—Er2—N1	115.28 (12)	C25—C26—H26A	118.8
O1—Er2—N4	107.41 (11)	C27—C26—H26A	118.8
O1^i^—Er2—N4	145.79 (11)	C28—C27—C26	118.9 (5)
N1—Er2—N4	98.93 (12)	C28—C27—H27A	120.6
O1—Er2—N2^i^	140.60 (11)	C26—C27—H27A	120.6
O1^i^—Er2—N2^i^	74.44 (10)	C27—C28—C29	119.6 (5)
N1—Er2—N2^i^	129.93 (12)	C27—C28—H28A	120.2
N4—Er2—N2^i^	83.60 (12)	C29—C28—H28A	120.2
O1—Er2—C19^i^	167.75 (11)	C30—C29—C28	121.4 (5)
O1^i^—Er2—C19^i^	91.60 (11)	C30—C29—H29A	119.3
N1—Er2—C19^i^	101.16 (12)	C28—C29—H29A	119.3
N4—Er2—C19^i^	80.92 (12)	C29—C30—C25	120.2 (5)
N2^i^—Er2—C19^i^	29.38 (11)	C29—C30—H30A	119.9
O1—Er2—Er2^i^	38.79 (7)	C25—C30—H30A	119.9
O1^i^—Er2—Er2^i^	37.67 (7)	N3—C31—C36	121.0 (4)
N1—Er2—Er2^i^	103.78 (9)	N3—C31—C32	121.5 (4)
N4—Er2—Er2^i^	136.14 (9)	C36—C31—C32	117.5 (4)
N2^i^—Er2—Er2^i^	108.23 (8)	C33—C32—C31	120.3 (5)
C19^i^—Er2—Er2^i^	129.22 (8)	C33—C32—H32A	119.8
O1—Er2—Er1^i^	104.75 (7)	C31—C32—H32A	119.8
O1^i^—Er2—Er1^i^	33.22 (7)	C34—C33—C32	121.1 (5)
N1—Er2—Er1^i^	133.86 (9)	C34—C33—H33A	119.5
N4—Er2—Er1^i^	118.69 (8)	C32—C33—H33A	119.5
N2^i^—Er2—Er1^i^	41.42 (8)	C33—C34—C35	119.4 (5)
C19^i^—Er2—Er1^i^	63.05 (8)	C33—C34—H34A	120.3
Er2^i^—Er2—Er1^i^	67.723 (6)	C35—C34—H34A	120.3
O1—Er2—Er1	22.83 (7)	C36—C35—C34	120.2 (5)
O1^i^—Er2—Er1	93.38 (7)	C36—C35—H35A	119.9
N1—Er2—Er1	65.26 (9)	C34—C35—H35A	119.9
N4—Er2—Er1	101.68 (8)	C35—C36—C31	121.5 (5)
N2^i^—Er2—Er1	163.43 (8)	C35—C36—H36A	119.3
C19^i^—Er2—Er1	166.36 (8)	C31—C36—H36A	119.3
Er2^i^—Er2—Er1	57.225 (7)	C38—C37—C42	116.8 (4)
Er1^i^—Er2—Er1	124.949 (6)	C38—C37—N4	119.5 (4)
Er1—O1—Er2	133.25 (14)	C42—C37—N4	123.6 (4)
Er1—O1—Er2^i^	110.82 (12)	C39—C38—C37	122.5 (4)
Er2—O1—Er2^i^	103.54 (11)	C39—C38—H38A	118.8
C1—N1—C7	116.7 (3)	C37—C38—H38A	118.8
C1—N1—Er2	116.2 (3)	C40—C39—C38	119.7 (5)
C7—N1—Er2	125.9 (3)	C40—C39—H39A	120.1
C13—N2—C19	119.3 (3)	C38—C39—H39A	120.1
C13—N2—Er1	92.6 (2)	C39—C40—C41	119.8 (4)
C19—N2—Er1	128.1 (3)	C39—C40—H40A	120.1
C13—N2—Er2^i^	138.0 (3)	C41—C40—H40A	120.1
C19—N2—Er2^i^	88.7 (2)	C42—C41—C40	120.3 (4)
Er1—N2—Er2^i^	92.84 (11)	C42—C41—H41A	119.9
C31—N3—C25	117.6 (4)	C40—C41—H41A	119.9
C31—N3—Er1	137.7 (3)	C41—C42—C37	121.0 (4)
C25—N3—Er1	103.8 (3)	C41—C42—H42A	119.5
C43—N4—C37	117.5 (4)	C37—C42—H42A	119.5
C43—N4—Er2	130.3 (3)	C44—C43—C48	116.7 (4)
C37—N4—Er2	111.8 (3)	C44—C43—N4	123.6 (4)
N1—C1—C2	124.2 (4)	C48—C43—N4	119.5 (4)
N1—C1—C6	119.8 (4)	C45—C44—C43	122.2 (5)
C2—C1—C6	115.7 (4)	C45—C44—H44A	118.9
N1—C1—Er1	112.7 (3)	C43—C44—H44A	118.9
C2—C1—Er1	80.6 (2)	C44—C45—C46	120.3 (5)
C6—C1—Er1	71.0 (2)	C44—C45—H45A	119.8
C3—C2—C1	121.8 (4)	C46—C45—H45A	119.8
C3—C2—Er1	76.5 (3)	C47—C46—C45	118.7 (5)
C1—C2—Er1	71.5 (2)	C47—C46—H46A	120.7
C3—C2—H2A	119.1	C45—C46—H46A	120.7
C1—C2—H2A	119.1	C46—C47—C48	120.7 (5)
Er1—C2—H2A	124.5	C46—C47—H47A	119.7
C4—C3—C2	121.0 (4)	C48—C47—H47A	119.7
C4—C3—Er1	72.1 (2)	C47—C48—C43	121.5 (5)
C2—C3—Er1	76.4 (2)	C47—C48—H48A	119.3
C4—C3—H3A	119.5	C43—C48—H48A	119.3
C2—C3—H3A	119.5	C50—C49—C54	120.0 (5)
Er1—C3—H3A	123.4	C50—C49—H49A	120.0
C3—C4—C5	118.4 (4)	C54—C49—H49A	120.0
C3—C4—Er1	80.6 (2)	C49—C50—C51	120.2 (6)
C5—C4—Er1	71.8 (2)	C49—C50—H50A	119.9
C3—C4—H4A	120.8	C51—C50—H50A	119.9
C5—C4—H4A	120.8	C50—C51—C52	120.3 (5)
Er1—C4—H4A	117.7	C50—C51—H51A	119.9
C6—C5—C4	121.6 (4)	C52—C51—H51A	119.9
C6—C5—Er1	74.6 (2)	C53—C52—C51	119.0 (5)
C4—C5—Er1	79.8 (3)	C53—C52—H52A	120.5
C6—C5—H5A	119.2	C51—C52—H52A	120.5
C4—C5—H5A	119.2	C52—C53—C54	121.0 (5)
Er1—C5—H5A	117.0	C52—C53—H53A	119.5
C5—C6—C1	121.3 (4)	C54—C53—H53A	119.5
C5—C6—Er1	76.5 (2)	C49—C54—C53	119.6 (5)
C1—C6—Er1	79.4 (2)	C49—C54—H54A	120.2
C5—C6—H6A	119.4	C53—C54—H54A	120.2
C1—C6—H6A	119.4		
			
C7—N1—C1—C2	−31.7 (6)	Er1—N2—C19—Er2^i^	92.6 (2)
Er2—N1—C1—C2	136.5 (4)	C24—C19—C20—C21	3.1 (6)
C7—N1—C1—C6	153.8 (4)	N2—C19—C20—C21	178.6 (4)
Er2—N1—C1—C6	−38.0 (5)	Er2^i^—C19—C20—C21	122.1 (4)
C7—N1—C1—Er1	−125.8 (3)	C19—C20—C21—C22	0.3 (7)
Er2—N1—C1—Er1	42.4 (3)	C20—C21—C22—C23	−2.6 (7)
N1—C1—C2—C3	−171.4 (4)	C21—C22—C23—C24	1.5 (7)
C6—C1—C2—C3	3.3 (6)	C22—C23—C24—C19	1.9 (6)
Er1—C1—C2—C3	−60.2 (4)	C20—C19—C24—C23	−4.1 (6)
N1—C1—C2—Er1	−111.2 (4)	N2—C19—C24—C23	−179.4 (4)
C6—C1—C2—Er1	63.5 (3)	Er2^i^—C19—C24—C23	−106.9 (4)
C1—C2—C3—C4	−1.3 (7)	C31—N3—C25—C26	138.6 (4)
Er1—C2—C3—C4	−59.1 (4)	Er1—N3—C25—C26	−50.2 (5)
C1—C2—C3—Er1	57.8 (4)	C31—N3—C25—C30	−48.7 (6)
C2—C3—C4—C5	−2.1 (6)	Er1—N3—C25—C30	122.5 (4)
Er1—C3—C4—C5	−63.3 (4)	C31—N3—C25—Er1	−171.2 (5)
C2—C3—C4—Er1	61.2 (4)	C30—C25—C26—C27	−0.6 (7)
C3—C4—C5—C6	3.3 (6)	N3—C25—C26—C27	172.5 (4)
Er1—C4—C5—C6	−64.7 (4)	Er1—C25—C26—C27	137.5 (5)
C3—C4—C5—Er1	68.0 (4)	C25—C26—C27—C28	1.6 (8)
C4—C5—C6—C1	−1.2 (6)	C26—C27—C28—C29	−0.5 (8)
Er1—C5—C6—C1	−68.5 (4)	C27—C28—C29—C30	−1.7 (8)
C4—C5—C6—Er1	67.3 (4)	C28—C29—C30—C25	2.7 (7)
N1—C1—C6—C5	172.8 (4)	C26—C25—C30—C29	−1.6 (7)
C2—C1—C6—C5	−2.1 (6)	N3—C25—C30—C29	−174.4 (4)
Er1—C1—C6—C5	67.0 (4)	Er1—C25—C30—C29	−113.1 (5)
N1—C1—C6—Er1	105.9 (4)	C25—N3—C31—C36	150.8 (4)
C2—C1—C6—Er1	−69.1 (3)	Er1—N3—C31—C36	−16.4 (7)
C1—N1—C7—C12	124.5 (4)	C25—N3—C31—C32	−32.3 (6)
Er2—N1—C7—C12	−42.3 (5)	Er1—N3—C31—C32	160.5 (3)
C1—N1—C7—C8	−59.9 (5)	N3—C31—C32—C33	−179.0 (4)
Er2—N1—C7—C8	133.3 (4)	C36—C31—C32—C33	−2.0 (7)
C12—C7—C8—C9	1.5 (7)	C31—C32—C33—C34	1.9 (7)
N1—C7—C8—C9	−174.3 (4)	C32—C33—C34—C35	−0.6 (8)
C7—C8—C9—C10	−0.4 (7)	C33—C34—C35—C36	−0.6 (8)
C8—C9—C10—C11	−0.3 (8)	C34—C35—C36—C31	0.4 (7)
C9—C10—C11—C12	0.0 (8)	N3—C31—C36—C35	177.9 (4)
C8—C7—C12—C11	−1.8 (6)	C32—C31—C36—C35	0.9 (7)
N1—C7—C12—C11	173.9 (4)	C43—N4—C37—C38	−144.9 (4)
C10—C11—C12—C7	1.1 (7)	Er2—N4—C37—C38	41.8 (4)
C19—N2—C13—C18	174.3 (4)	C43—N4—C37—C42	38.1 (6)
Er1—N2—C13—C18	−48.5 (4)	Er2—N4—C37—C42	−135.2 (4)
Er2^i^—N2—C13—C18	48.7 (6)	C42—C37—C38—C39	−1.2 (6)
C19—N2—C13—C14	−16.4 (6)	N4—C37—C38—C39	−178.4 (4)
Er1—N2—C13—C14	120.8 (4)	C37—C38—C39—C40	0.5 (7)
Er2^i^—N2—C13—C14	−142.0 (4)	C38—C39—C40—C41	0.3 (7)
C19—N2—C13—Er1	−137.2 (4)	C39—C40—C41—C42	−0.3 (7)
Er2^i^—N2—C13—Er1	97.2 (4)	C40—C41—C42—C37	−0.5 (7)
C18—C13—C14—C15	0.5 (6)	C38—C37—C42—C41	1.3 (6)
N2—C13—C14—C15	−168.8 (4)	N4—C37—C42—C41	178.3 (4)
Er1—C13—C14—C15	−93.8 (5)	C37—N4—C43—C44	32.4 (6)
C13—C14—C15—C16	0.3 (7)	Er2—N4—C43—C44	−155.8 (3)
C14—C15—C16—C17	−0.6 (7)	C37—N4—C43—C48	−152.6 (4)
C15—C16—C17—C18	−0.1 (7)	Er2—N4—C43—C48	19.2 (5)
N2—C13—C18—C17	169.1 (4)	C48—C43—C44—C45	1.0 (6)
C14—C13—C18—C17	−1.1 (6)	N4—C43—C44—C45	176.2 (4)
Er1—C13—C18—C17	128.5 (4)	C43—C44—C45—C46	−0.7 (7)
N2—C13—C18—Er1	40.6 (3)	C44—C45—C46—C47	−0.3 (7)
C14—C13—C18—Er1	−129.6 (4)	C45—C46—C47—C48	1.0 (7)
C16—C17—C18—C13	0.9 (7)	C46—C47—C48—C43	−0.7 (7)
C16—C17—C18—Er1	99.2 (5)	C44—C43—C48—C47	−0.3 (6)
C13—N2—C19—C20	142.4 (4)	N4—C43—C48—C47	−175.7 (4)
Er1—N2—C19—C20	22.0 (5)	C54—C49—C50—C51	−1.6 (9)
Er2^i^—N2—C19—C20	−70.6 (4)	C49—C50—C51—C52	1.6 (9)
C13—N2—C19—C24	−42.4 (6)	C50—C51—C52—C53	−0.5 (9)
Er1—N2—C19—C24	−162.8 (3)	C51—C52—C53—C54	−0.6 (8)
Er2^i^—N2—C19—C24	104.6 (4)	C50—C49—C54—C53	0.5 (9)
C13—N2—C19—Er2^i^	−147.0 (4)	C52—C53—C54—C49	0.6 (8)

Symmetry code: (i) −*x*+1, −*y*+1, −*z*+1.
